# Geology and taphonomy of a unique tyrannosaurid bonebed from the upper Campanian Kaiparowits Formation of southern Utah: implications for tyrannosaurid gregariousness

**DOI:** 10.7717/peerj.11013

**Published:** 2021-04-19

**Authors:** Alan L. Titus, Katja Knoll, Joseph J.W. Sertich, Daigo Yamamura, Celina A. Suarez, Ian J. Glasspool, Jonathan E. Ginouves, Abigail K. Lukacic, Eric M. Roberts

**Affiliations:** 1 Paria River District, US Bureau of Land Management, Kanab, UT, USA; 2 Department of Earth Sciences, Denver Museum of Nature and Science, Denver, CO, USA; 3 Department of Geosciences, University of Arkansas at Fayetteville, Fayetteville, AR, USA; 4 Department of Geology, Colby College, Waterville, ME, US; 5 Department of Earth and Environmental Sciences, James Cook University of North Queensland, Townsville, QLD, Australia

**Keywords:** Tyrannosauridae, Campanian, Cretaceous, Behavior, Taphonomy, Laramidia, Utah, Kaiparowits, Teratophoneus, Bonebed

## Abstract

Tyrannosaurids are hypothesized to be gregarious, possibly parasocial carnivores engaging in cooperative hunting and extended parental care. A tyrannosaurid (cf. *Teratophoneus curriei*) bonebed in the late Campanian age Kaiparowits Formation of southern Utah, nicknamed the Rainbows and Unicorns Quarry (RUQ), provides the first opportunity to investigate possible tyrannosaurid gregariousness in a taxon unique to southern Laramidia. Analyses of the site’s sedimentology, fauna, flora, stable isotopes, rare earth elements (REE), charcoal content and taphonomy suggest a complex history starting with the deaths and transport of tyrannosaurids into a peri-fluvial, low-energy lacustrine setting. Isotopic and REE analyses of the fossil material yields a relatively homogeneous signature indicating the assemblage was derived from the same source and represents a fauna living in a single ecospace. Subsequent drying of the lake and fluctuating water tables simultaneously overprinted the bones with pedogenic carbonate and structurally weakened them through wet-dry cycling. Abundant charcoal recovered from the primary bone layer indicate a low temperature fire played a role in the site history, possibly triggering an avulsion that exhumed and reburied skeletal material on the margin of a new channel with minimal transport. Possible causes of mortality and concentration of the tyrannosaurids include cyanobacterial toxicosis, fire, and flooding, the latter being the preferred hypothesis. Comparisons of the RUQ site with other North American tyrannosaur bonebeds (Dry Island-Alberta; *Daspletosaurus horneri*-Montana) suggest all formed through similar processes. Combined with ichnological evidence, these tyrannosaur mass-burial sites could be part of an emerging pattern throughout Laramidia reflecting innate tyrannosaurid behavior such as habitual gregariousness.

## Introduction

**Figure 1:**
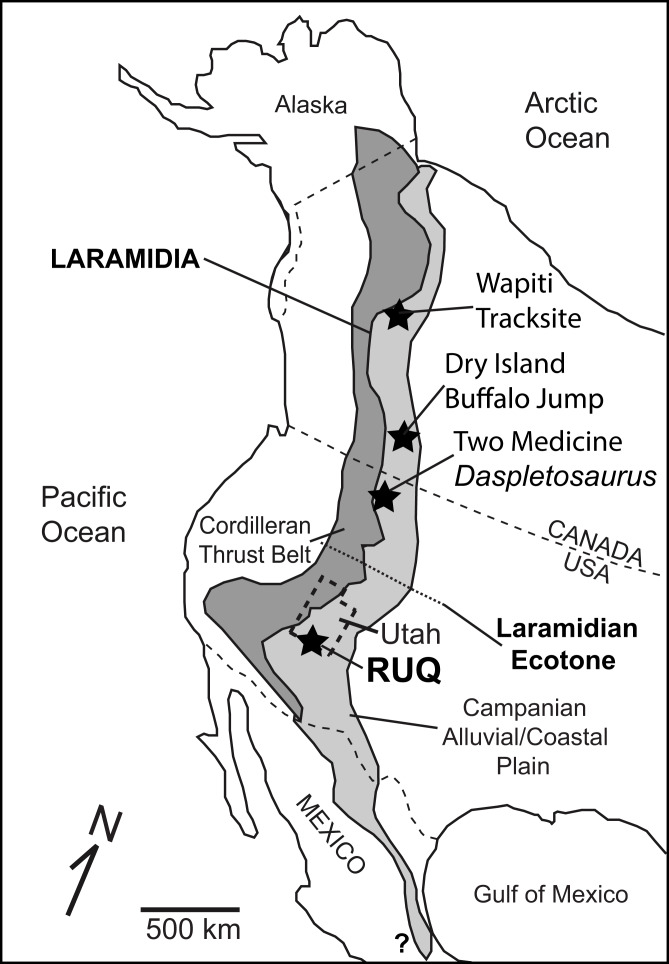
Overview map of Laramidia. Reference map for North America showing the ancient Laramidian land mass and locations of the Dry Island, Two Medicine, and Rainbows and Unicorns Quarry (RUQ) tyrannosaurid mass mortality sites. Also shown is the Wapiti Formation tyrannosaurid tracksite in northern British Columbia and a hypothesized ecotone between northern and southern Laramidia.

Monodominant large theropod dinosaur bonebeds are rare in the geologic record. Published examples include the Lower Jurassic *Dilophosaurus wetherilli* type locality (Welles, 1954), the Upper Jurassic Cleveland-Lloyd *Allosaurus fragilis*-dominated site in central Utah (Madsen, 1976; Gates, 2005; Peterson et al., 2017), the middle Cretaceous *Mapusaurus* locality in Neuquen, Argentina (Coria & Currie, 2006), the Upper Cretaceous MAD05-42 *Majungasaurus* quarry in Madagascar (Ratsimbaholison, Felice & O’Connor, 2016), the middle Cretaceous tyrannosauroid *Yutyrannus* site in China (Xu et al., 2012), and two tyrannosaurid sites from North America (Fig. 1): the Dry Island Buffalo Jump *Albertosaurus sarcophagus* site in the lower Maastrichtian Horseshoe Canyon Formation of Alberta, Canada (Eberth & Currie, 2010), and the *Daspletosaurus horneri* site (TA 1997.002) in the upper Campanian portion of the Two Medicine Formation of central Montana (Currie et al., 2005; Carr et al., 2017). Previous analyses of the two North American tyrannosaurid sites concluded they both possibly represent mass mortality of an aggregation and are not time-averaged or catastrophically-forced (Currie, 1998; Currie & Eberth, 2010). Perhaps the most controversial inference made from the Dry Island site is that it evidences habitual gregarious behavior including cooperative hunting (Currie, 1998; Currie & Eberth, 2010), something previously hypothesized only for tyrannosaur’s smaller paravian cousins (Maxwell & Ostrom, 1995).

Such monotaxic or monodominant bonebeds have historically been one of the few accepted arguments for gregariousness in fossil species (Currie & Eberth, 2010). However, their origins are frequently complex, and drought, fire, or other catastrophic events can force aggregations of normally non- or minimally-gregarious taxa (Currie & Eberth, 2010; Gates, 2005). Attritional traps such as tar seeps (Stock, 1972), quicksand/mires (Kirkland et al., 2016), or sinkholes (Martin & Gilbert, 1978) can also accumulate non-time associated individuals.

Here we describe a recently discovered large, monodominant tyrannosaurid bonebed containing at least four individuals from the upper Campanian Kaiparowits Formation of southern Utah. The size, fossil concentration, taxonomic diversity and geologic context of the site are unusual for Campanian terrestrial deposits in southern Laramidia, and the site’s taphonomy provides key insights into the local paleoclimate and environmental succession that led to its formation and preservation. The site also provides an opportunity to investigate whether the tyrannosaurids represent a time-averaged or forced accumulation, or another possible example of gregariousness within a completely different spatial, ecological and phylogenetic context south of the mid-Laramidia ecotone (Fig. 1) hypothesized by Loewen et al. (2013).

## Site discovery

**Figure 2:**
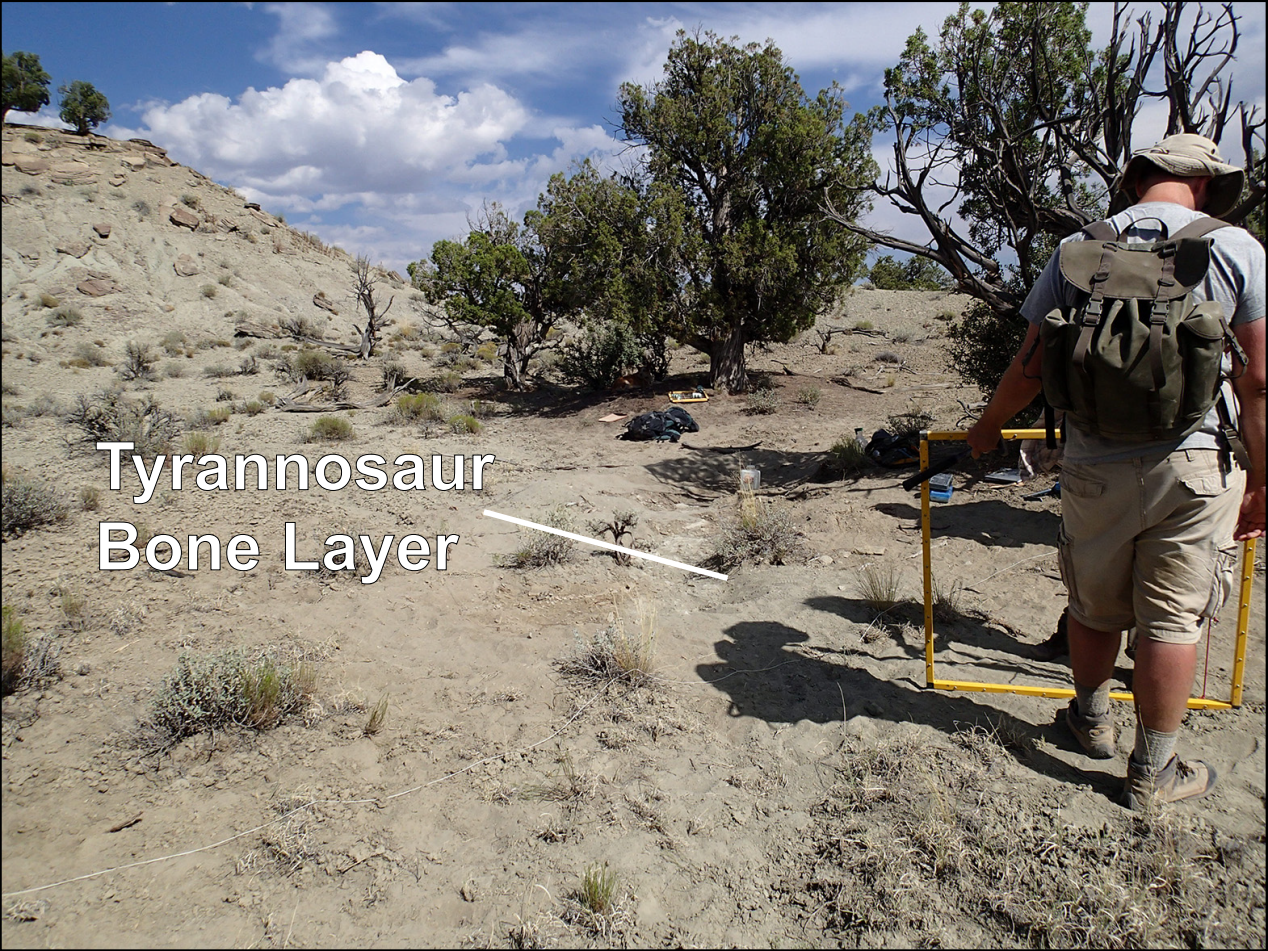
Photograph of the Rainbows and Unicorns Quarry. Photograph of the Rainbows and Unicorns Quarry tyrannosaurid mass-mortality site (14UTKA-8) on the day of discovery. Tyrannosaurid bones were first found eroding out the small rill indicated by the white line. Dr. Michael Knell holding a one-meter grid for scale.

**Figure 3:**
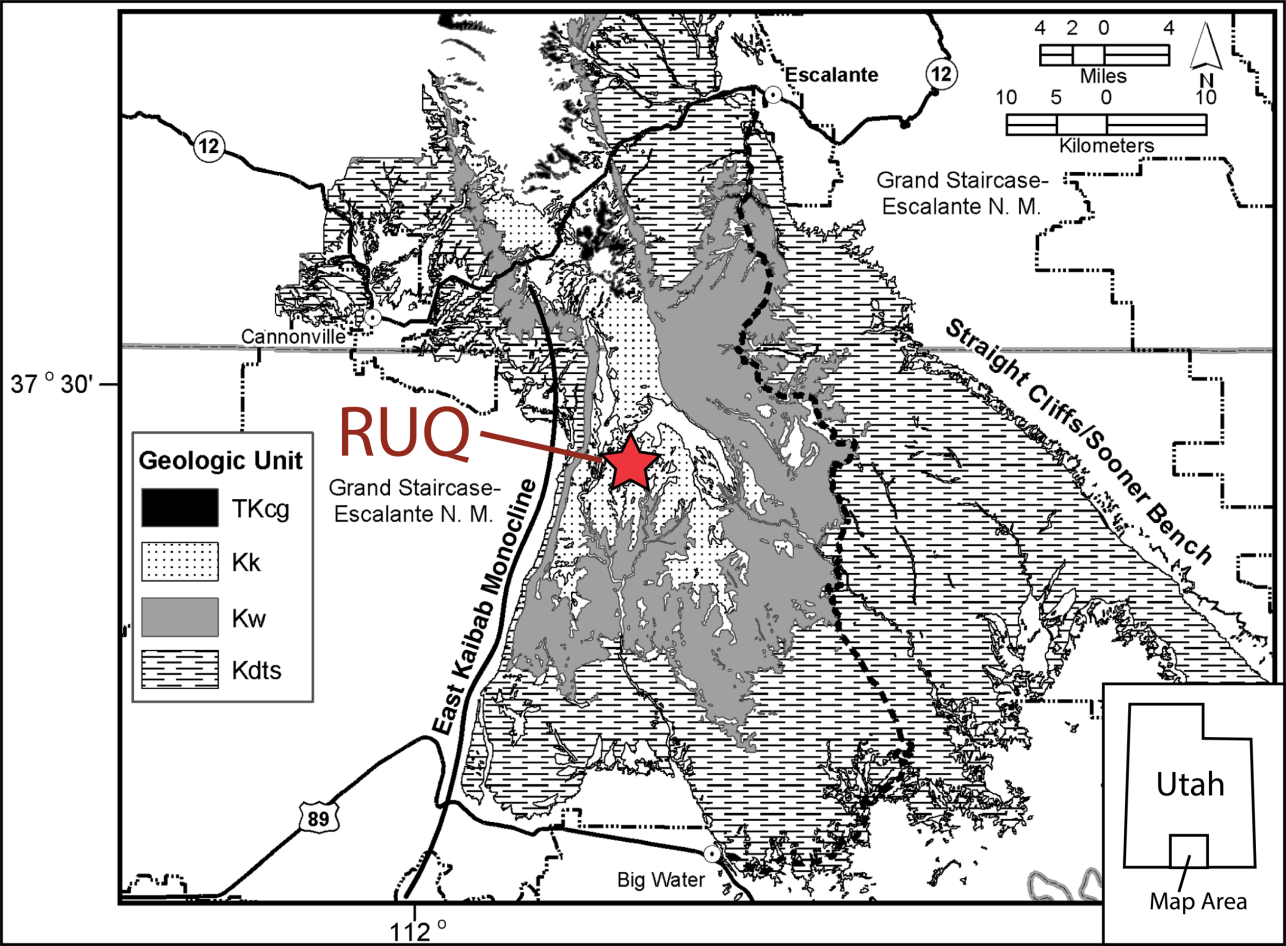
Reference geologic map of the Kaiparowits Plateau. Reference geologic map of the Kaiparowits Plateau region showing Cretaceous bedrock units and location of the Rainbows and Unicorns Quarry (RUQ). TKcg = Canaan Peak and Grand Castle Formations; Kk = Kaiparowits Formation; Kw = Wahweap Formation; Kdts = Dakota (now referred to the Naturita Formation), Tropic Shale and Straight Cliffs formations. N.M. = National Monument. Monument boundary is as of 2016 and does not reflect the current boundary which was altered in 2017.

In July 2014, the senior author (ALT) took MJ Knell and Katja Knoll (KK) to a small un-named butte (Fig. 2) in the northern Kaiparowits Plateau area (Fig. 3) of Grand Staircase-Escalante National Monument to assess some turtle remains for scientific potential. A few dozen meters northwest of two large *Neurankylus* turtle carapaces, ALT found a recently exposed single large tyrannosaurid astragalus. Preliminary investigation uncovered dozens of tyrannosaurid elements representing at least two individuals, a juvenile and a large adult. A new locality number (14UTKA-8) was assigned to the site and ongoing excavation was initiated at the end of July 2014.

By the end of the 2014 field season it became clear the site was one laterally persistent bonebed with a minimum extent of half a hectare. Because of the unusual concentration of large bones and high taxonomic diversity, it was nicknamed the “Rainbows and Unicorns Quarry” (RUQ). Detailed location information including UTM coordinates are on file at the Natural History Museum of Utah, Salt Lake City, and can be gained through requests to that institution’s Paleontology Collections Manager.

## Site geology

**Figure 4:**
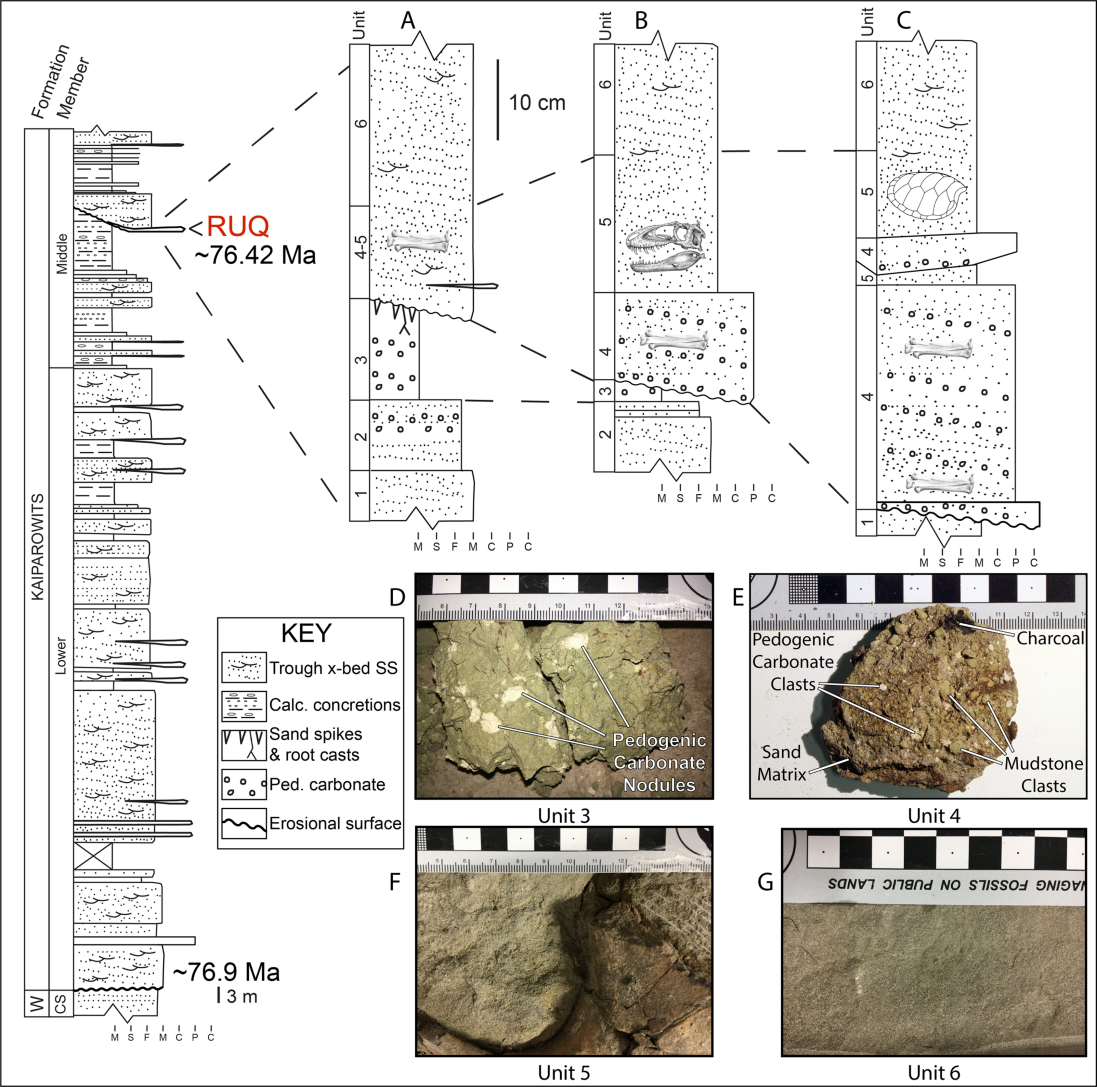
Graphic measured sections and photographs of major rock unit types for the RUQ. Left is of the lower portion of the Kaiparowits Formation from the RUQ area showing the stratigraphic position of the quarry and extrapolated absolute age. (A), (B) and (C) are detailed sections from the quarry whose locations are shown in Fig. 7 and whose unit numbers correspond with samples in the photographs. (D) Hand sample of unit 3 showing gleyed color and white pedogenic carbonate masses. (E) Hand sample of unit 4 showing every major lithic component of the unit except fossils. (F) Unit 5 exposed in fossil jacket during preparation. Marks in upper right are from an airscribe tool. A large piece of turtle carapace is visible in the lower right. (G) Medium grained sandstone of unit 6. Abbreviations are as follows: W = Wahweap; CS = capping sandstone; x-bed SS = cross-bedded sandstone; Calc. = calcareous; Ped. = pedogenic. Grain size abbreviations are; m = mudstone; s = siltstone; f = fine sandstone; m = medium sandstone; c = coarse sandstone; p = pebble; c = cobble.

**Formation and Age:** The RUQ is in the lower portion of the terrestrial upper Campanian Kaiparowits Formation, an 1,005-m thick succession of drab gray, gray-green and bluish-gray stream channel, channel margin, levee and floodplain sandstones, shales and minor intraformational conglomerates deposited in the western portion of the Sevier Foreland Basin between 76.9 Ma and 72.8 Ma (Roberts et al., 2013; Beveridge, Roberts & Titus, 2020). Climate indicators in the Formation reflect an overall wet (humid), tropical to subtropical wet-dry setting, with episodic longer dry spells. The lower 750 m of the Kaiparowits Formation is divided into the informal lower sandstone, middle mudstone and upper sandstone members (Roberts, 2007), in turn capped by the recently named 255-m-thick Upper Valley Member (Beveridge, Roberts & Titus, 2020). A section was measured to the RUQ from the top of the Wahweap Formation by ALT and EMR in August 2018 (Fig. 4) which determined it lies approximately 138.5 m above the base of the Kaiparowits Formation, or about 26 m above the base of the informal middle member. TIMS U-Pb dating of zircons in ash bed KP-07, located within 50 m above the bone horizon, yielded a date of 76.26 +/− 0.10 Ma (Roberts et al., 2013). Extrapolation using a depositional rate of 2.8 million years for the lower 865 m of Kaiparowits Formation (309 m/1 Ma) would, assuming a constant rate, suggest that the RUQ site is older than ash KP-07 by approximately 160,000 years (76.42 Ma-Fig. 4).

**Figure 5:**
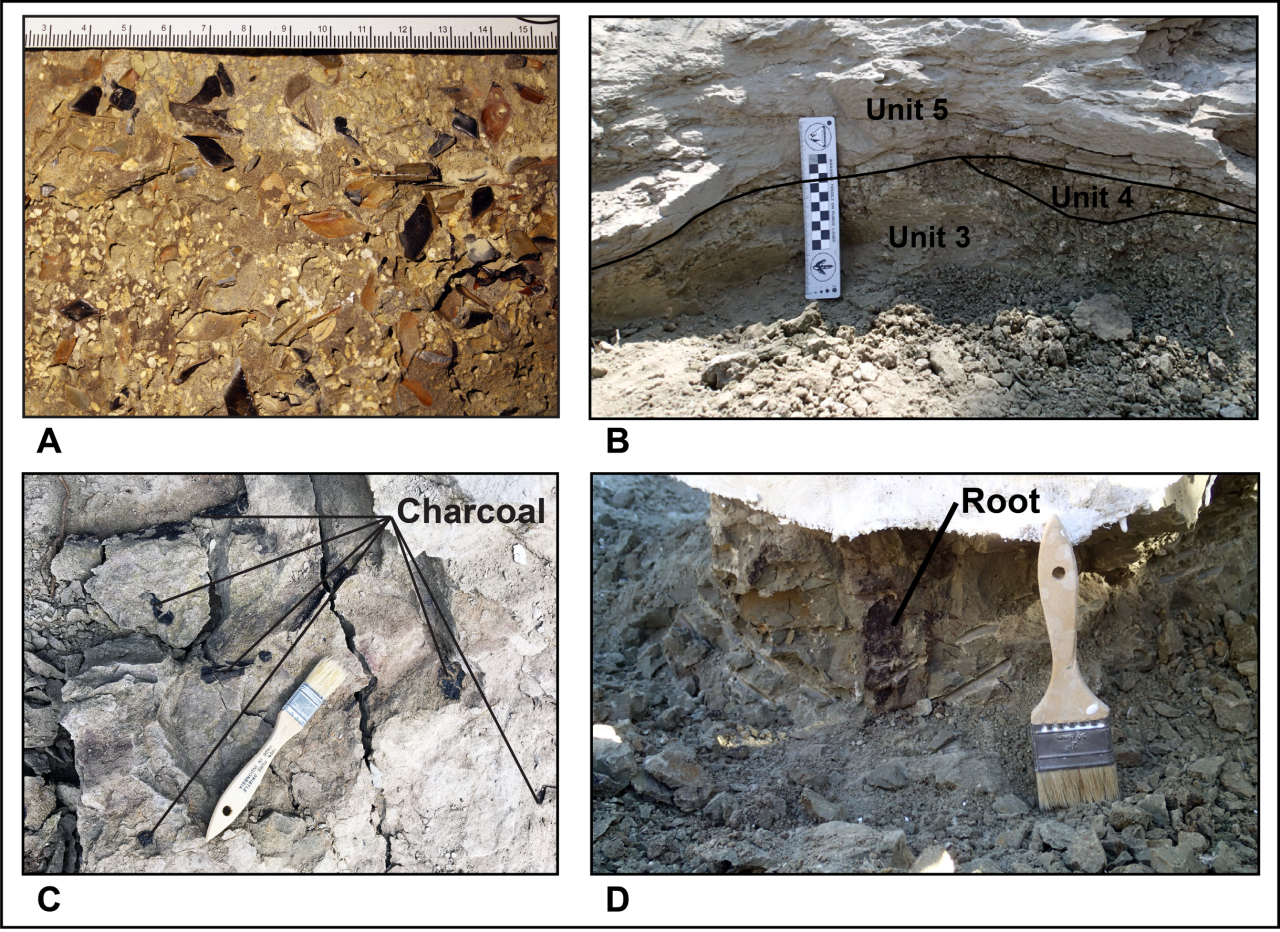
Photos of RUQ geologic features. (A) Concentration of gar scales and bones (referable to *Lepisosteus* sp.) at the base of unit 4. (B) Cross-sectional view showing architectural relationships of RUQ units 3, 4 and 5 and erosional relief imposed on unit 3. Taken at east edge of main tyrannosaurid bone area, just north of where section (B) in Fig. 4 was measured. Scale bar divided into cm. (C) Charcoal specimens in unit 5 from near section Fig. 4B. (D) 6 cm diameter carbonized root and associated mold in unit 3 below fossil jacket capping units 4 and 5. From near section (B). Brush is 50 mm wide for scale.

**Site Stratigraphy**: The oldest strata at the site consists of poorly fossiliferous to unfossiliferous gray-green (Munsell Gley 1 6/5GY) medium-to-fine sandstone (Fig. 4-units 1–2) overlain by a variable thickness of fossiliferous silty/sandy mudstone (Fig. 4D-unit 3). Unit 1 is a minimum 1-m-thick greenish-gray (Munsell Gley 1 6/5GY) horizontally bedded medium sandstone. Unit 2 is essentially a finer grained version of unit 1 that is part of a distinct fining upward trend but contains 5–20 mm diameter punky, porous carbonate nodules. Unit 3 is sandy mudstone with 1–2 cm long gastropods, small (1–20 mm) dispersed carbonate nodules (Fig. 4D) like those in unit 2, uncommon bone fragments and turtle shell and is locally penetrated by vertically oriented carbonized root remains (Fig. 5D). XRD analysis (Table S1) of the bulk unit 3 fines show it to be a mixed siliceous mudstone using the terminology of Diaz, Camron & Lewis (2013) with the clay component having roughly equal chlorite (20%) and kaolinite (22%) content and higher levels of illite (58%). Based on onlapping/downcutting relationships with units 4–6 around the quarry’s north edge, it is estimated unit 3 and whatever other beds may have previously been present above it did not exceed 2 m thickness. The entire sub-unit 4 succession bears a pedogenic overprint of “greening” (=mild oxidation) of the presumed original gray color and would be classified as a calcic gleyed protosol following Tabor, Myers & Michel (2017).

**Figure 6:**
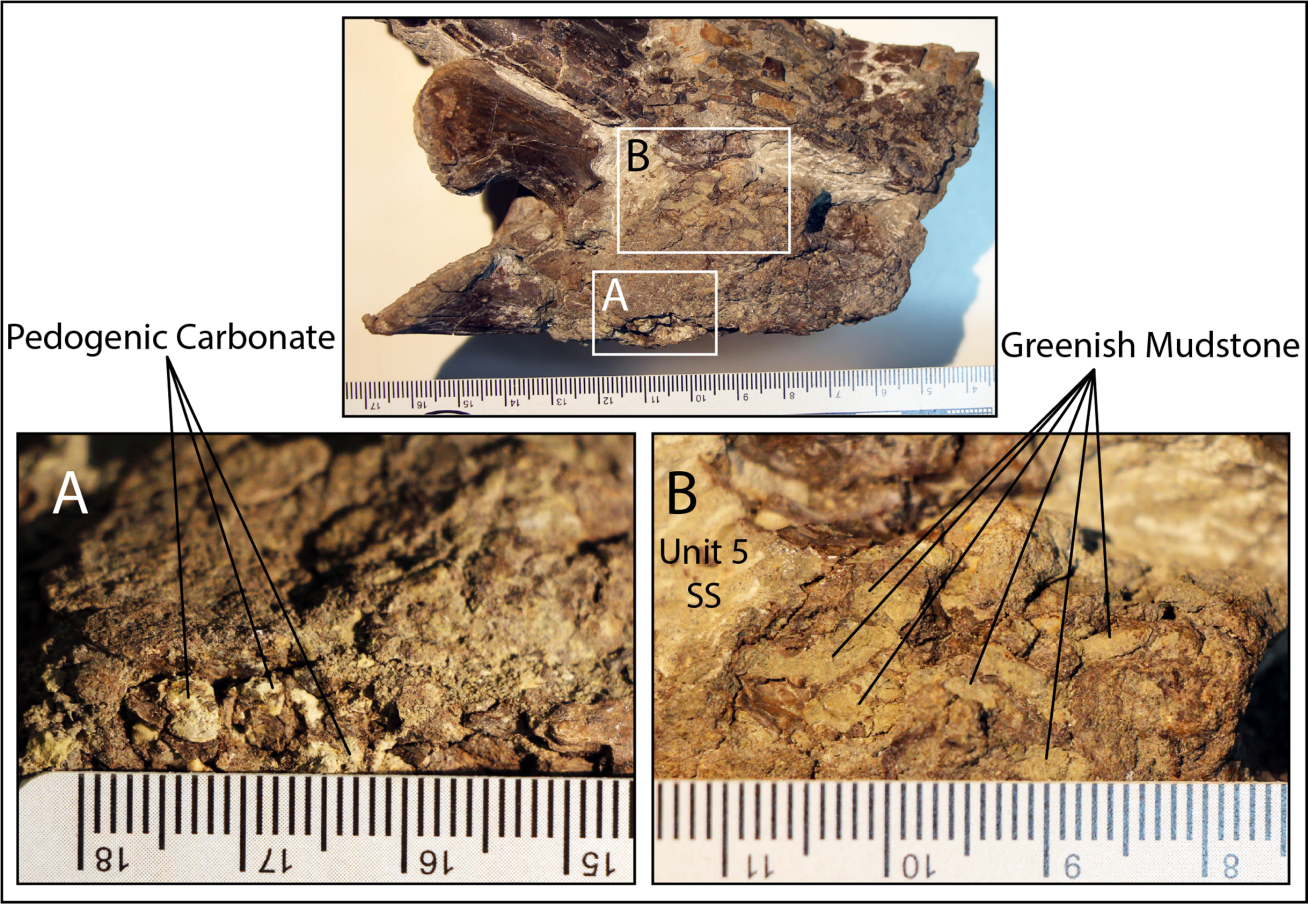
Details of juvenile tyrannosaur vertebra (#384) from unit 5. Both pedogenic carbonate (A) and green mudstone (B) infillings are visible inside pneumatic pore spaces. Mudstone infill is only observed inside small pore spaces of intact robust elements like vertebrae.

**Figure 7:**
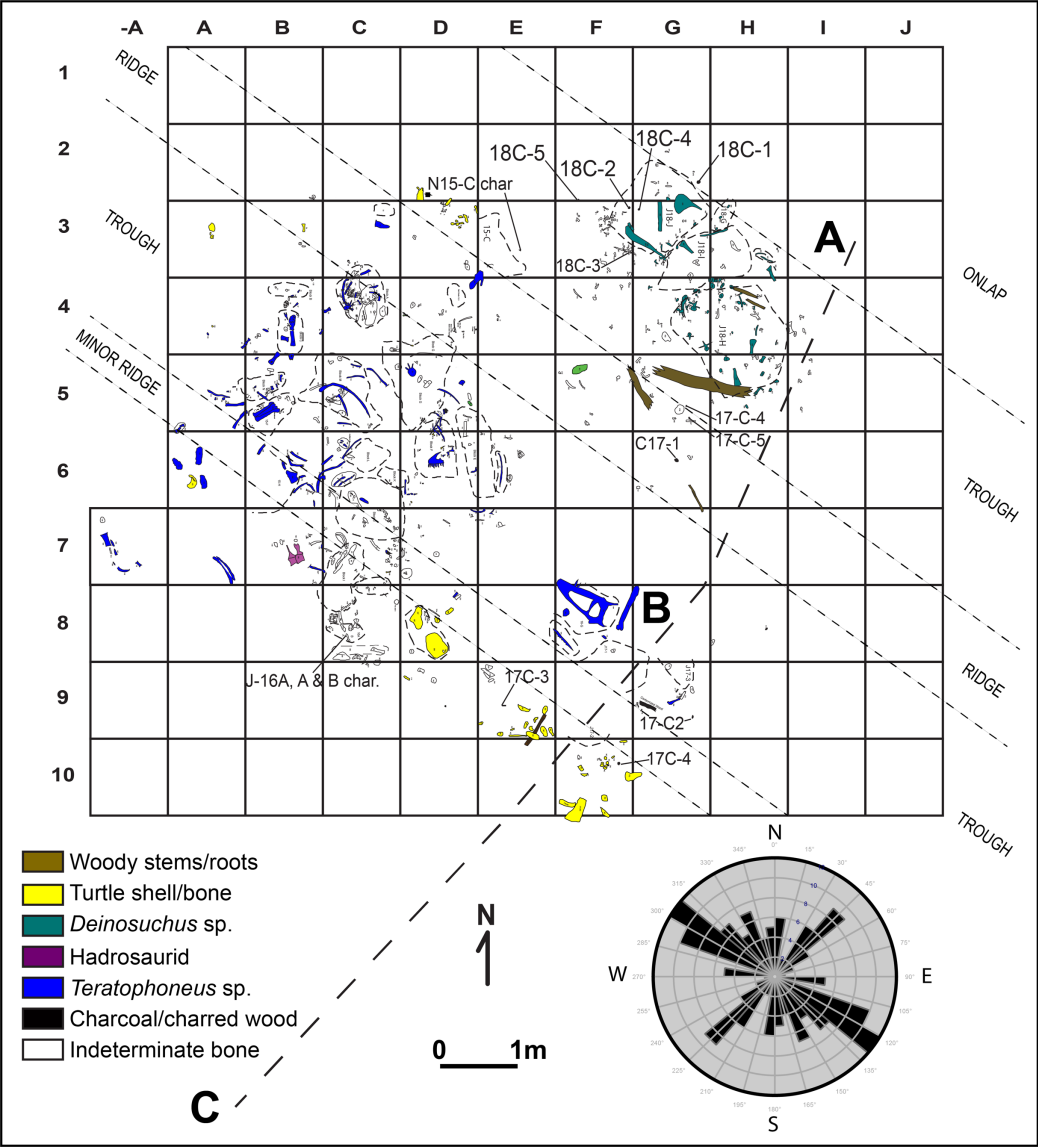
Quarry map and rose diagram for area of main tyrannosaurid accumulation at the RUQ. Also shown are sub-unit 4 surface features, source of charcoal samples, and location of detailed sections (Figs. 4A–4C). Dashed lines represent outlines of field jackets.

The main RUQ bonebed is an up to one-meter-thick collective sequence of conglomerate (Fig. 4E-unit 4) and sandstone (Fig. 4F-unit 5) that pinches out in a northeast direction and thickens to the southwest over an additional 1 m of erosional relief carved into units 1–2 (Figs. 4A–4C). Most clasts in the conglomerate consist of reworked off-white punky pedogenic carbonate nodules identical to those in units 2–3, green and gray colored clay rip-ups (Fig. 4E) and fish debris (Fig. 5A) supported by a gray feldspathic-lithic sand matrix (Munsell Gley 1 6/N). Clay clasts are generally greenish gray (Munsell Gley 1 6/5GY), but some are gray (Munsell Gley 1 7/N), and range in size from 2 mm to 20 mm. The clay/carbonate clast ratio is locally highly variable but is usually subequal. Clay clast bulk composition (Table S1) shows the green ones are mixed siliceous mudstone compositionally very similar to unit 3 (chlorite-kaolinite-illite = 22%, 23%, 55%), while the gray ones are slightly higher in carbonate, classifying as argillaceous siliceous mudstone with a slightly higher illite fraction (chlorite-kaolinite-illite = 16%, 10%, 74%). Most fish scales are concentrated near the bottom of unit 4 in a lag (Fig. 5A), which also hosts gastropod and bivalve steinkerns, carbonized roots, charcoal (Fig. 5C), other microvertebrates, and locally common macrovertebrate remains. Fractured tyrannosaur elements in units 4 and 5, have gray sandstone infillings identical to the matrix in which they reside. In stark contrast, pneumatic and vascular pore spaces inside structurally intact robust elements like vertebral centra, are filled with both precipitated (not clasts) off-white punky pedogenic carbonate (Fig. 6A) and green mudstone (Fig. 6B). Such mud-fills readily form early in the burial history of mud-hosted bone via hydrologic vacuums created by escaping decompositional gasses (Bodzioch, 2015) and indicate that RUQ tyrannosaur material was initially buried in greenish mudstone. Analysis of clay inside a RUQ tyrannosaur centra shows it to have the same kaolinite fraction, but slightly higher chlorite and lower illite levels than either unit 3 or the unit 4 green and gray clasts (Table S1). Seemingly, the constant kaolinite fraction and elevated chlorite and illite levels link all these clays genetically, with observed variations paralleling those in modern depositional settings with heterogeneous source areas (Griffiths et al., 2019). Regardless, the contrasting infill of intact elements demonstrates their primary depositional context was not units 4 and 5, but a pedogenic mudstone similar to unit 3. Unit 4 generally forms the base of the bone-bearing interval in lenses and sheets within the lower 30–50 cm of the bonebed, but does locally pinch out over small topographic ridges in the underlying scoured units (Fig. 5B) and at the north edge of the quarry (Fig. 7) where the whole succession onlaps underlying units and thins dramatically.

Extra-basinal chert and limestone pebble clasts, some bearing Paleozoic age marine fossils, are sparsely distributed through the quarry and may represent material washed in on tree or shrub stumps. However, one unusual cluster at the north end of the quarry, closely associated with a *Deinosuchus* skeleton, are very likely gastroliths. Distribution of fossil taxa appears to be random between units 4 and 5 (i.e., all fossil types are found in both units), with local variations in abundance. Unit 6, a gray (Munsell Gley 1 6/N) trough-crossbedded medium sandstone (Fig. 4G), represents a continuation of channel fill on top of the bone-bearing unit that is essentially unfossiliferous. We interpret units 4–6 as mixed lag debris and fill on the northern margin of a major (>0.3 km width) incised fluvial channel whose exact scale and geometry are impossible to determine because most of it was removed by Holocene erosion.

**Figure 8:**
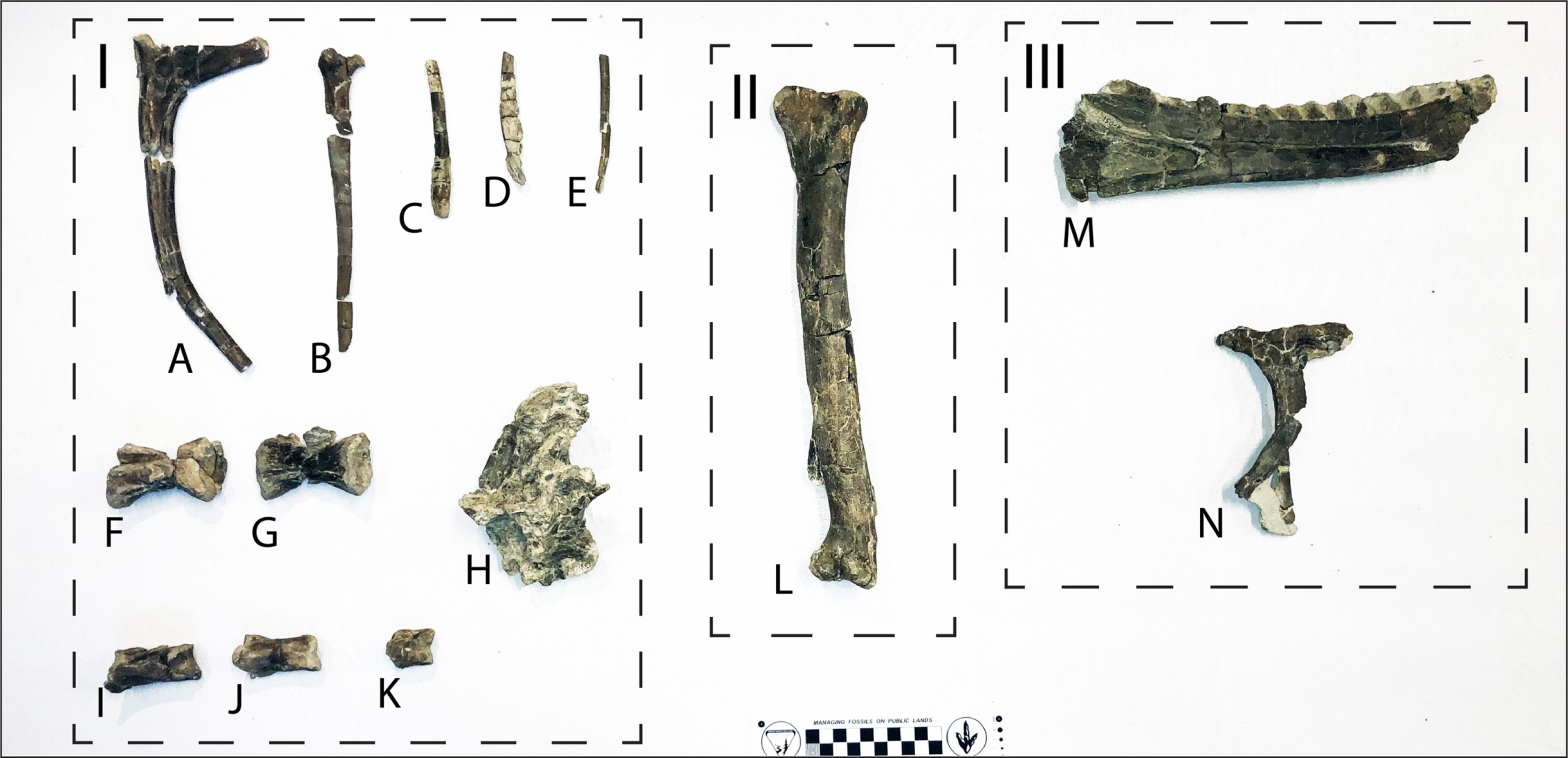
Photograph showing RUQ tyrannosaur elements of Voorhies groups I–III. (A–K) Group I. (A) Juvenile dorsal rib. (B) Juvenile cervical rib. (C–E) Juvenile gastralia. (F and G) Juvenile caudal vertebrae. (H) Juvenile dorsal vertebra. (I–K) Juvenile pedal phalanges. (L) Group II, juvenile tibia. (M and N) Group III. (M) Juvenile dentary. (N) Juvenile lacrimal. Scale bar is in centimeters.

**Paleocurrent Indicators:** Local troughs and ridges (fluting) roughly striking 314° are discernible in the main tyrannosaurid area (Fig. 7). Clear trough-crossbedding is absent in units 4 and 5, although lobate structures occur randomly throughout the quarry aligned with the underlying incisions into units 1–3 which we interpret as poorly-developed meso-scale trough-crossbedding. Bimodal orientation of long elements and vectors associated with scatters of individual animals (Fig. 7), as well as the lensoidal-lobate bedding structure (in units 4 and 5), and orientation of basal scour troughs suggest fluvial transport toward an azimuth of 314°. Unit 6, interpreted as channel fill lateral bar deposits (Fig. 4-unit 6), grades into an overlying fine-grained floodplain succession representing subsequent channel abandonment (not figured). Skeletal elements of nearly all taxa have multiple representatives (Fig. 8) in all three Voorhies groups (I–III of Voorhies, 1969) demonstrating low-to-moderate current strength and minimal transport. The onlapping relationship between unit 3 and units 4 and 5 leads us to presume low velocities resulted from proximity to the channel bank, possibly in a transitional zone between active cutbank erosion and point bar deposition.

## Biotic composition

**Figure 9:**
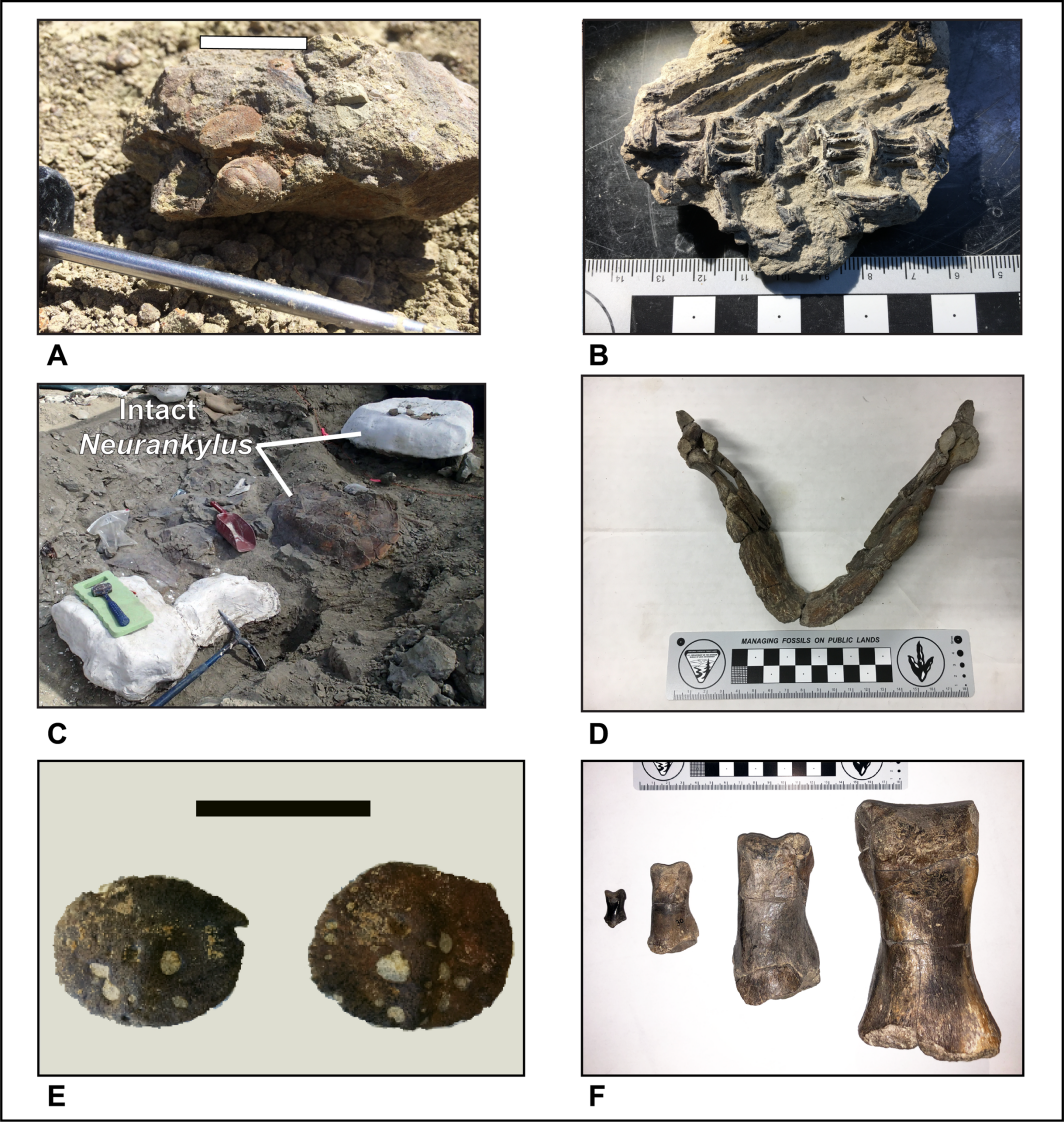
Faunal elements from units 4 and 5 at the RUQ site. (A) *Viviparus* sp. and an unidentified juvenile unionid (unit 4). (B) Articulated vertebrae of an unidentified teleost fish (unit 5). (C) Overview of area south of main tyrannosaurid accumulation with dense occurrence of turtle carapaces and skeletal material (unit 4). Two intact large *Neurankylus utahensis* Lively shells, one exposed, one jacketed are visible. (D) Mandible of unidentified giant panchelonioid turtle (unit 5). (E) Osteoderms of juvenile *Deinosuchus hatcheri* Holland, 1909 from northern portion of RUQ (unit 5). (F) Pedal phalanges of cf. *Teratophoneus* and a non-paravian coelurosaur grade theropod (likely a juvenile *Teratophoneus*) exhibiting three, possibly four different growth stages (units 4 and 5). Left to right: possible small juvenile III-3 (#728), juvenile right II-2 (#30), subadult right II-2 (#313), somatic adult III-1 (#21). Bar scales: A = 2 cm; E = 5 cm.

**Non-tyrannosaurid components:** Preserved macroflora in all units consists entirely of roots (and possibly woody stems) and charred wood; very unusual for Kaiparowits Formation bonebeds which normally produce woody stems and foliage. Identifiable charcoal is referred to Cupressaceae, Podocarpaceae and Pinaceae. Both unit 3 and 4 have yielded a low diversity gastropod assemblage (as reworked steinkerns) containing *Viviparus* (Fig. 9A) and *Lioplacodes*. The largest individuals are approximately 2.5 cm in height. Bivalves, consisting entirely of juvenile unionids less than 2 cm in length, occur only as steinkerns in unit 4 (Fig. 9A). The RUQ vertebrate assemblage, still being prepared and cataloged, is moderately diverse and dominated by aquatic taxa but includes several terrestrial forms. Based on absolute numbers of specimens it would be dominated by fish (represented mostly by scales), however, a strong collecting bias towards tetrapods leaves this faunal component underrepresented in this study. Lepisosteidae dominate (estimated 80%) recovered/observed specimens, with rays, amiids, sturgeons and yet unidentified, but possibly diverse teleosts (Fig. 9B) making up the remaining estimated 20%. Turtles are a significant component both numerically and taxonomically, with at least seven species from four different families represented. The most abundant taxon is *Neurankylus utahensis* Lively, 2015, with individual shells ranging in diameter from 0.5 m to 1 m. Most taxa are represented by both skeletons and shells, and while most shells are broken, local concentrations of large, whole shells do occur (Fig. 9C). The most remarkable turtle from the site is a large panchelonioid with an estimated >1.5 m diameter shell (Fig. 9D). Neosuchians are uncommon but represented by two isolated small teeth and a spectacular, relatively complete 4-m-long juvenile specimen of the alligatoroid *Deinosuchus* (Fig. 9E) associated with possible gastroliths.

Most aquatic fauna can be referred to either river, pond or lake environments (all smaller turtle taxa and most fish) although larger elements such as *Neurankylus* may have preferred riverine habitats (Knell, 2012). However, many modern sturgeons are anadromous, with all species confined to major river systems and connected large lakes (Billard & Lecointre, 2001), indicating this part of the RUQ fauna was washed in from a trunk stream during seasonal flooding. Although there are no modern observations of panchelonioids migrating great distances up major river systems, they also probably washed in from a major trunk stream. At least three dinosaur taxa have been identified: tyrannosaurids, an indeterminate hadrosaurid, and an indeterminate small paravian theropod. Although hadrosaur material is widespread at the site, all recovered elements (femur = 72 cm length, radius = 69 cm length, sacrum, vertebrae) suggest the presence of a single large juvenile/subadult. Two highly processed/weathered ornithischian limb fragments belong to an individual of unknown affinity. The paravian material is sparse, points to a single individual, and is found only in the north area of the quarry among the main concentration of tyrannosaur bones.

**Figure 10:**
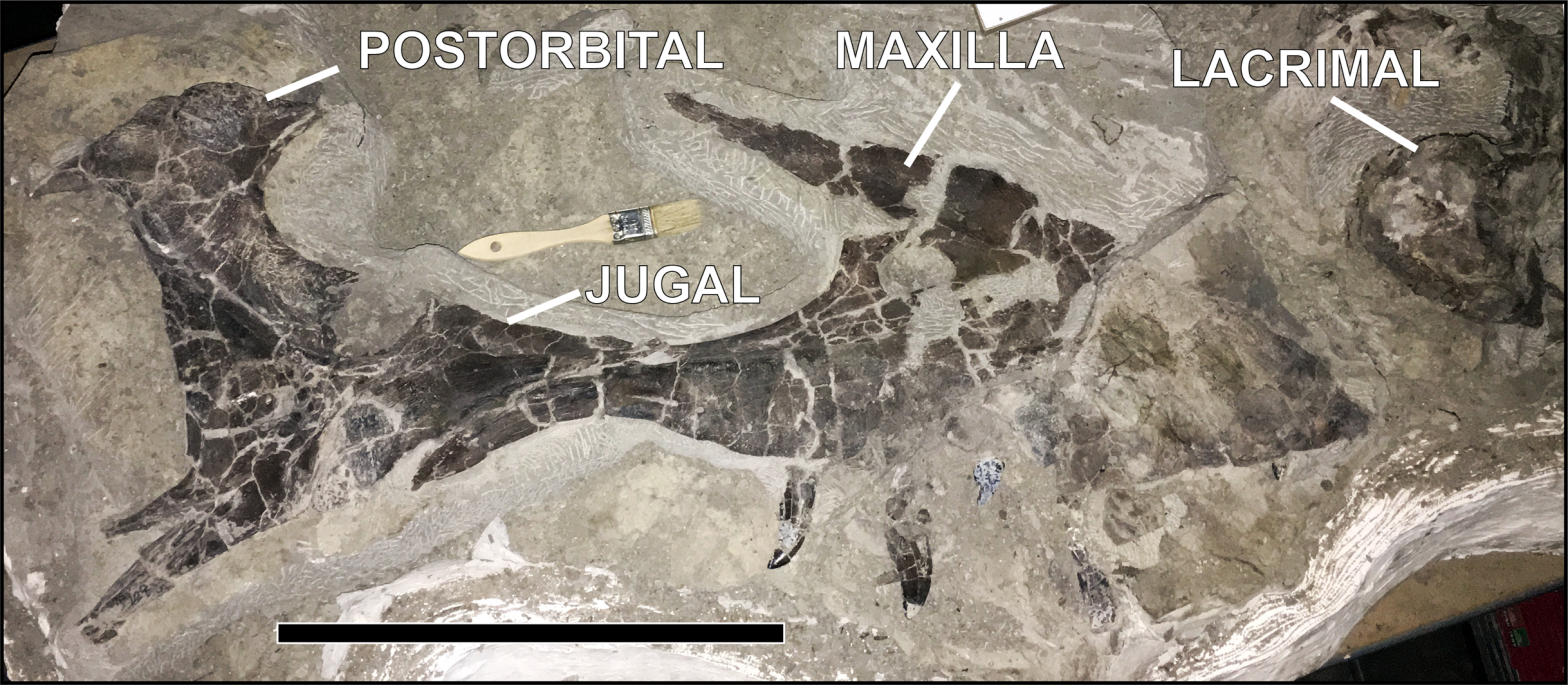
Adult cf. *Teratophoneus* partial skull complex (element #360) from contact between units 4 and 5 preserving the jugal, postorbital, and maxilla. Right lacrimal (element #375) is displaced slightly to the upper right. Bar scale = 40 cm.

**Tyrannosauridae:** Tyrannosaurid bones are concentrated in a 100 m^2^ area at the north edge of the site, with only a couple of elements recovered outside that area, immediately to the south. Comparison of lacrimals from the largest individual (Fig. 10) and a specimen 55% of that size shows they are both “L-shaped” (jugal process joins at a right angle), with the cornual processes projecting laterally rather than dorsally. Also, the postorbital morphologies of different size classes are nearly identical in overall shape, differing mostly in the degree of development of the cornual process. Furthermore, maxillae in the largest specimens and individuals in the 55% size class both have 13 alveoli, a count typical of southern Laramidia tyrannosaurids (Carr et al., 2011; Loewen et al., 2013) such as *Teratophoneus curriei* Carr et al. (2011). Accordingly, we refer all the RUQ individuals to a single taxon, cf. *Teratophoneus curriei* Carr et al. (2011).

In summary, the following vertebrate taxa have been identified from the RUQ:

Batomorphii

 Rhinobatoidea Family *incertae sedis*

  *Myledaphus bipartitus* Cope, 1876

Osteichthyes-Neopterygii

 Acipenseriformes

  Gen. et. sp. indet.

Amiidae

 *Melvius* sp.

Lepisosteidae

 *Lepisosteus* sp.

Teleostei

 Family *incertae sedis*

Testudines

 Baenidae

  *Neurankylus utahensis* Lively, 2015

  cf. *Boremys*

 Adocidae

  *Adocus* sp.

 Trionychidae

  *Helopanoplia* sp.

  *Aspideretoides* sp.

  ?*Derrisemys* sp.

  *Gilmoremys gettyspherensis* Joyce, Lyson & Sertich, 2018

 Panchelonioidea

  Gen. et sp. indet.

 Crocodylia

Alligatoroidea Family *incertae sedis*

 *Deinosuchus* sp.

Dinosauria-Saurischia

 Tyrannosauridae

  cf. *Teratophoneus curriei* Carr et al., 2011

 Paraves

  Gen. et sp. indet.

Dinosauria-Ornithischia

 Hadrosauridae

  Gen. et sp. indet.

## Geochemical analysis

### Stable isotopes

**Methods:** Pedogenic calcium carbonate (calcite) nodules both from in situ (unit 3) and *ex situ* (unit 4) sources were analyzed for stable carbon (δ^13^C_CO3_) and oxygen (δ^18^O_CO3_) isotopes in order to characterize the site’s paleohydrology and determine if the unit 4 pedogenic nodules were locally derived from unit 3 or related strata. Pedogenic nodules from unit 3 consisted of both micrite and fracture-fill calcite spar components that were analyzed separately and together in bulk. Nodules from unit 4 were homogeneous micrite and were analyzed in bulk. All carbonate samples were digested in phosphoric acid at room temperature and analyzed via a gas bench II attached to a Thermo Advance Plus isotope ratio mass spectrometer (IRMS) at the University of Arkansas Stable Isotope Laboratory (UASIL).

Shells of five different turtle taxa randomly sampled from units 4–5 (a large trionychid = *Aspideretoides*, a small trionychid = *Gilmoremys gettyspherensis*, a medium-sized baenid = cf. *Boremys*), a large baenid = *Neurankylus utahensis* and the giant panchelonioid) were also analyzed for δ^18^O_p_ to determine their ecological (aquatic) fingerprints specifically to try and falsify our assumption they were all derived locally from the same facies/strata. Silver phosphate samples from turtle specimens were reacted with glassy carbon chips at 1400 °C on a thermo high temperature conversion elemental analyzer (TC/EA) attached to a continuous flow Advance Plus IRMS at UASIL.

Isotopic composition of water was calculated from turtle remains using the equation of Barrick, Fischer & Showers (1999) revised by Coulson et al. (2008):

δ^18^O_w_ = 1.08δ^18^O_p_ – 22.3, while isotopic composition of water derived from carbonate nodules was based on temperature estimates using clumped isotopes (Burgener et al., 2019) and plant Leaf Margin Analysis (LMA) (Miller et al., 2013). We used the temperature dependent isotopic fractionation factor of O’Neil, Clayton & Mayeda (1969), to estimate isotopic composition of meteoric water:

1000 ln*a* = 3.23(10^6^/*T*^2^) − 3.5

δ^18^O_w_ = (δ^18^O_c_ + 1000)/*a* α − 1000

All carbonate values are reported as ‰VPDB, while all phosphate values are ‰VSMOW.

**Results:**

**Figure 11:**
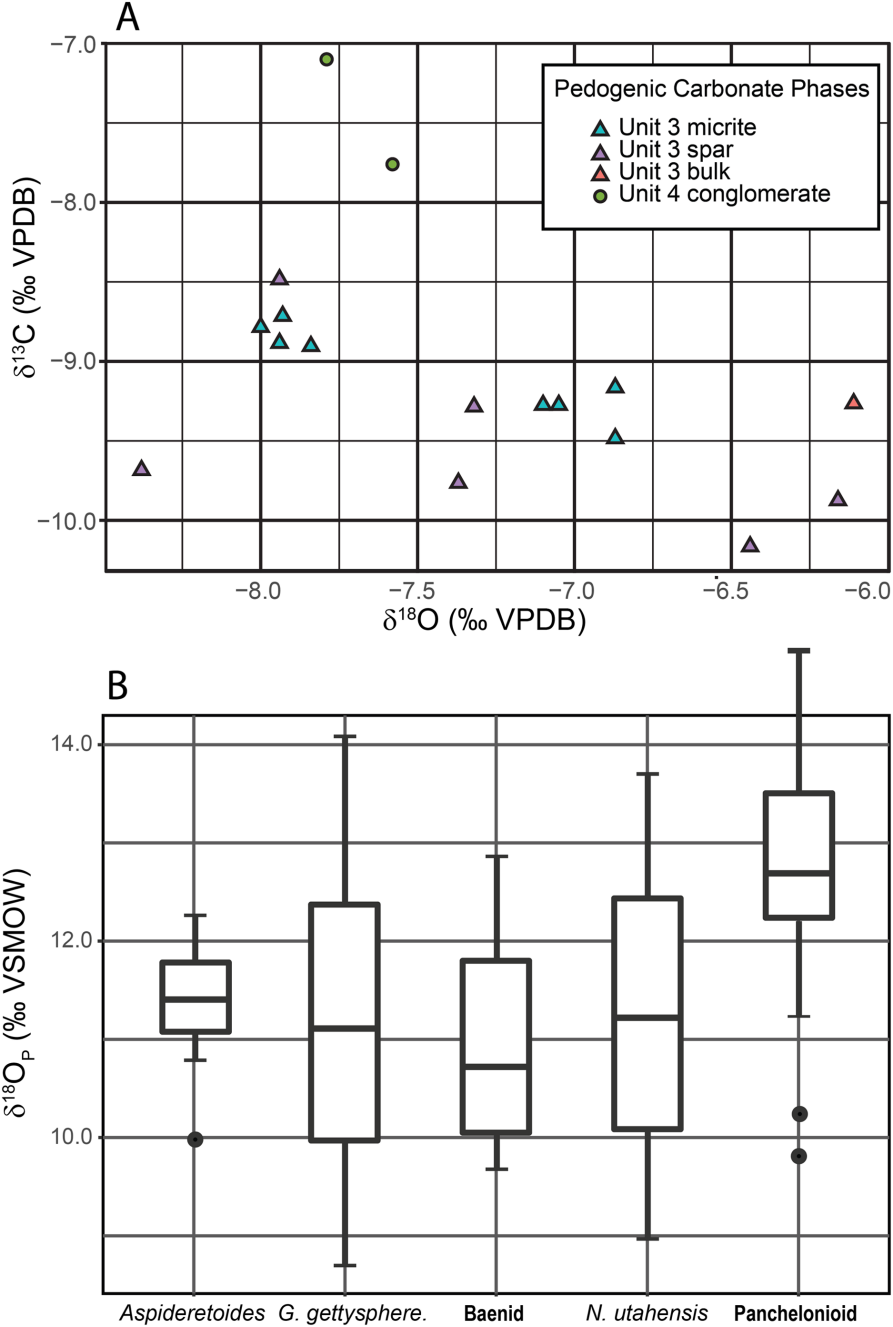
Plots of stable isotope data. (A) C–O isotope cross-plot of pedogenic carbonate nodules. Unit 3 in-situ nodules contain both primary micrite yielding tightly clustered results and sparry calcite veins with more widely scattered results that bias the bulk (unit 3 bulk) results towards a heavier average. Unit 4 conglomerate nodules are indistinguishable from those in unit 3 in δ^18^O_CO3_ space but are heavier in δ^13^C_CO3_ space. (B) Box plot of turtle δ^18^O_p_. Solid line represents median δ^18^O_p_, box boundary represents third and fourth quantile, whiskers represent maximum and minimum and points represent likely outliers. Abbreviation: *G*. = *Gilmoremys*; *N*. = *Neurankylus*.

Pedogenic Carbonate.—Results are given in Table 1, Table S2 and Fig. 11A. Bulk (spar + micrite) mean δ^13^C, for the unit 3 calcite nodules is −9.26‰ (*n* = 14, SD = 0.76). The mean δ^13^C for the micritic component of the nodules is −9.06‰ (*n* = 8, SD = 0.28) while the mean for the sparry calcite is −9.54‰ (*n* = 6, SD = 0.59). Bulk (spar + micrite) mean δ^18^O_CO3_ for unit 3 calcite nodules is −7.37‰ (*n* = 14, SD = 0.70). The mean δ^18^O for the micritic component is −7.44‰ (*n* = 8, SD = 0.53) while that for the sparry calcite is −7.27‰ (*n* = 6, SD = 0.88). For conglomerate-derived micrite nodules (unit 4), mean δ^13^C is −7.43‰ (*n* = 2, SD = 0.47), and mean δ^18^O is −7.73‰ (*n* = 2, SD = 0.16).

**Table 1:** Averaged pedogenic carbonate nodule isotopic composition data.

Nodule type	Unit	*n*	δ^18^O_CO3_ VPDB	1σ δ^18^O_CO3_	δ^13^C_CO3_	1σ δ^13^C_CO3_
Bulk (micrtic + sparry mixed)	3	14	−7.37	0.7	−9.26	0.76
Micritic	3	8	−7.44	0.53	−9.06	0.28
Sparry	3	6	−7.27	0.88	−9.54	0.59
Micritic (conglomerate)	4	2	−7.73	0.16	−7.43	0.47

Turtles.— A summary of data is shown in Table 2 and Fig. 11B. The δ^18^O_P_ for the turtles ranges between 12.8 ± 1.3 (1σ) for the giant panchelonioid to 11.0 ± 1.4‰ for an unnamed baenid. *Neurankylus utahensis* averages δ^18^O_P_ = 11.3 ± 2.4‰, while *Aspideretoides* δ^18^O_P_ = 11.3 ± 0.7‰, and *Gilmoremys gettyspherensis* δ^18^O_P_ = 11.1 ± 2.3‰. The four non-panchelonioid taxa produced δ^18^O_P_ values statistically indistinguishable from each other, while the panchelonioid is slightly heavier (Fig. 11B).

**Table 2:** Averaged turtle phosphate oxygen isotopic composition data.

Taxon	*n*	Median δ^18^O_P_ VSMOW	Avg. δ^18^O_P_ VSMOW	1σ δ^18^O_P_
*Aspideretoides*	8	11.4	11.3	0.7
*Gilmoremys*	4	11.2	11.1	2.3
Baenid	4	10.8	11.0	1.4
*Neurankylus utahensis*	3	11.2	11.3	2.4
Large panchelonioid	23	12.7	12.8	1.3

Water Isotopic Composition.—Water isotopic composition was calculated from both turtles and pedogenic carbonate nodules (Table 3) to determine if the meteoric water source for the different carbonate nodules in units 3 and 4 was the same and to compare them to water that turtles consumed and lived in. For carbonate nodules, temperature must be assumed, and we used temperature averages derived from both clumped isotope analysis of Burgener et al. (2019) (35 ± 4 °C), and LMA of Miller et al. (2013) (20 ± 1 °C). Given the lack of significant difference between the different calcite phases of unit 3 carbonate nodules we use a bulk δ^18^O_co3_ value of −7.37‰. With these average values, the isotopic composition of water that precipitated unit 3 nodules at 35 °C was −6.40‰ and the isotopic composition of water that precipitated nodules re-worked into unit 4 was −6.76‰. Using 20 °C, water isotopic composition was −9.83‰ for bulk unit 3 nodules and −10.18‰ for unit 4 transported nodules. Given the low variability of different turtle shell δ^18^O_p_ we averaged all turtle δ^18^O_p_ values. The δ^18^O_w_ that turtles lived in was −11.21‰.

**Table 3:** Summary of calculated water oxygen isotopic composition values (δ^18^O_w_) for both carbonate and turtle phosphate.

Mineral	Unit	Temp	α	δ^18^O_w_ VSMOW
Bulk unit 3	3	35	1.029901	−6.40
Bulk unit 3	3	20	1.03347	−9.83
Unit 4	4	35	1.029901	−6.76
Unit 4	4	20	1.03347	−10.19
Average large panchelonioid	5	–	–	−9.4
Average turtles	5	–	–	−11.2

**Note:**

Results are based on MAT data from Miller et al. (2013) and clumped isotope work of Burgener et al. (2019).

**Figure 12:**
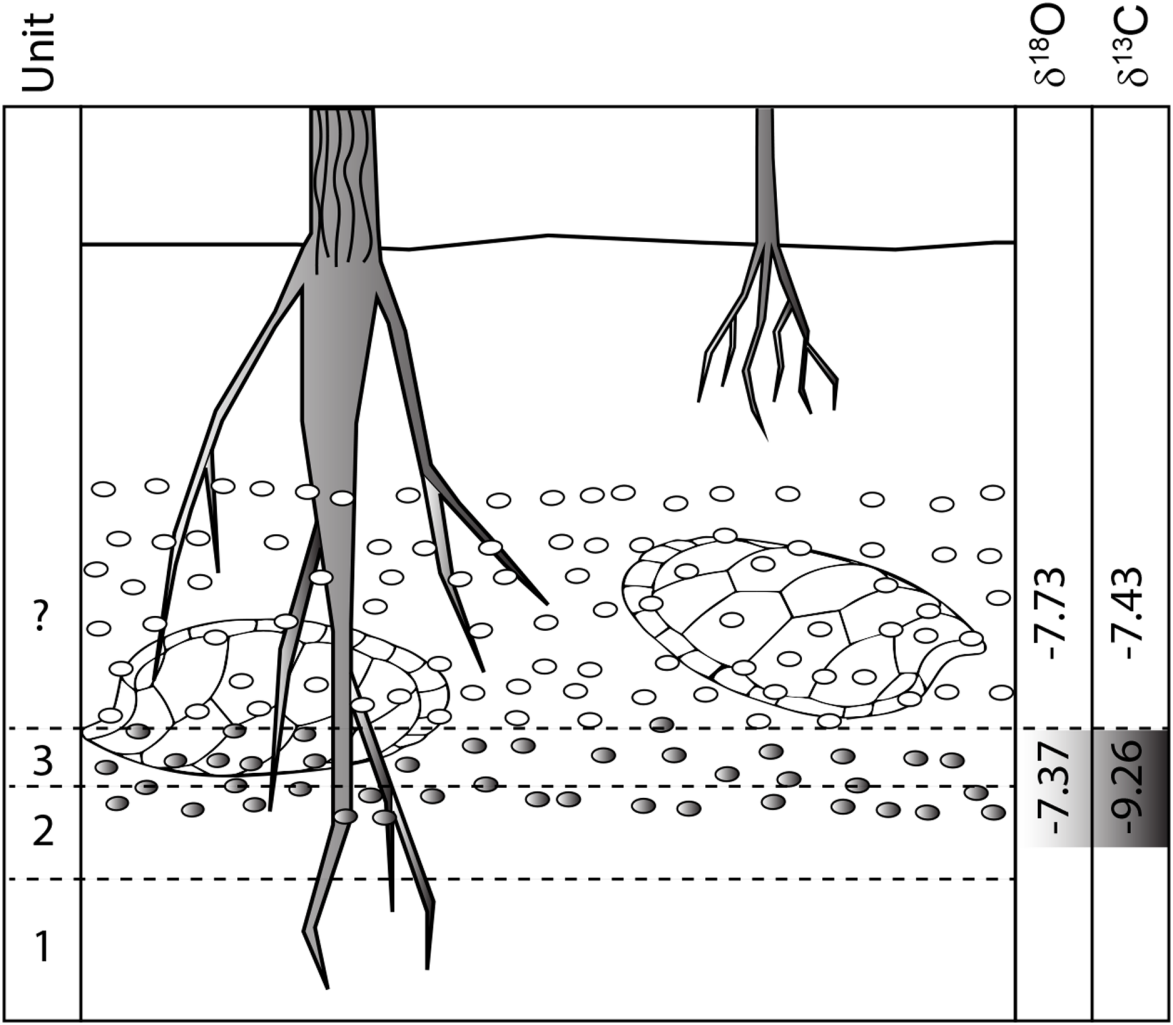
Pre-erosional soil profile showing hypothetical δ^18^O and δ^13^C isotopic patterns for unit 3 and overlying strata (now removed). δ^18^O values stay relatively constant because they are generated by similar water regimes while δ^13^C values increase towards the surface as atmospheric input into soil increases. Isotopic values are from Table 1.

**Discussion:** The independent analysis of spar and micrite components in unit 3 nodules shows that they formed under different conditions, almost certainly at different times. The tight cluster of micrite values for unit 3 nodules, which we interpret as the primary Cretaceous age pedogenic calcite, indicates they all formed from waters with nearly identical composition. Much more widely scattered results from the sparry fraction suggest remobilization of the micritic calcite and random mixing with a slightly heavier fraction during later diagenesis or possibly even Holocene weathering. The oxygen isotopic values from unit 4 nodules, which do not contain sparry fill, are nearly identical to those of unit 3 micrite, indicating they are all from the same pedogenic process and meteoric water sources. However, the lighter C-isotopic values of the micrite component of unit 3 nodules argue they formed lower in the soil profile with a greater proportion of plant root respired CO_2_ and restricted input from atmospheric CO_2_ than unit 4; a finding consistent with our interpretation that most lag material in the unit 4 bonebed was locally derived from a slightly higher stratigraphic horizon than unit 3 (Fig. 12).

Turtle skeletal and carapace (phosphate) oxygen isotope composition (δ^18^O_p_) directly reflects their aqueous (δ^18^O_w_) habitat (e.g., small pond vs. large stream) (Barrick, Fischer & Showers, 1999; Coulson et al., 2008). Given the calculated water isotope values from the RUQ turtles (Fig. 11B), excepting the giant panchelonioid, it is likely they all inhabited water with a similar isotopic composition during phosphate precipitation and therefore represent individuals derived from the same spatial/environmental/sedimentary context. The giant panchelonioid is statistically different; being approximately 1.6‰ more enriched in ^18^O (calculated δ^18^O_w_ average-9.4‰) relative to the other turtles, suggesting it lived in isotopically enriched ponded water, spent more time out of water subjecting itself to evaporative conditions, or spent time closer to the coast. Alternatively, the giant panchelonioid could have had a unique physiology that enriched its body water relative to smaller (or younger) counterparts, a phenomenon observed in other giant taxa (e.g., sauropods and very large theropods) (Suarez et al., 2014). There is no evidence to suggest it routinely inhabited marine environments as it would present much higher δ^18^O_p_ and calculated δ^18^O_w_ (Cretaceous marine water = 0 to −1.2‰) (Suarez, González & Ludvigson, 2011; Ufnar et al., 2002). Simple mass balance modeling suggests that if the giant panchelonioid did spend time in marine water, it would only have been for ~10% of its entire growth history.

### Rare earth elements

**Methods:** Approximately 0.1 g samples were taken from tyrannosaur dentine and one vertebra, fish scales and one vertebra, dentine from *Deinosuchus*, four turtle shells, a carbonate nodule from unit 4, and a carbonate nodule embedded within a tyrannosaur vertebra. These were crushed or drilled, dissolved in 0.28 mL of 15.7 M trace grade nitric acid and diluted to 10 mL using DDI water, then analyzed for cerium through lutetium (lanthanum was not analyzed) on a Thermo iCap q inductively-coupled mass spectrometer at the Trace Element and Radiogenic Isotope Lab at the University of Arkansas. All samples were then normalized to the North American Shale Constant (NASC) using data in Gromet et al. (1984).

**Results**: Data is summarized in Table S3. Total rare earth element (REE) abundance ranged between 78.2 and 4,662.1 ppm for bone/dentine. The two carbonate nodules analyzed (one from within a bone cavity and one from unit 4) ranged between 33.6 and 214.4 ppm. NASC-normalized data show values for all taxa and the carbonate nodules are light REE (LREE) and middle REE (MREE)-enriched and heavy REE (HREE)-depleted. Fish and tyrannosaur material have NASC-normalized patterns that are indistinguishable from each other. Three of the four turtle elements have similar REE patterns to the tyrannosaur and fish material but are lower in overall REE concentration. Finally, the carbonate nodules’ REE patterns are also HREE-depleted.

**Figure 13:**
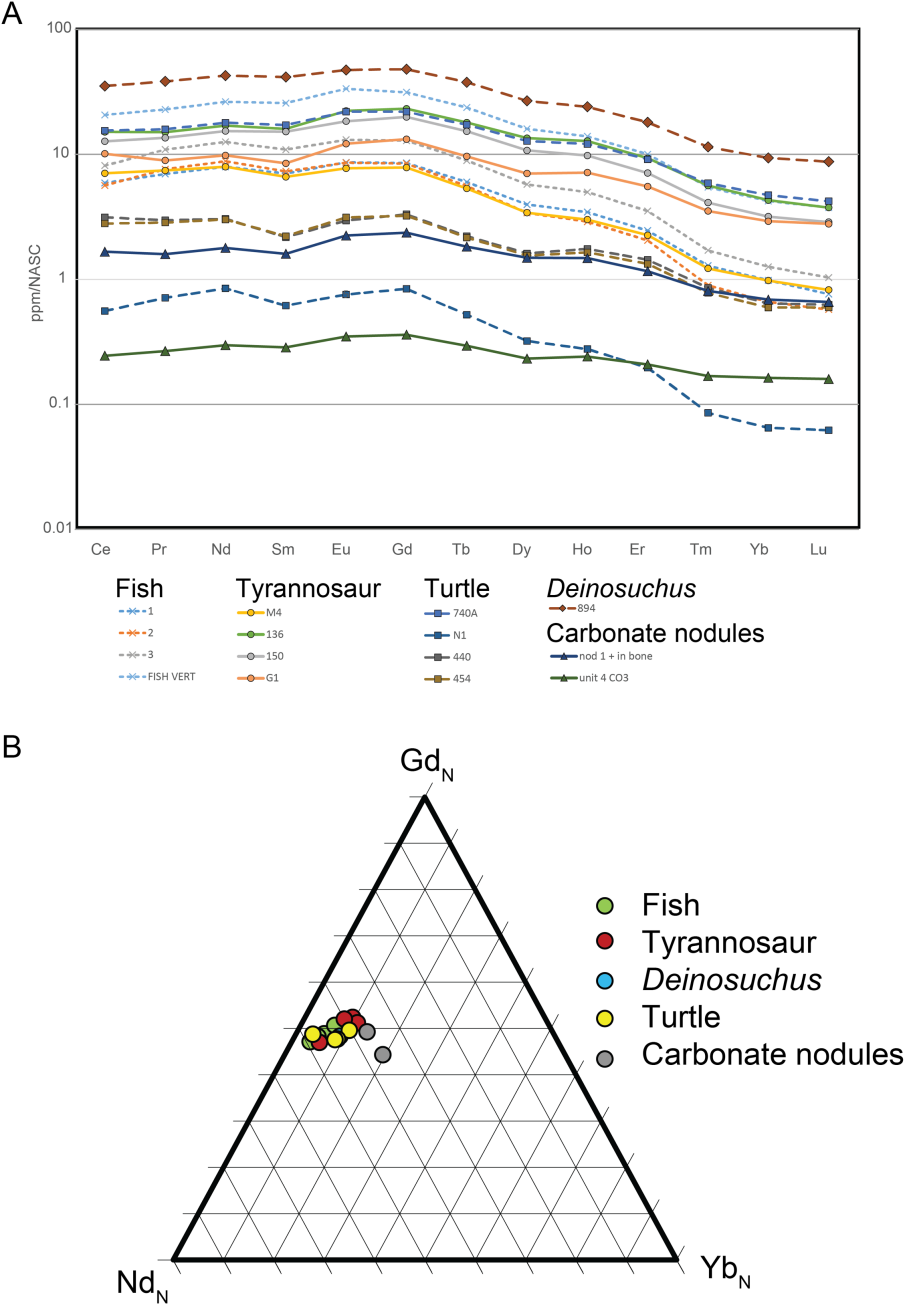
NASC-normalized REE patterns and ratios for RUQ fossils and carbonate. (A) REE patterns that show a HREE-depleted pattern for all bones, dentine and carbonate nodules. (B) Ternary diagram of representative normalized light (Nd_N_), middle (Gd_N_) and heavy (Yb_N_) REEs. The closely similar REE patterns and tight clustering demonstrate similar early fossilization histories for all sampled specimens.

**Discussion**: Rare earth elements (La 139 to Lu-175) are incorporated in fossil bones and teeth very early in their diagenetic history (Trueman & Tuross, 2002; Kohn & Moses, 2013; Suarez & Kohn, 2020) via a diffusion reaction process as fossilizing fluids (typically groundwater) trigger apatite recrystallization and replacement of (mostly) calcium cations in the crystal lattice (Millard & Hedges, 1996; Suarez & Kohn, 2020). The REE pattern, that is the relative proportions and abundance of LREEs, MREEs and HREEs are controlled by Redox, pH, dissolved colloids and ligands, and source rock and reflect the early diagenetic history of the geographic setting (Trueman, 1999). Thus, bones sharing the same fossilization history have closely similar REE patterns even though their absolute abundances differ. The similarity of REE patterns and ratios for all taxa analyzed (Fig. 13) demonstrate they share the same fossilization history, while similarities of the bone REE pattern to the carbonate nodule REE pattern (Fig. 13) indicate the overall signature was imparted during pedogenic carbonate formation (Fig. 12); i.e., during pedogenesis (Grandstaff & Terry, 2009; Metzger, Terry & Grandstaff, 2004; Suarez et al., 2007). This is corroborated by the HREE-depleted signature of RUQ fossils, which is typical of high suspension load or colloid-rich fluvial environments like floodplains (Herwartz et al., 2013; Rousseau et al., 2015), indicating REE infusion of fossils and peds was essentially complete prior to incorporation into the channel setting of units 4 and 5, which would have enriched the bones with HREEs (Trueman, 1999). The overall lower abundance of REE in the bones also suggests very little diagenetic alteration and rapid fossilization (Ullmann et al., 2020).

## Charcoal analysis

**Methods:** Macroscopic organic particles resembling charcoal were identified and collected from units 4 and 5 at thirteen grid locations within the mapped area of tyrannosaur bone concentration (Fig. 7). The samples were treated by standard hydrofluoric acid maceration to liberate the organics (see Pearson & Scott, 1999). The largest fragments were selected from each macerate for analysis using incident light microscopy. In addition to these twenty fragments (Table 4) a random strew of particles from sample 17C-3 was embedded for reflectance microscopy alone, allowing reflectance to be quantified independent of sampling bias. Upon polishing, an additional ten clasts were intersected for analysis, bringing the total number of specimens for reflectance analysis to 30.

**Table 4:** Summary of unit 4 and 5 charcoal specimens (sample locations shown in Fig. 7). Given are their approximate volume (mm^3^); their orientation (TLS = tangential longitudinal section, RLS = radial longitudinal section, TS = transverse section) as seen in reflected light microscopy (RL); evidence of brittle fracture (one character used to identify charcoal); cell wall homogenization (absent = a middle lamella is not discernible by either SEM or RL; this is a characteristic of charcoal); a characterization of the tracheid pitting and the ray parenchyma, in particular cross field pitting as an aid to taxonomic identification; other features with potential bearing on the specimens taphonomy.

Macerate #	Sample #	Spec. Vol. (mm^3^)	Orientation in RL	Brittle Fracture	Middle Lamella	Pitting	Rays	Possible alteration
15C	15C-1A	360	TLS	Present	RL only	Taxodioid-bordered	Abundant, 6-21 cells high, uni-biseriate, opposite; uniseriate bordered pits large (degraded)	Micro-checking in walls, degraded rays, infilling of some lumina, ray cell walls separating
	15C-1B	110.25	RLS-TS	Present	RL only	Bordered	Abundant, 8-17 cells high, uniseriate (degraded), transverse walls nodular	Infilling of some lumina, ray cell walls separating & degrading
15F-A	15F-AA	3.75	TLS	Unknown	Unknown	Not observed	Not observed	Not observed
	15F-AB	1.5	TS	Present	Absent	Not observed	Not observed	Not observed
	15F-AC	0.25	TLS	Present	Absent	Not observed	Not observed	Not observed
15F-B	15F-B	1	TLS	Present	Absent	Not observed	7 cells high, uniseriate	Not observed
15F-C	–	–	–	–	–	–	–	–
16A-A	16A-AA	10.5	TLS-TS	Present	Absent	Bordered, uni-biseriate (opposite)	Uniseriate	Micro-checking through pits
	16A-AB	9	TLS-TS	Present	Absent	Not observed	Not observed	Not observed
16A-B	16A-BA	2	TLS	Absent	Unknown	Not observed	Not observed	Not observed
	16A-BB	1.5	TLS-TS	Absent	Unknown	Not observed	Not observed	Not observed
	16A-BC	1.5	TLS-TS	Absent	Unknown	Not observed	Not observed	Not observed
17C-1	17C-1A	224	RLS	Present	RL only	Taxodioid-bordered	Abundant, high, uniseriate-sub-opposite borderd pits large, transverse & end walls nodular	Not observed
	17C-1B	539	TS	Present	Absent	Not observed	Abundant (degraded)	Not observed
17C-2	17C-2	108	TLS	Present	Absent	Not observed	9 cells high, uniseriate	Not observed
17C-3	17C-3A	12.5	TLS	Present	RL only	Bordered	4-12 cells high, uni-biseriate, opposite, borderd pits	Not observed
	17C-3B	9	TLS	Present	RL only	Not observed	9 cells high, uni-biseriate, opposite	Infilling of some lumina, rare ray cell walls separating
	17C-3-Strew1	N/A	TS	Present	Absent	Bordered	Uniseriate	Not observed
	17C-3-Strew2	N/A	TS	Present	Absent	Not observed	Uniseriate	Not observed
	17C-3-Strew3	N/A	TS	Present	Absent	Not observed	Not observed	Not observed
	17C-3-Strew4	N/A	TLS	Present	RL only	Not observed	Not observed	Unidentified tissue with irregularly thickened & deformed cell walls, cells often infilled
	17C-3-Strew5	N/A	TS	Present	RL only	Not observed	Not observed	Not observed
	17C-3-Strew6	N/A	TLS?	Present	Absent	Not observed	Not observed	Unidentified tissue with irregularly thickened & deformed cell walls, cells often infilled
	17C-3-Strew7	N/A	TS?	Present	RL only	Not observed	Not observed	Unidentified tissue with irregularly thickened & deformed cell walls, cells often infilled
	17C-3-Strew8	N/A	TS	Present	RL only	Not observed	Not observed	Not observed
	17C-3-Strew9	N/A	TLS	Present	Absent	Not observed	Not observed	Not observed
	17C-3-Strew10	N/A	TLS?	Present	Absent	Bordered	Uniseriate	Not observed
17C-4	17C-4A	78	TS	Present	Absent	Not observed	Not observed	Not observed
332A	332A-A	315	TLS	Present	Absent	Not observed	Not observed	Not observed
774A	774A-A	216	TS	Present	Absent	Uniseriate, bordered	Short, uniseriate (degraded)	Not observed
X	X-A	212	LS	Present	Absent	Bordered	Abundant, short, uni-biseriate, opposite (degraded)	Not observed

While picking, an assessment of volume, luster and color was made. Specimens were mounted for scanning electron microscopy on adhesive Pelco tabs and sputter coated with gold for 90 s prior to scanning and imaging with a Hitachi S2700 scanning electron microscope.

Following SEM, the specimens were removed from their tabs and embedded in Epofix low-viscosity resin, following manufacturer protocols, prior to being hand polished using a series of coarse-to-fine grit papers and a sequentially finer series of aluminum oxide slurries on woven and napped cloths; with the final polish at 0.05 microns.

Polished blocks were imaged under oil (refractive index 1.5180 at 23 °C) in plane-polarized light at 546 nm wavelength using a Leica DMR microscope fitted with a 50× oil immersion lens. Following a preliminary assessment of estimated reflectance, the system was calibrated for each specimen using four optical standards closest in range to this estimate (using methodology in Taylor et al. (1998)), in each case these were Spinel, Leuko-Sapphire, YAG and GGG spanning a range of reflectance from 0.425% to 1.705%. Digital images were captured for greyscale analysis and where large enough, specimens were measured at 100 different locations, ten pixels being measured and averaged from each location. Smaller fragments were imaged at as many points as possible where reliable data could be gathered. Several smaller fragments (approximately 0.25–4 mm^3^), plus some of those embedded in the strew, yielded very low numbers of reliable data points and are considered less statistically robust.

Both mean reflectance (Ro_MEAN_) and highest observed reflectance (Ro_HO_) were calculated. Ro_HO_ does not equate to Ro_MAX_ or apparent Ro_MAX_ but is simply the highest random reflectance value measured, which can then be used to estimate the minimum fire temperature (Scott & Glasspool, 2005). Minimum fire temperature was calculated from experimental charring data published in Scott & Glasspool (2005) using a combination of 1 hr and 24 h data sets to generate a polynomial regression line (*R*^2^ = 0.9802).

**Figure 14:**
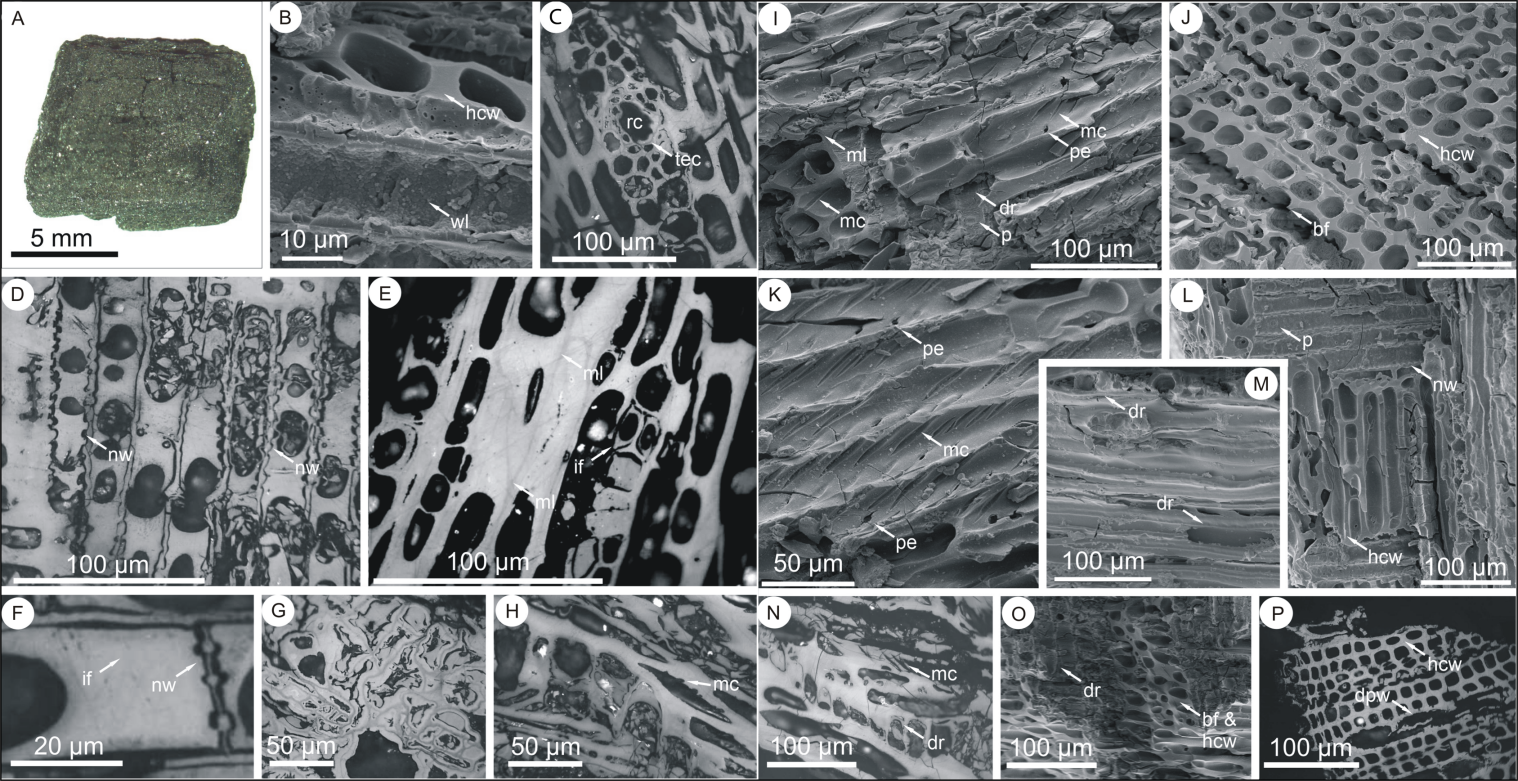
Standard light and SEM images of charcoal specimens from units 4 and 5. (A) 17C-1B. (B, D, F and L) 17C-1A. (C) 17C-3B. (E, I and K) 15C-1A. (G) 17C-3 Strew 7. (H and N) 15C-1B. (J and M) X-A. (O) 774A-A. (P) 17C-3 Strew 1. **Image mode**: (A) incident light; (B, I–M and O) scanning electron microscopy; (C–H, N and P) reflected light. **Abbreviations**: bf = brittle fracture, dpw = degraded parenchyma wall, dr = degraded ray cells, hcw = homogenized cell wall, if = infilled cell, mc = microchecking, ml = middle lamella, nw = nodular parenchyma wall, p = pitting, pe = pitting elongation, rc = resin canal, tec = thick celled epithelial cells, wl = warty layer. **Description: A**: a cubic (though compressed in the z-dimension) woody fragment of charcoal and showing characteristic black coloration and silky luster. **B**: cell wall homogenization and a possible warty layer. **C**: conifer resin canal bordered by thick walled epithelial cells. **D**: ray parenchyma exhibiting nodular transverse walls. **E**: cell walls superficially appeared homogenized when viewed by SEM (see: fig. I) though preserving localized evidence of a middle lamella. Here, the lignin-rich middle lamella is clearly visible as a less reflective (darker grey) layer (arrows) surrounded by higher reflecting cellulosic layers. **F**: ray parenchyma exhibiting nodular end walls. **G**: unidentified chaotic tissue with differential reflectance, cell infillings and variably thickened and distorted cell walls. **H**: degraded parenchymal tissues (bottom left) and a tracheid in which microchecks are discernible in cross-section penetrating into the tracheid wall. **I**: tracheids in which microchecks are visible both through the cellulose wall and as elongation of the pitting. The specimen also preserves a bordered pit within degraded ray parenchyma. The tracheid end walls exhibit brittle fracture and appear homogenized except at the arrow where evidence of the middle lamella is discernible. **J**: cell wall homogenization and brittle fracture. **K**: tracheids in which microchecks are visible both through the cellulose wall and as elongations of the pitting. **L**: tracheids with homogenized cell walls and a bordered pit within a parenchyma ray, the transverse walls of the ray cells are nodular. **M**: degraded ray cells in longitudinal section. **N**: degraded, distorted parenchymal tissues in which the cell walls show evidence of separation (dr) and a tracheid in which microchecks are discernible penetrating into the tracheid wall. **O**: brittle fracture and homogenized cell walls in comparably well preserved tracheids and highly degraded ray cells. **P**: woody tissues in which the tracheids exhibit brittle fracture and are well-preserved, with clean, entire cell margins (hcw) conversely ray cells have collapsed and show evidence of cell wall degradation (dpw).

**Results:** Specimens analyzed were small, ranging in volume from ~540 mm^3^ to just 0.25 mm^3^ (See: Table 4). Larger fragments were typically cubic (Fig. 14A), though somewhat rounded in shape and flattened in one dimension. All specimens were black and exhibited a silky luster in incident light. In many, elongate cellular elements comparable to the vascular tissues of wood were apparent.

Viewed by SEM, all specimens proved to be woody tissues though the degree of preservation was highly variable. All identifiable specimens were coniferous and no conclusive vessel elements were recognized.

Traces of a middle lamella were discernible in nine specimens (e.g., Figs. 14E and 14I), while 17 had homogenized cell walls (Figs. 14B, 14J, 14L, 14O and 14P; see also Table 4). However, brittle fracture was observed in all but four specimens (Figs. 14J and 14O; see also Table 4). All pitting observed was uni- or biseriate bordered or taxodioid (Figs. 14I, 14K and 14L; see also Table 4), with some specimens preserving both types. A possible warty layer (Fig. 14B) was observed lining the lumina of tracheids in two specimens. A normal horizontal resin canal (Fig. 14C) surrounded by thick walled epithelial cells was observed in one specimen (17C-3B). Ray cells were degraded in most specimens (Fig. 14M, 2G; see also Table 4) and cross field pitting organization was distinct in only one specimen 17C-1 (Fig. 14L). Other features of note include two specimens in which the ray parenchyma had nodular transverse and end walls (Figs. 14D-nw and 14F-nw), six specimens in which ray cell parenchyma were infilled (Fig. 14E-if), two specimens exhibiting microchecking (Figs. 14H, 14I, 14L and 14N; see also Table 4), gashes in the secondary wall (S2) that follow the cellulose microfibril angle, and in seven specimens evidence of both degradation of cell walls and cell wall detachment (Figs. 14H and 14M–14P).

Across all 30 specimens, 18,080 pixels of data were analyzed (10 points at 1,808 data locations. See: Table S4), yielding a mean random reflectance Ro = 1.32% s.d. = 0.28 and highest observed reflectance of Ro_HO_ = 2.10%. Eliminating all less well sampled specimens (i.e., those measured at <40 locations; in Table S3 marked with an asterisk (*)), reflectance values change minimally: *n* = 1,635 locations, Ro = 1.35% s.d. = 0.27, Ro%_HO_ = 2.10. Ro = 1.32% equates to a minimum charring temperature of 416 °C, while Ro_HO_ = 2.10% equates to 479 °C. Lowest recorded mean random reflectance was of Ro = 0.74% (specimen 16A-BB).

**Discussion:** Based on anatomical characters observed by SEM, incident and reflected light microscopy, all specimens are charcoal. Quantitative reflectance data confirm this. In the vicinity of RUQ, coals from the Straight Cliffs Formation, which underlies the Kaiparowits Formation on the Kaiparowits Plateau, are quantified as of high volatile bituminous (hvb) C rank (Fig. 5 in: Kohler, Quick & Tabet (1998)) this equates to a mean random reflectance Ro < 0.79% (the transition from hbv C-B. See: Ward & Suárez-Ruiz (2008)). Therefore, coalified organic matter from the Kaiparowits Formation should have a rank significantly less than hvb C (Ro < 0.79%). Only three specimens from RUQ (16A-BB and BC and 17C-3A) have reflectance values that approach rank maturation of the underlying Straight Cliffs Formation. However, their morphological appearance under incident light shows they were charred.

Given the small size of the fragments, detailed taxonomic identification is not possible. However, all identifiable fragments are coniferous, with some assignable to Cupressaceae and one to Pinaceae. Presence of abundant ray parenchyma (e.g., 15C-1A) is, within modern conifers, characteristic of Podocarpaceae and Cupressaceae, and not Pinaceae (Phillips, 1941). Bordered pitting observed within ray parenchyma closely resembles that illustrated by Gerards et al. (2007) in charred Cupressaceae. Potential affinities with Cupressaceae are strengthened by observations of nodular transverse walls within ray parenchyma, a feature documented by Phillips (1941) within modern conifers in Cupressaceae and Podocarpaceae. Presence of these thickenings within end walls of parenchyma is restricted in modern conifers to certain species of *Juniperus, Libdocedrus decurrens* and members of the Abietoideae (Phillips, 1941). Presence of a warty layer, a continuous coating of proteinaceous cytoplasmic breakdown products deposited over the trachery elements once they mature (Dickison, 2000), is like that seen in modern junipers (Kocoń, 1984). However, the normal horizontal resin canal observed within 17C-3B indicates affinities with Pinaceae (Phillips, 1941), though the thick-walled epithelial cells differentiate it from the genus *Pinus*, within which they are only thin-walled (Phillips, 1941).

All fragments, except some unidentified tissues in three of the strews (17C-3 Strew 4, 6 and 7: see Fig. 14G) are woody, though their fragmentary nature precludes assignment to an organ (e.g., trunk, branch, twig). Microchecks (checks) in wood (Figs. 14H, 14I, 14K and 14N) are longitudinal openings at anatomical weak points resulting from differential shrinkage caused by factors such as water stress, rapid loss of water from exposed cells in senesced wood, or photodegradation by ultraviolet and visible light (Miniutti, 1967; Kollman & Côté, 1968; Donaldson, 2002; Evans, Urban & Chowdhury, 2008). Where related to aerial exposure, microchecks not associated with pits may indicate more severe or longer degradation (Rowell, 2012). Here checks occur in two specimens (15C-1A and 16A-AA) and in one are associated with evidence of extensive ray cell degradation prior to charring. In combination with observations of little tracheid cross-sectional rounding and few intercellular spaces, this degradation indicates senesced wood with microchecking resulting from sub-aerial exposure and its effects.

Evidence of decay includes degradation of ray parenchyma, detectable by SEM (e.g., Figs. 14I, 14M and 14O) and by reflected light microscopy (e.g., Figs. 14N and 14P) where integrity of tracheid walls compared with those of pitted and deformed parenchyma is easily discerned. The nature of the unidentified tissues (e.g., Fig. 14G) is unclear, though they too may indicate severe decay.

Mean random reflectance values for all charcoals are low, none exceeding 2.10% and, across all 30 specimens measured, only encompasses a range of minimum calculated fire temperature ranging from ~350–480 °C. Reflectance of charcoal produced by a range of modern surface and crown fires burning in differing ecological settings and at different intensities has been assessed by Roos & Scott (2018). Their data shows the RUQ charcoal to be atypical in its comparatively high mean random reflectance (Ro = 1.32% s.d. = 0.28), while its very tight range is highly unusual. It is possible that the RUQ charcoal has been taphonomically filtered which can occur both during transport by overland flow and while in a standing or flowing body of water (Scott et al., 2000). There is also a difference in the hydrodynamic properties of fresh wood charcoal dependent upon its temperature of formation (Scott, 2009) that results in taphonomic filtering and bias and it is also possible this latter process governed charcoal deposition at RUQ.

## Taphonomic analysis

**Overview:** A total of 1,019 individually mapped and numbered skeletal elements (Fig. 7) were collected over the course of five field seasons (2014–2018) from an area of approximately 72 m^2^. Numerous other pieces of turtle shell that had been virtually disintegrated by modern weathering as well as groupings of fish scales were mapped but not collected. Many additional elements not seen in the field are still in unprepared field jackets, bringing the estimated total count to 1,300 elements collected. Of these 1,300 elements, the Number of Identified Specimens (NISP) is 391 (Tables S5 and S6), which represents a good cross-section of the tetrapod assemblage collected so far. Both field collections and lab preparation have been biased towards tetrapods, so fish, which are ubiquitous throughout the quarry as disarticulated remains, are vastly under-represented in this report and would dominate the NISP count (mostly scales) if tallied. Tyrannosaurid specimens (Table S5) account for 223 of the total NISP (57.0%); hadrosaurs (18–4.6%), and paravians (8–2.0%) are relatively minor components of the dinosaur assemblage. Following Eberth, Shannon & Noland (2007) and excluding fish from our NISP, the RUQ would currently be classified as a tyrannosaurid (mono) dominant, high diversity mixed macrovertebrate/microvertebrate bonebed, although we believe this assignment will change as the quarry expands into areas with lower densities of tyrannosaurid material.

For tyrannosaurids, out of 223 NISP, 146 of those are classified as complete and can be translated directly into a Minimum Number of Elements (MNE) from which to base estimates of the Minimum Number of Individuals (MNI). The remainder of NISP (*n* = 77), mostly shafts of ribs and gastralia, are fragmentary and difficult to extrapolate into complete elements or assign to right and left. To determine a total figure, we divided fragmentary elements (77) by the arbitrary number of five (based on estimates of how many pieces ribs and gastralia had repeatedly broken into) to approximate a MNE (estimated *n* = 15), bringing the total MNE to 161. To determine the MNI we tabulated right and left counts from MNE data subdivided by our estimates of represented growth stages (very small juvenile, juvenile, large juvenile/subadult, somatic adult). Phalanx counts, particularly of element IV-3, demonstrate a MNI of four in the following size ranges: two moderate size juveniles; one subadult, and a presumed adult. However, a single small right pedal digit III-3 (Fig. 9F-element 728), a tibia-fibula pair, and a single pubic shaft demonstrates there is a very small non-paravian theropod size class also represented. The general morphology of all these elements is distinctly basal coelurosaur with the pubic shaft clearly anteriorly directed and the toe bearing a subrectangular anterior cross-sectional profile with incipient septum on the cotyles and moderately well-developed sulcus between the condyles (ginglymoid). We cannot positively refer it to Tyrannosauridae or Ornithomimidae. However, as no distinctively ornithomimid skeletal elements have yet been identified at the RUQ, these specimens likely indicate the presence of a fifth tyrannosaurid in the small juvenile size class (20% of largest specimen).

**Figure 15:**
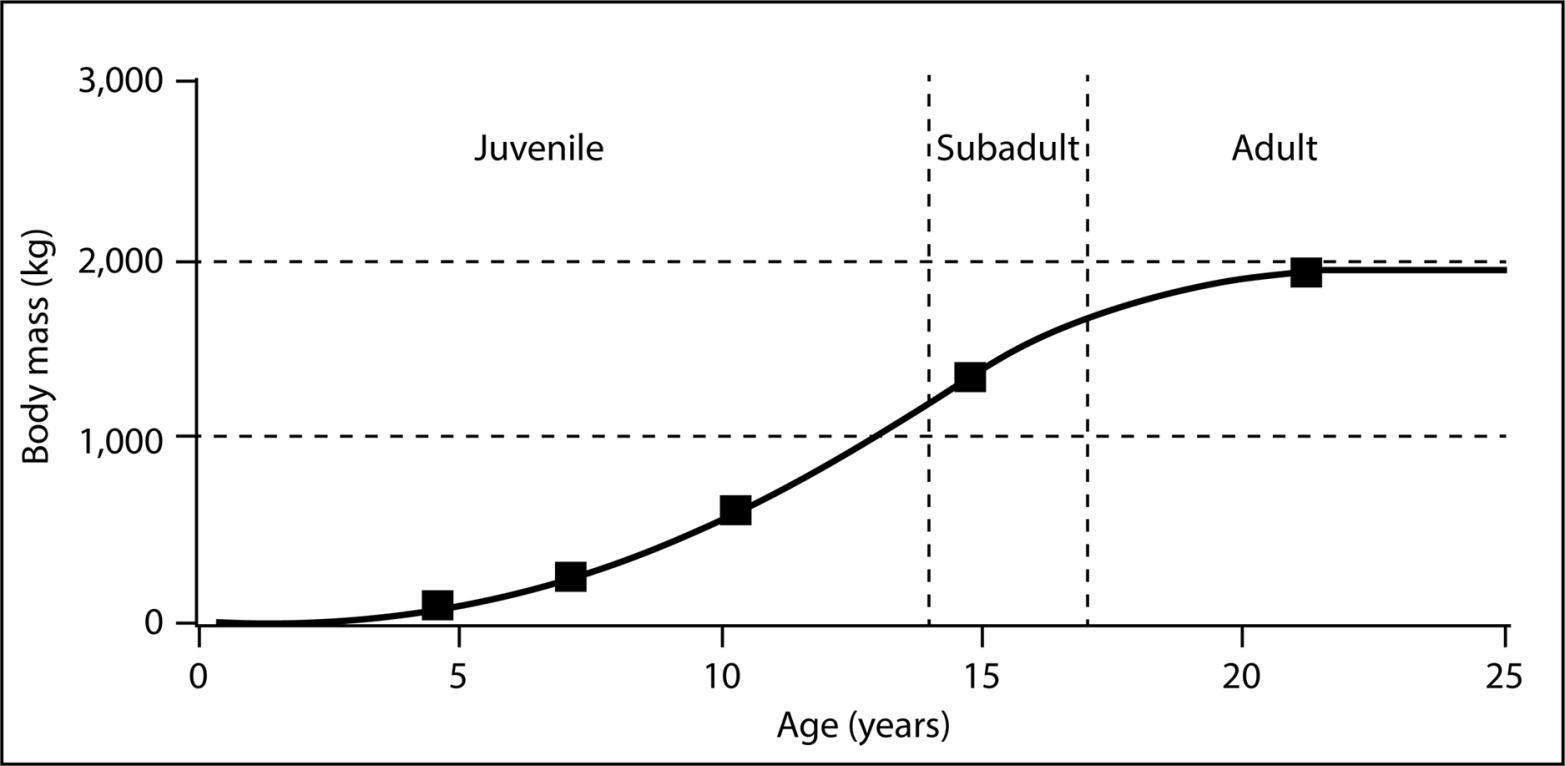
Hypothetical growth curve for cf. *Teratophoneus* with calculated sizes of the four confirmed and fifth possible specimen plotted to show relative developmental stages. Curve is slightly modified from that of *Daspletosaurus* given in Erickson et al. (2004). Age-based growth stages based on Carr’s (2020) estimates for *Tyrannosaurus rex*.

To confirm MNI based on pedal elements we performed a simple dimensional analysis on skull elements and compared the results to the same elements on a reconstructed *Lythronax* skull cast by Gaston Designs (Table 5). Four different size classes of animals are readily apparent: from smallest-to-largest these are 50%, 80%, 100% and 140% the size of the *Lythronax* standard. The largest individual from the RUQ (Fig. 10) features exaggerated craniofacial rugosity, high relief cornual processes and other ornament on the lacrimals, nasals, and postorbitals, all features regarded as indicating somatic maturity verging on senescence in *Tyrannosaurus rex* (Molnar, 1990; Carr, 2020). Assuming this individual is a somatic adult (which is speculation even though it is the largest diagnostic tyrannosaurid cranial specimen collected from the Kaiparowits Formation), then the three smaller individuals represent roughly 40%, 55%, and 70% of terminal adult size. The fifth individual, whose referral to Tyrannosauridae is not certain, is approximately 20% the size of the largest. Length estimates were calculated using comparisons with snout-to-tail tip length of the only known articulated *Teratophoneous* (UMNH VP 21100) as a standard. UMNH VP 21100 measures 7.6 m long from tip of snout to tip of tail, which would make the largest individual at the RUQ approximately 8.7 m long. Based on this estimate, the other individuals at the RUQ would measure 1.7, 3.5, 4.8 and 6.1 m. The only intact femur from the site (element 402) represents an individual in the 40% size class and measures 560 mm long. Using Fig. 12 of Eberth & Currie (2010) as a rough approximation, this individual would have been six years old if they shared similar growth histories. From this we can generalize the four individuals correspond to two juveniles, a subadult, a full adult, with a possible small juvenile (Fig. 15).

**Table 5:** Comparisons of measurable tyrannosaurid cranial elements to a reconstruction of the holotype skull of *Lythronax argestes* (UMNH VP 20200).

Element #	Identification	Size vs. *Lythronax* type (%)	Size vs. Somatic Adult (%)
150-N	L dentary	73	52
74	R postorbital	53	38
194	R lacrimal	78	55
G-15-1	R quadrate	80	57
395	R-L nasals	140	100
300	R dentary	130	100
761	L frontal	100	73
122	R maxilla (height)	88	NA
122	R maxilla (length)	78	56
721	L maxilla (height)	88	NA
721	L maxilla (length)*	78	56

**Notes:**

* Indicates value estimated from incomplete element.

Four basic size classes are represented. Scaled to the largest individual, these represent a somatic adult and 40%, 55% and 70% adult size.

The second highest NISP are from turtles (*n* = 102), of which, *Neurankylus utahensis* (*n* = 68) accounts for 17.3% of the total NISP and 66.6% of the total NISP for turtles. Five specimens of *N. utahensis* are relatively complete shells and can be directly translated into the MNI. We roughly estimate the remainder of carapace and plastron fragments represent another 2–4 individuals. Based solely on the presence of two separate dentaries, the large panchelonioid’s MNI is two. All other turtle taxa have estimated MNI of two or less. *Deinosuchus* material is largely unprepared, so the NISP count for that taxon (*n* = 23), which we are certain represents a single individual, is only an estimated 20% of total elements collected for that specimen. Both hadrosaur and paravian elements appear to represent single individuals, although the hadrosaur is much more widely dispersed throughout the site.

**Figure 16:**
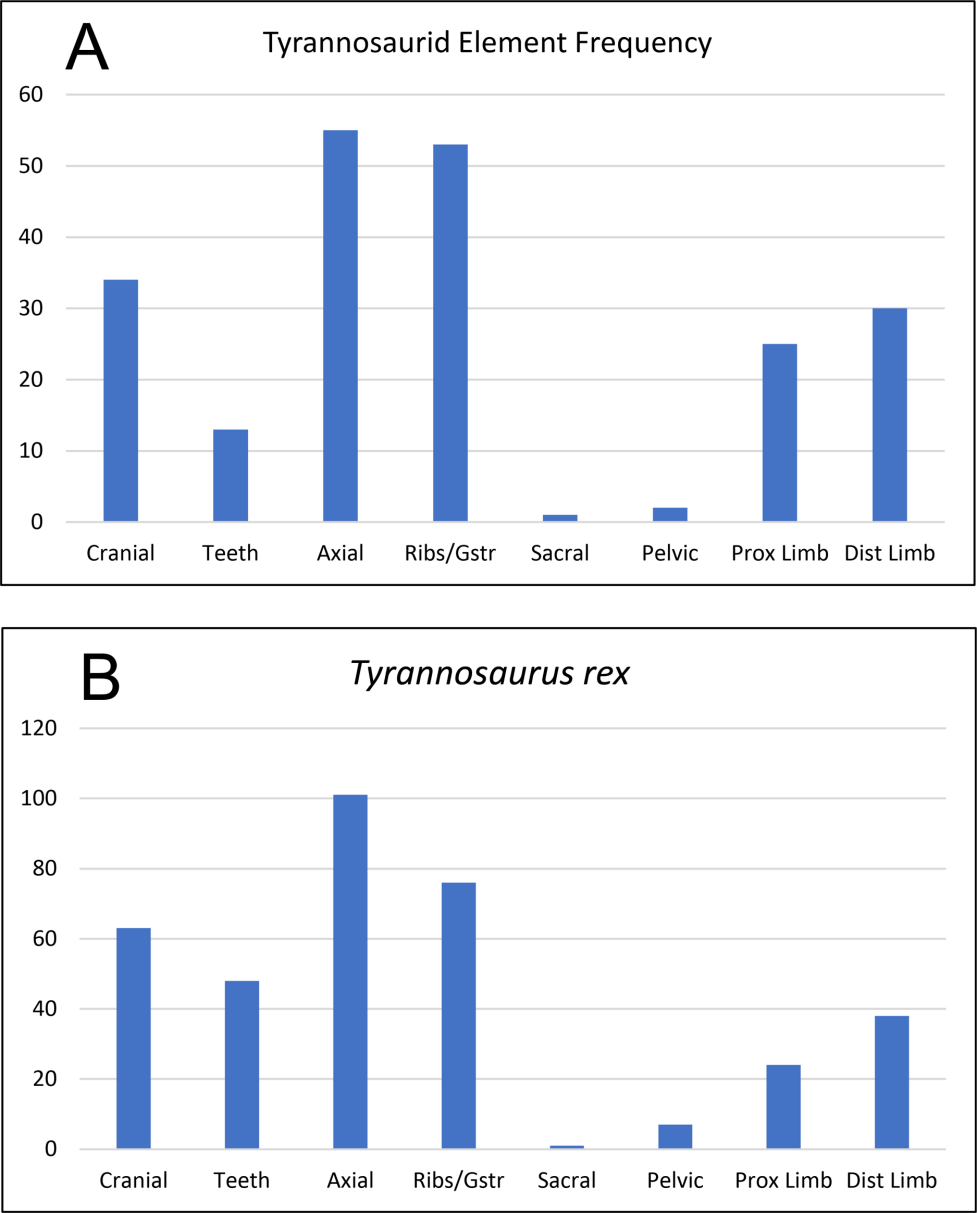
Frequency diagram of all identified elements of cf. *Teratophoneus* from the RUQ site (A), compared to one for a complete skeleton of *Tyrannosaurus rex* (B). The correlation coefficient between the two curves is 0.91, indicating strong positive correlation between the two data sets and suggesting the RUQ tyrannosaurid sample is derived from still largely complete specimens.

**Spatial-Stratigraphic-Size Distribution**: The distribution of elements at RUQ is both highly localized (Fig. 7) and grouped taxonomically (e.g., tyrannosaurid material is largely confined to the northwest area of the site), although exceptions to the latter occur, particularly with smaller taxa. Since the Voorhies indices (Fig. 8) and lack of abrasion indicate low velocity currents and the element frequency counts for the tyrannosaurids (Fig. 16) indicate minimal transport of relatively complete individuals, we suggest taxonomic groupings at the site reflect their approximate original positions before exhumation. This effect could be expected to diminish southward, into the main body of the channel deposit where there were stronger currents.

The highest concentrations of fish and other small material generally occur in the carbonate and clay rip-up facies of unit 4, indicating some degree of hydraulic sorting and lag effects. Larger elements occur as concentrations in both conglomeratic and sandstone facies of both units 4 and 5 and size distribution appears to be relatively random, with small delicate elements (e.g., gastralia) occurring together with large limb elements of the same individual, again demonstrating minimal transport (Fig. 8).

**Orientation**: There is a strong bi-modal distribution of larger long elements (*n* = 90) in the quarry with the dominant direction averaging 314°. Secondary lineation occurs at approximately 220° (90° counterclockwise), indicating that these orientations are non-random, and most likely caused by current flow, with unequally weighted elements oriented parallel to current direction and equally weighted elements oriented perpendicular to current direction. The linear trends of taxonomic groups (Fig. 7) very closely follow the 314° direction exhibited by individual elements as well as general orientation of scour features eroded into unit 3.

**Articulation/Skeletal Completeness:** Even though close anatomical association of elements from individuals in the quarry is common, articulation is rare. Examples include a partial adult tyrannosaurid skull (Fig. 10), a tibia-fibula-metatarsal complex of a juvenile tyrannosaurid, adult tyrannosaurid metatarsals, and caudal sections with two or three elements from both hadrosaurs and tyrannosaurids. Articulation in fish and other smaller taxa is limited to a couple of examples of integrated ganoid scales of *Lepisosteus* and a string of teleost vertebrae (Fig. 9B). Frequency analysis of tyrannosaurid skeletal element groups from the RUQ (Fig. 16A) presents a very similar pattern to that of the overall curve of the same groupings from a complete *T. rex* skeleton (Fig. 16B). As the two number sets have a correlation coefficient (*r*) of 0.91, the RUQ individuals were largely complete upon initial burial and have not been significantly size-sorted or transported during subsequent exhumation and reburial. The *Deinosuchus* specimen is the most complete specimen recovered so far, with an estimated 70% represented.

### Bone modification, weathering and breakage

Methods.—A large percentage of fossil material recovered in the first 2 years of work at RUQ was within one meter below the original land surface in either regolith or soil and therefore showed evidence of recent (Late Holocene) weathering, the most profound of which was caused by plants. Common shrubs and trees at the RUQ site include Fremont Barberry (*Berberis fremontii*), Roundleaf Buffaloberry (*Shepherdia rotundifolia*), Mormon Tea (*Ephedra viridis*), Pinyon Pine (*Pinus edulis*) and Utah Juniper (*Juniperus osteosperma*), all species with typical xeric deep penetrating, widely spreading root systems (Schenk & Jackson, 2002) that aggressively attack fossil bone after it’s encountered in the subsurface. Root effects on bones include pulverization through digestion, cracking by penetration, and erosion of cortical surfaces leaving characteristic shallow, branching grooves (Schultz, 1997). Such alterations on skeletal material at the RUQ makes taphonomic grading difficult or even impossible where large areas of cortical surface are affected. As a result, all prepared specimens were examined microscopically to differentiate those with original (i.e., Cretaceous) eroded/flaked surfaces from those with Holocene modifications. Specimens with an estimated 50% or more of their surface areas showing modern root damage were not evaluated. Specimens recovered later from more consolidated rock were less ambiguous, showing cortical surfaces mostly undamaged by modern roots. Great care was taken to not add damage to cortical surfaces during the preparation process, but a few specimens were rejected because of “vigorous” preparation.

All prepared bones were also examined for evidence of abrasion or wear and macro or micro feeding traces such as grooves, gnaw marks, scratches, or punctures. No such abrasion or traces were found indicating very little-to-no post-mortem scavenging or transport. Subsequently, taphonomic grade was evaluated on 194 elements of various taxonomic groups (Table S6); Osteichthyes (*n* = 20), Testudines (*n* = 38), Hadrosauridae (*n* = 15), Tyrannosauridae (*n* = 113), Paraves (*n* = 8), to characterize the basic taphonomic signature of the material using Behrensmeyer’s (1991) system of taphonomic indices (TI) ranging from 0 to 5.

Since every element exhibited at least some form of cracking in the cortical surface, no 0 values were recorded. Elements with unblemished cortical surfaces but showing even pervasive cracking were assigned a value of 1. Fragmental specimens showing significant loss of cortex and rounded fracture edges were assigned a value of 4. No values of 5 (highly exfoliated, extensively fragmented) were recorded regardless of if they were collected as fragments.

The breakage pattern analysis was done on a subset of 129 elements using the system of Villa & Mahieu (1991) to assess dominant fracture type as well as the percentage of surface area affected by breakage. Although fractures in bones occur in a wide variety of patterns, two primary patterns have emerged as useful for taphonomic analysis: “wet” (formerly perimortem) and “dry” (substantially post-mortem). The former typically forms spiral (curving) or acute/obtuse fracture patterns on elements that result from the more homogeneous mechanical properties of bone still reinforced with collagen fibrils, while the latter results from less randomly propagating rectangular fracture patterns that reflect pure mineral structures (Villa & Mahieu, 1991; Moraitis & Spiliopoulou, 2006). An intermediate pattern of non-curving diagonal fractures has also been noted. As a result, orthogonal or rectangular breakage patterns (=dry) indicate a substantial amount of time has passed between the death of the organism and breakage (Sauer, 1998; Villa & Mahieu, 1991). Breakage patterns were classified as either oblique, orthogonal, or intermediate.

**Results:**

**Figure 17:**
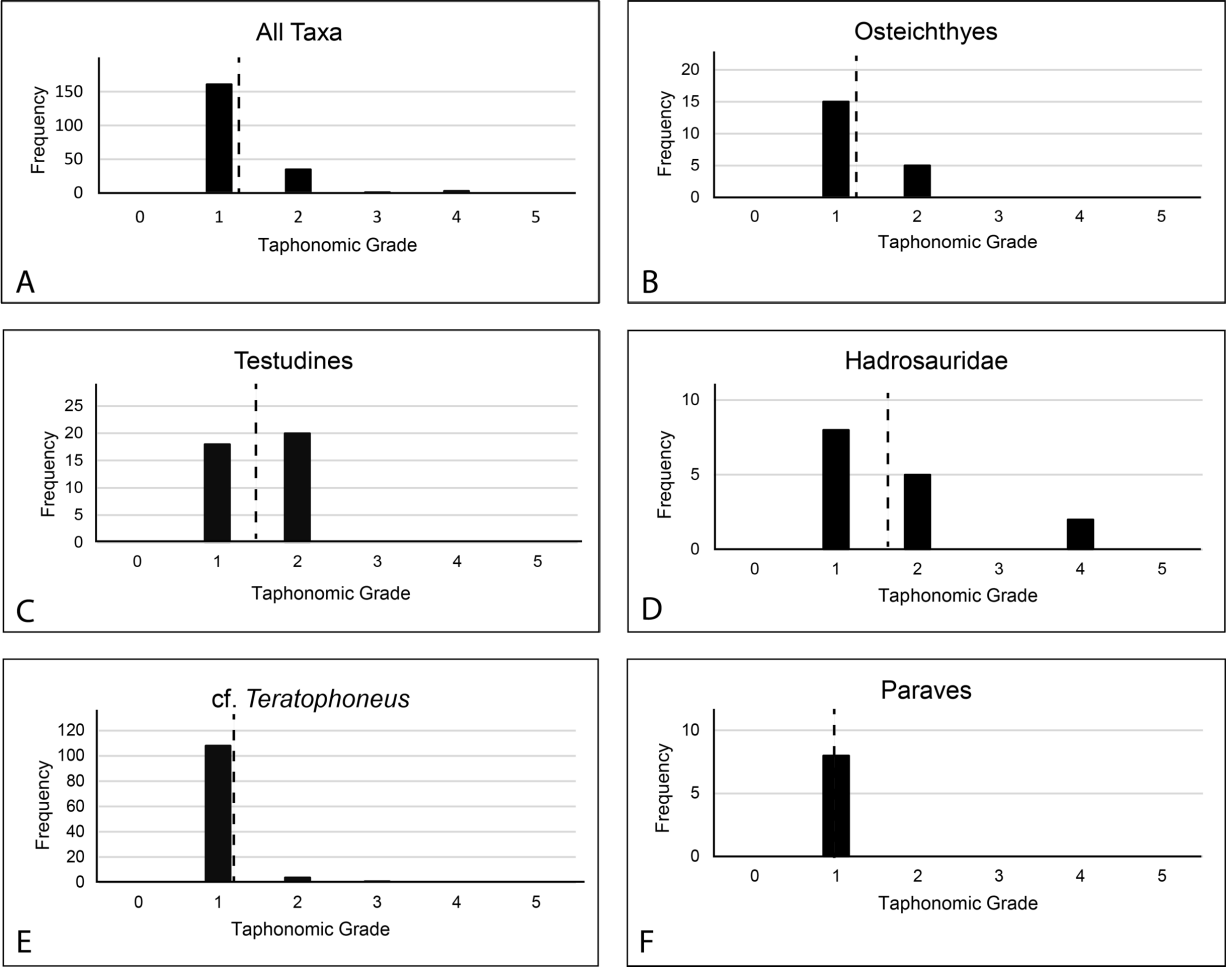
Frequency diagram showing total number and weighted averages (dashed vertical line) of taphonomic grade assignments for both the entire graded assemblage (A) and by major taxonomic groups (B–F). Data from Table S6.

*Taphonomic Grade*.—A total of 200 specimens were graded. Of these, 161 rated as 1; 35 elements rated as 2; 1 element rated as 3; 3 elements rated as 4. This is a total weighted average of 1.23 (Fig. 17). Only 194 were assignable to specific taxa. Of these, the weighted average for osteichthyans (1.25 from 1 to 15, 2 to 5), cf. *Teratophoneus* (1.05 from 1 to 108, 2 to 4, 3 to 1) and paravians (1 from 1 to 8) is lower, while that of turtles (1.52 from 1 to 18, 20 to 2) and hadrosaurids (1.73 from 1 to 8, 2 to 5, 4 to 2) is higher. Three elements rated (2 at 1 and 1 at 4) were not assignable to specific taxonomic groups. Of special note is the juvenile *Deinosuchus* specimen recovered in 2019. Most of this material is still being prepared, but anecdotally it would rank largely as taphonomic grade 1. This specimen is unusual in that it displays a much lower degree of breakage in the bones, is laid out in gross anatomical sequence, and was associated with probable gastroliths, indicating trivial post-mortem transport.

**Figure 18:**
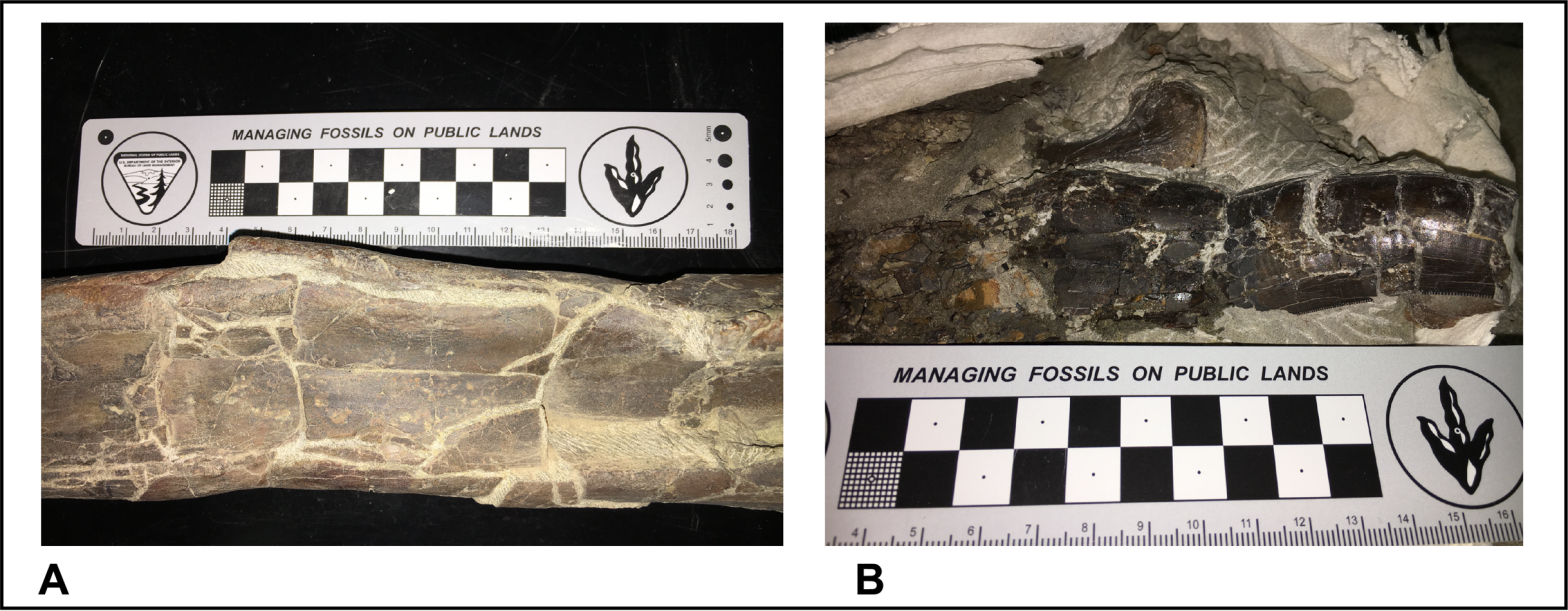
Photos of fractured tyrannosaur elements. Juvenile tyrannosaurid tibia (A) and adult tooth crown (B) from unit 5 showing typical rectangular (orthogonal) fracture patterns observed on nearly all skeletal material from the RUQ.

*Fracture Type*.—123 out of 129 total elements (95%) examined for fractures were broken in some fashion. Even normally robust elements like tibiae and tooth crowns are extensively fractured (Fig. 18). The dominant fracture pattern (Fig. 18A) on every observed element (*n* = 123) for every taxonomic group was orthogonal (dry). Acute or obtuse fractures are uncommon. Percentage of surface areas affected by fractures ranged from 0% to 90% with an average of 33.1%. The lack of more than trivial dilational displacement in many fractures suggests that brittle failure occurred during compaction of units 4 and 5 (Fig. 18A). Pre- or syndepositional breakage is also a common feature, but in many cases fragments of the same broken element are still resting relatively close to each other.

**Discussion:** The element orientations at the RUQ clearly indicate at least some current winnowing, transport, and reorientation. However, since all Voorhies groups are well represented (Fig. 8), transport was minimal. It also appears most material was isolated from surface weathering conditions relatively rapidly after death since the weighted average for the site’s overall taphonomic grade is 1.23. Wet rot is rare but may be present on some of the more susceptible juvenile tyrannosaurid long bones with less developed cortices. Hadrosaurs (1.73) and turtles (1.52) have the highest overall numbers, suggesting at least some individuals had longer residence times at the surface or in aerated soil with high destructive potential. The fish collectively are also slightly elevated (1.25) over the theropods (large and small), which both essentially rank as ones.

The pervasive orthogonal fracturing of elements at RUQ is somewhat paradoxical to their very low taphonomic grades. Normal disintegration under surface conditions starts at grades 4–5 (Behrensmeyer, 1978, 1991), at which point the cortical surface of even larger theropod elements would be showing obvious flaking comparable to that observed in mammal bone (Behrensmeyer, Stayton & Chapman, 2003; Cruz, 2008). Since such cortical erosion is absent in most RUQ material, the fracturing process was not connected to surface weathering. The *Allosaurus* femur illustrated by Gates (2005, fig. 5), broken orthogonally in similar fashion to material from RUQ, was hypothesized to have been trampled. However, the pervasive nature of orthogonal fracturing at RUQ (Fig. 18) causes us to appeal to a more universal process. Pokines et al. (2018) demonstrated experimentally using deer bones that pervasive fracturing of even robust elements can be achieved by simple cyclic wetting and drying independent of any other destructive factors, apparently as a result of volume changes in the cortex. Field observations of flamingo bones undergoing repeated wet-dry cycling and salt crystallization at the surface drove similar conclusions (Prassack, 2011). Therefore, the most likely mechanism for fracture development seems to be subsurface wet-dry cycling (i.e., shrink-swell) that weakened bone already stripped of most of its organic component, resulting in “dry” fracture patterns but leaving the cortical surfaces largely unblemished. If we are correct, this is a not a well documented taphonomic process and further experimentation is warranted. A relatively long subsurface residence time in a cyclic wet-dry environment is evidenced by the pedogenic carbonate infilling of some of the bones (Fig. 6A), which pre-dates mechanical failure/breakage upon burial in units 4–5.

## Synthesis

**Figure 19:**
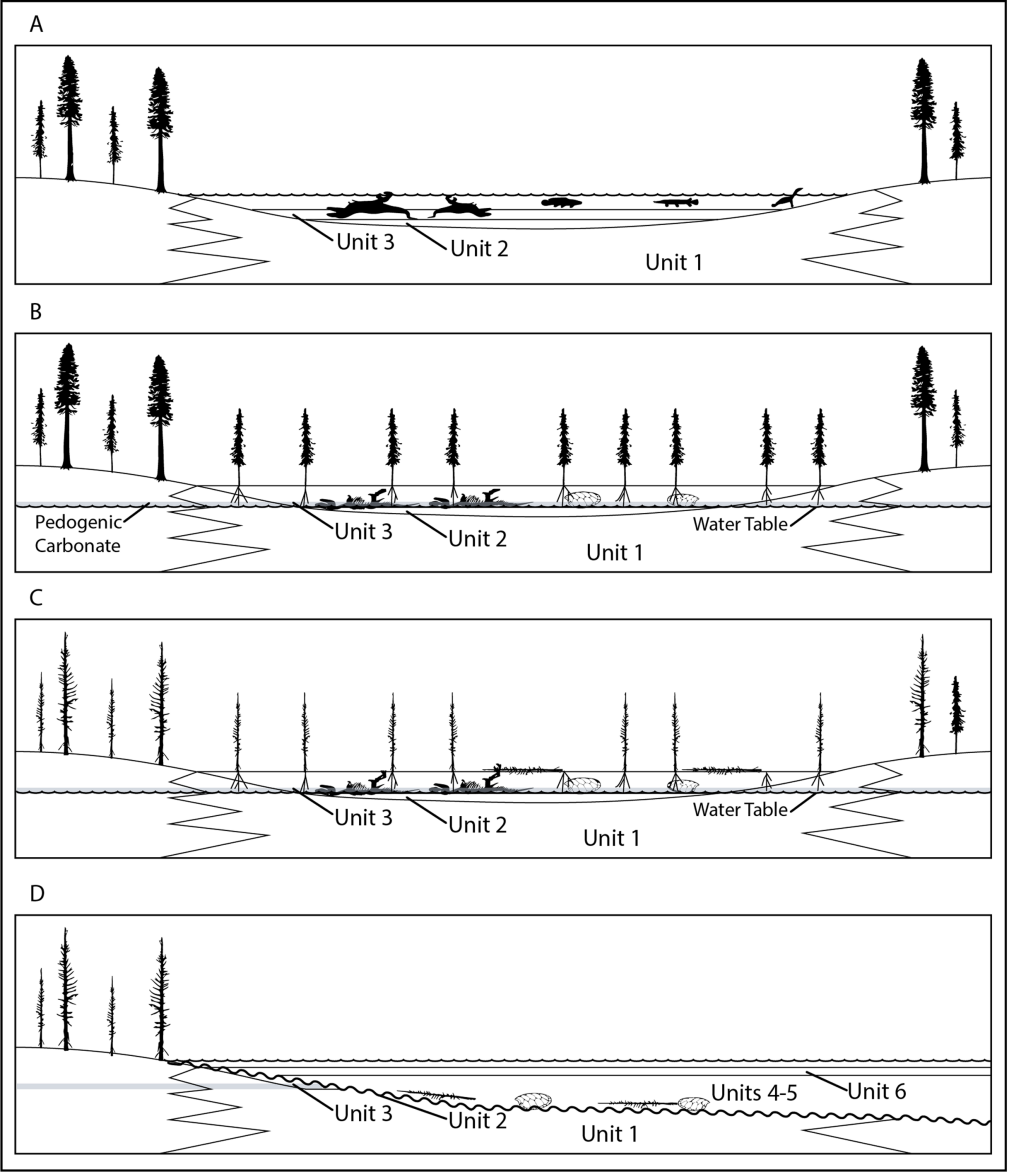
Four key pre-diagenetic stages interpreted for RUQ bonebed development. (A) Deposition of vertebrate and other fossils in the context of a low energy lake setting. (B) Lowering of water table and colonization of the former lake area by plants, accompanied by formation of pedogenic carbonate nodules at and below the bone layer. (C) Lower temperature fire event creating hydraulic instability in the region. (D) Avulsion of nearby river channel leading to exhumation and reburial of the former lake and floodplain fossils into a channel setting.

**Overview:** Although potentially an important data point in arguments regarding gregarious behavior in tyrannosaurids, such inferences from RUQ must first be filtered through the site’s geologic and taphonomic history to try and determine if the individuals represent a recycled single point accumulation or an attritional assemblage washed into units 4 and 5 from widely scattered sources. As a starting point, we reiterate our interpretation that units 1–3 record establishment of a relatively large peri-fluvial lake (probable oxbow) following either a crevasse splay event or, more likely, channel abandonment, since unit 1, which grades into cross-bedded sandstone and thickens southwards, is a fluvial deposit (Fig. 19A). Although it appears much of the upper 2 m of this lake’s sedimentary record was winnowed away prior to deposition of units 4 and 5, certain key pieces of evidence allow us to outline its history prior to paleosol development. The lake hosted large and small aquatic vertebrate fauna, probably augmented with larger, anadromous riverine taxa during warm season flooding events. Stable isotopes in the turtles and fish suggest normal freshwater habitat. The unweathered condition (weighted average taphonomic grade of 1.23) of most vertebrate fossil material (and nearly all tyrannosaur elements) indicates rapid burial and rules processes where the majority of skeletal elements would be exposed to prolonged subaerial weathering at any time. Although our sample size is limited, taphonomic values for aquatic components suggest that at least some of the turtles in units 4 and 5 (weighted average = 1.52) represent a longer standing, more attritional portion of the assemblage, with the more heavily decomposed components matching the condition of the few turtle remains recovered from unit 3. Slightly lower values for the fish material (weighted average 1.25) may indicate presence of a detectable component from a later mass mortality. Terrestrial components show a clear dichotomy, with hadrosaur values, as far as we can tell, representing a single widely scattered juvenile/subadult individual (weighted average = 1.73), reflecting a longer residence/decompositional history than either tyrannosaurs (weighted average 1.05) or the paravian (weighted average 1.0). Lastly, although we do not know exactly how the tyrannosaurs were introduced into this depositional setting, lack of evidence for all but minimal transport after exhumation and the tight spatial grouping of all individuals demonstrates initial burial was in close proximity (within a few meters). Although lacking the amount and diversity of associated vertebrate fauna and subsequent reworking, we see possible analogies with another closely associated, partly-articulated Kaiparowits *Teratophoneus* specimen (UMNH VP 16690) excavated from a fine grained mollusk-rich flood basin lake deposit similar to unit 3 (Wiersma & Loewen, 2013). Most notably, this specimen similarly lacks evidence for predation, scavenging or subaerial weathering.

Despite the presence of large aquatic vertebrates, the size distribution of the RUQ unionids (no greater than 2 cm long) suggests the lake became inhospitable to them, probably through hypoxia, within 10 years of its origin based on comparisons with unionid life cycles in tropical to subtropical South America (Beasley et al., 2000; Callil et al., 2017). Tropical wet-dry lake systems become susceptible to anoxia-inducing eutrophication/hypereutrophication in the warm season as they shallow because of a combination of nutrient loading from volume reduction and overall warmer temperatures which both promote phytoplankton blooms, and lower oxygen carrying capacity of water (Bleich, Silveira & Nogueira, 2009). Such conditions are universally lethal to juvenile unionids (Dimock & Wright, 1993; Sparks & Strayer, 1998). The RUQ juvenile unionid mortality is most likely the first indicator of diminishing refresh by warm season flood pulses, which precipitated the downward trend of the lake’s ecological health and ultimate drying out. Abundance of gar and amiid fish remains, both low oxygen tolerant taxa (Smatresk & Cameron, 1982; Porteus, Wright & Milsom, 2014), could reflect an acme during a later hypoxic phase of the lake following unionid die-off.

Presence of pedogenic carbonate nodules, pervasive wet-dry cracking of skeletal material, and preservation of in situ roots in unit 3 all reflect the transition of the initial bonebed from a lake deposit into a protosol, at which point all skeletal elements and the carbonate nodules were imparted with their HREE depleted REE signatures. Although the former lake would probably flood seasonally, it appears it never re-established itself permanently following the initiation of pedogenesis. No in situ roots were observed to have diameters wider than 5–6 cm, so invasion of the former lake area by plants appears to have arrested at an early stage (possibly from drought and/or fire), saving the bone from being consumed in the soil. Overall low levels of REEs in the entire fossil deposit also suggest a relatively short pedogenic phase.

Following protosol formation, the entire lake complex was subject to winnowing/erosion and redeposition into units 4 and 5 with minimal transport of clasts. The high density and diversity of fossil material is typical of floodplain accumulations reworked into fluvial channels (Rogers & Kidwell, 2007), a view supported by our collective sedimentological, taphonomic, stable isotopic and REE data. Finally, extensive damage/breakage of fossil material occurred during the single exhumation and reburial event, and not during the initial accumulation phase. Given these constraints, we review six possible scenarios for creating the tight spatial grouping of the RUQ tyrannosaurid individuals with the a priori assumption it is an intrinsic biogenic concentration sensu Rogers & Kidwell (2007). A discussion of each is given below.

**Mire:** Sediment mires are ephemeral traps typically created as shrinking bodies of still standing water expose areas of deep mud accumulation, which is compatible with our drying lake hypothesis. Prolonged struggles of trapped organisms produce characteristic ichnological deformation structures (fugichnia) in entrapping layers (Varricchio et al., 2008). If larger prey animals become mired, these could hypothetically draw predators that also get trapped and accumulate over time (Spencer, VanValkenburgh & Harris, 2003). However, a survey of Holocene to Miocene large mammal mass-mortality sites (Berger et al., 2001) identified sediment mires as the cause in only four out of a total of fifty-one and none of them had trapped predators. Furthermore, sediment mires tend to be relatively thin, selectively trapping smaller animals and juveniles (Berger et al., 2001; Varricchio et al., 2008; Eberth, Xing & Clark, 2010). Although supposed examples of miring larger animals exist (Sander, 1992; Hungerbühler, 1998), the largest animals Berger et al. (2001) could confirm had been trapped in more recent sedimentary mires were modern moose (*Alces alces*). Additionally, miring of large-bodied vertebrates results in preservational bias towards body parts trapped in sediment such as hindlimbs and portions of the posterior trunk. Skulls, upper body and distal tail portions are generally absent (Burrows, 1989; Hungerbühler, 1998; Sander, 1992). Given the abundance of skull material at the RUQ, complete lack of bias in skeletal representation (Fig. 16) and that the original bone hosting layer couldn’t have been more than 2 m thick, we rule out miring as a mechanism.

**Quicksand/Liquefaction**: Quicksand and seismic liquefaction have been hypothesized as mechanisms for spatial concentration of theropods either through mass kills or time attritional accumulations (Eberth, Xing & Clark, 2010; Kirkland et al., 2016). Both result in formation of contorted/vertically disrupted bedforms and other highly distinctive sedimentary structures that typically have meter scale relief. No such structures are observed to exist in unit 3, and it seems unlikely that any overlying units, with their 2 m estimated maximum thickness, had them either. Consequently, we eliminate this as a possible mechanism.

**Poisoning:** Tight spatial groupings of large animals can hypothetically be created by poisoning at shallow, generally smaller scale bodies of water if the conditions are right to generate lethal levels of toxins (Briand et al., 2003). The two primary agents of poisoning are bacteria of the genus *Clostridium* and toxin-producing cyanobacteria (Varricchio, 1995). *Clostridium* poisoning is selective towards insectivores and carnivores that consume tainted maggots or carcasses, and tyrannosaurids are potential candidates. However, intravenous injections of massive amounts of *botulinum* toxins cannot kill a mouse in less than 20 min (Boroff & Fleck, 1966), and most animals, including birds, do not present any symptoms within less than a few hours of normal toxin ingestion (Friend & Franson, 1999) leading us to conclude that dropping five tyrannosaurs of highly disparate body masses in the exact same spot via slow onset *botulinum* poisoning would be a highly improbable event. Cyanobacterial poisoning is less selective as the water itself becomes toxic and kills animals coming to drink. The two most common and lethal cyanobacterial toxins are peptide and alkaloid hepatotoxins and neurotoxins (Briand et al., 2003). Because of slow onset mortality, cyanobacterial hepatotoxins are rarely, if ever, responsible for creating accumulations of multiple large animals in the exact same spot (Bengis et al., 2016). Like *botulinum* poisoning, we view this as a highly unlikely accumulation mechanism at the RUQ. Alkaloid neurotoxins do have the potential to present symptoms and kill animals within minutes of ingestion (Carmichael, Biggs & Gorham, 1975). Our hypothetical “dying lake” could have ultimately created the type of shallow, eutrophic warm body of water conducive to generating neurotoxic cyanobacterial blooms. Thus, we cannot rule out cyanobacterial neurotoxicosis as a cause of death/accumulation for the tyrannosaurs. However, numerous images from a recent cyanobacterial neurotoxin-induced elephant mass-mortality of over 350 individuals in Botswana (Azeem et al., 2020) show that carcasses are widely dispersed around poison sources because large body size delays mortality long enough that affected individuals still have time to wander away. Thus, we remain skeptical that even neurotoxins could create a literal “pile” of tyrannosaur victims whether as a mass kill or over an extended time, although a subsequent flood could collect their carcasses if it happened almost immediately after poisoning.

**Drought**: Drought is commonly invoked as a mechanism for creating exclusively attritional bonebeds around former water holes or springs (Rogers & Kidwell, 2007), and has long been held as the cause of numerous dinosaur fossil concentrations in the Upper Jurassic Morrison Formation, including the famous Cleveland-Lloyd *Allosaurus fragilis* site (Gates, 2005) and the mixed assemblage at the Carnegie Quarry, Dinosaur National Monument (Carpenter, 2013). The upper Campanian Two Medicine Formation, nearly coeval with the Kaiparowits Formation, has also had drought proposed as a mass mortality/concentration trigger (Rogers, 1990; Varricchio, 1995). Signatures of drought kill include mixed terrestrial taxa (all animals are forced to remnant water sources), bias towards juveniles in the assemblage (Conybeare & Haynes, 1984), and indicators of arid or semi-arid climate (Gates, 2005). Unlike the Morrison Formation, which has long been held to represent largely semi-arid environments (Demko, Currie & Nicoll, 2004; Tanner, Galli & Lucas, 2014), the middle unit of the Kaiparowits Formation does not contain calcretes, red or purplish beds, arid flora (Roberts, 2007; Miller et al., 2013), or other indicators of persistent dry climate. Instead, the Kaiparowits Formation reflects a humid/mesic wetlands environment (Roberts, 2007; Crystal et al., 2019) where trunk stream systems would rarely, if ever, dry out even if local bodies of water did. One would also expect drought kill remains to have prolonged subaerial exposure and exhibit greater degrees of weathering than observed at RUQ. Lastly, the tyrannosaurid assemblage represents a relatively continuous ontogenetic spectrum (Fig. 15) as well as a disproportionate share of the dinosaur assemblage, all of which argues against drought kill (Varricchio, 1995).

**Fire**: Presence of charcoal in units 4 and 5 suggests fire may have been responsible for the tyrannosaurid deaths, possibly even driving individuals into a forced concentration. Indeed, the RUQ site does share many similarities with the Upper Triassic Snyder Quarry bonebed in north-central New Mexico, which was interpreted by Zeigler (2003) as a fire-induced mass mortality. Both sites exhibit charcoal, a mix of aquatic and terrestrial vertebrates, a lack of significant size sorting, widespread disarticulation, and reworking of material into a fluvial unit. Estimated fire temperatures from the Snyder Quarry also fall within the range estimated for the RUQ site.

While we see the sites as possibly analogous, two lines of evidence argue against fire being responsible for killing the tyrannosaurids at RUQ. Firstly, although making analogies between tropical Mesozoic gymnosperm forests and their angiosperm-dominated Neogene counterparts is imperfect at best, in modern warm, humid/mesic settings such as are interpreted for the Kaiparowits Formation, fire is an unusual phenomenon resulting from extended dry (cool) seasons that lower fuel moistures to the point where natural ignition from lightning triggers combustion (Bush et al., 2007). Such fires are characteristically low temperature, confined to forest floors, and typify today’s Pantanal and upper Amazon Basin regions where peak occurrences/severity averages every 5–10 years (De Sá Arruda et al., 2016). It is not possible to determine whether the charcoal at RUQ represents one or multiple fire events, though the homogeneity of reflectance might favor a single event. Also, the style of fire (crown, surface or ground) cannot be deduced conclusively, although reflectance data combined with evidence of sub-aerial exposure and senescence of some of the wood indicate a low severity, not particularly fast moving or catastrophic surface fire that burned conifer dominated vegetation during an extended dry season (Fig. 19C). Second, the only possible indicators of extended drought we see at the RUQ site are the pedogenic calcite nodules and there could be a connection between local water table depression stressing vegetation and fire. In this scenario the timing of fire significantly post-dates the initial burial of the tyrannosaurids, since evidence for drying (pedogenic carbonate nodules) formed within already buried bone, ruling it out as an agent of mortality. At the very least, the anomalous lack of foliage in units 4 and 5, very unusual for Kaiparowits Formation bonebeds, argues the reburial of fossils occurred while the area was devoid of dense cover. Modern surface fires often dramatically alter soil permeability and stability, which combined with the removal of vegetation as a rainfall-intercept mechanism, frequently leads to greatly increased surface run-off, erosion, and sediment transport (Brown, Collinson & Scott, 2013; Santín & Doerr, 2016). Such massive, widespread hydrologic chaos could trigger an avulsion event such as that observed at the RUQ and has been implicated in the post-mortem preservation of numerous bonebeds in the Barremian age Wessex and Campanian age Dinosaur Park formations (Sweetman & Insole, 2010; Brown, Collinson & Scott, 2013). Other fire-mediated causes of avulsion, such as clogging of nearby fluvial channels with downed wood, are also plausible. Even though this is, in our view, the most likely scenario, we admit the fire(s) could have occurred at any time between deposition of units 3 and 4. Since charcoal does not occur in unit 3, if fire occurred before the lake sediment’s pedogenic phase, it would have been buried initially in the bone-hosting unit and reworked along with all the other fossils. Although we cannot rule out fire as a killing agent, all evidence points to a slow-moving, lower temperature and severity event the likes of which are rarely implicated in mass killings of large animals (Lyon, Telfer & Schreiner, 2000).

In summary, our preferred interpretation is that fire played a key role in destabilizing soils and triggering the avulsion that exhumed and reburied the bones (Fig. 19D) but was not responsible for the tyrannosaur deaths; a pattern similar to, but not identical with, that outlined by Brown, Collinson & Scott (2013) and Sweetman & Insole (2010) for the preservation of several Dinosaur Park Formation and Wessex Formation dinosaur bonebeds. Thus, rather than seeing the Snyder Quarry as an argument for fire kill of the RUQ tyrannosaurids, the former is much more likely a product of drought or flood kill on top of an attritional aquatic assemblage that was later reworked, possibly because of fire alteration of the landscape’s hydraulic response, which Zeigler (2003) did present as an alternate hypothesis.

**Figure 20:**
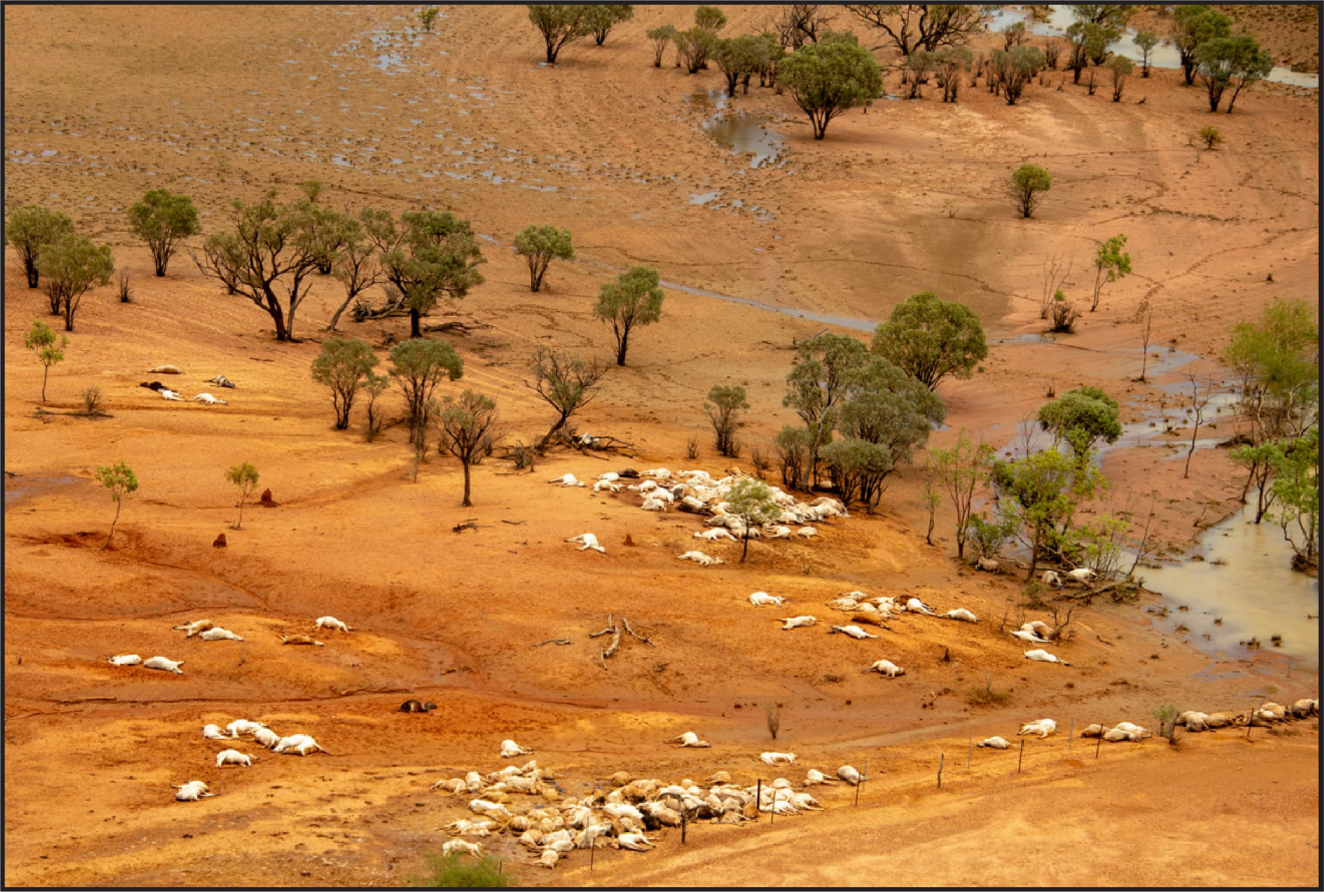
Photograph of accumulated drowned cattle carcasses following massive floodplain flooding in 2019, Queensland, Australia. Note concentration of carcasses around margins of the lower elevation channel, analogous with the oxbow setting hypothesis we propose for the RUQ. Photo credit: The Fence Post Magazine online version.

**Flooding**: Flooding could potentially concentrate tyrannosaur carcasses through two processes: (1) direct kill (drowning) and transport of carcasses into a flood plain catchment (strand or winnow) or (2) transport of a pre-flood accumulation of carcasses (dispersed or concentrated) into a catchment. Direct flood kill mass drowning on flood plains (Fig. 20) is a common cause of large vertebrate mortality and carcass concentration in the natural world (Rogers & Kidwell, 2007), with mortality usually highest near levee failures where water depths and current strengths are greatest (Wuczyński & Jakubiec, 2013). Evidence at the RUQ consistent with a flood-accumulation hypothesis includes the ontogenetic spectrum of tyrannosaurids (i.e., lack of bias in take), monodominant taphonomic mode, lack of selective preservational bias of skeletal elements, and association with abundant aquatic fauna. Given the Kaiparowits ecosystem was mesic/humid (Roberts, 2007) and subject to high seasonal rainfall (Fricke, Foreman & Sewall, 2010; Miller et al., 2013; Crystal et al., 2019) and flood pulse hydrology (Foreman et al., 2015), it seems reasonable to assume flood concentration of carcasses was a common occurrence. Seasonal flooding has been identified as the cause of nearly every Campanian age mass dinosaur concentration in both the Oldman and Dinosaur Park formations (Wood, Thomas & Visser, 1988; Ryan et al., 2001; Eberth & Getty (2005); Chiba et al., 2015), affecting both near coastal and more inland settings. Concentration of carcasses in lower areas of floodplains by such events is also a common outcome (Fig. 20). Lastly, a closely associated/articulated specimen of *Teratophoneus* (UMNH VP 16690) recovered from a mollusk-rich mudstone very similar to RUQ unit 3 a few kilometers away (Wiersma & Loewen, 2013) was determined to have been washed into a flood basin lake or pond during a seasonal crevasse splay flooding event, so the exact process we propose for initial accumulation has been documented to have happened to a single individual of the same genus in the Kaiparowits Formation. Given REE data argue the RUQ site does not represent a long nerm attritional assemblage and the lower likelihood of fire or cyanobacterial toxicosis as causal agents, the preponderance of evidence points toward flooding as the most parsimonious explanation for the concentration of tyrannosaur carcasses at the RUQ.

## Comparisons with two medicine and horseshoe canyon (dry island) sites

Comparison of the RUQ with the two other published Western Interior tyrannosaurid bonebeds offers an opportunity to highlight similarities and differences. All three sites preserve individuals of widely varying ages. Flooding has long been held as the cause of the Dry Island tyrannosaurid concentration, which preserves a minimum number of 12–14 *Albertosaurus* individuals (Eberth & Currie, 2010) initially deposited in a fine grained interfluvial setting (carbonaceous, plant-rich mudstone), and subsequently partially exhumed/winnowed and reburied by a minor (15 m wide) channel incision event which size-sorted the assemblage and redeposited fossil material on lateral accretion surfaces. Multiple aspects of the Dry Island site, including disarticulation, exhumation and reburial, presence of multiple size classes of tyrannosaurids, and presence of charcoal (Currie, personal communication, 2021) are closely analogous with the RUQ. The presence of sturgeon remains at the Dry Island site (Eberth & Currie, 2010) opens the possibility that flooding events concentrating tyrannosaurs at both sites were sourced from major trunk streams. Most differences between Dry Island and RUQ can probably be explained by their fundamentally different paleogeographic contexts. For instance, the mudstone serving as the initial bone host at Dry Island is gastropod and plant-rich (including foliage) but contains relatively little aquatic vertebrate fauna (fish are a minor component and turtles are essentially absent) indicating it represents a small, shallow, relatively ephemeral body of water. Abundant taxodiaceous trunk and foliage content (Eberth & Currie, 2010) also suggests a drowned forest rather than the oxbow or persistent flood basin lake setting we interpret for RUQ. Furthermore, the incision event that reworked RUQ material into units 4–5 was much larger (channel width estimated at >0.3 km vs. 15 m) than the Dry Island event, while the pedogenic calcite nodules at RUQ (vs. siderite at Dry Island) suggest more extreme fluctuations in annual precipitation and possibly a more inland setting.

The *Daspletosaurus horneri* bonebed in the Two Medicine Formation (TA 1997.002) has not been assigned a specific cause (Currie et al., 2005) but given lack of evidence for fire, flood accumulation is certainly tenable. Preservation of multiple tyrannosaurids in a fine-grained mudstone attributable to recurring flood events (Currie et al., 2005) is similar to our hypothesized initial deposition at RUQ, but like Dry Island, TA 1997.002 lacks an abundant large aquatic fauna. The variety of preservational modes at the Two Medicine site, including articulated sections of skeletons, a lack of element sorting, and association of several individual size/ontogenentic classes are like those at RUQ. Since TA 1997.002 also contains several hadrosaurs (Currie et al., 2005) that exhibit evidence of having been fed upon (e.g., shed teeth and bite marks on bones), it is possible this association reflects mortality during an opportunistic feeding event, the cause of which is unknown. In the absence of a more rigorous taphonomic evaluation of the Two Medicine site, current evidence indicates more similarities than differences with both the Dry Island and RUQ sites.

## Conclusions

We are relatively certain that the RUQ tyrannosaur carcasses were very closely spaced during initial burial and conclude the most probable agent of accumulation is flood transport into a quiet water oxbow lake setting (Fig. 19A). This interpretation is generally consistent with those published for Dry Island, TA 1997.002, and another Kaiparowits Formation tyrannosaur locality described by Wiersma & Loewen (2013). Although we cannot rule out cyanobacterial neurotoxicosis or fire as causal agents of tyrannosaur take/concentration at the RUQ, we regard these as much less likely. Because the Dry Island and TA 1997.002 sites have been used as arguments for mass mortality and inferred gregariousness in tyrannosaurids (Currie, 1998; Currie & Eberth, 2010), this opens the possibility that the RUQ site could also evidence such behavior. McCrea et al. (2014) described a nearly coeval (Wapiti Formation) tyrannosaurid tracksite (Fig. 1) in British Columbia made by multiple individuals closely associated in time and argued it likewise reflected tyrannosaur gregariousness. Although we cannot ascertain whether the RUQ tyrannosaurs died in a mass-mortality or were transported from disparate localities over a relatively short duration, low taphonomic values observed on all tyrannosaur elements demonstrate their deaths were not widely separated in time. This leaves mass mortality as a viable explanation for the RUQ bonebed and opens the possibility this is the first evidence of such in southern Laramidia. Furthermore, while each Campanian or Maastrichtian age tyrannosaur mass burial site considered separately represents a statistically rare (=improbable) event, we see no reason why this broadening pattern in eastern Laramidia across completely different paleogeographic/paleoecological settings and among different clades couldn’t reflect innate behavior such as habitual gregariousness.

## Supplemental Information

10.7717/peerj.11013/supp-1 Supplemental Information 1 Bulk composition and clay species determinations by x-ray diffraction for unit 3, unit 4 mudstone clasts, and unit 4 bone mudstone fill. Bulk composition and clay species determinations by x-ray diffraction for unit 3, unit 4 mudstone clasts, and unit 4 bone mudstone fill.

10.7717/peerj.11013/supp-2 Supplemental Information 2 Raw data for phosphate analysis of carbon and oxygen isotopes.

10.7717/peerj.11013/supp-3 Supplemental Information 3 REE analysis data for bone and pedogenic carbonate at RUQ.

10.7717/peerj.11013/supp-4 Supplemental Information 4 Summary of the number of locations measured to determine mean random charcoal reflectance (Ro%). Asterisk (*) indicates a sample measured at less than 40 locations and so considered less statistically robust. Ro_HO_ is the highest observed reflectance in any given sample and was used to calculate the mean charring temperature of each specimen (°C).

10.7717/peerj.11013/supp-5 Supplemental Information 5 Tabulated list of identified fish specimens from the RUQ.

10.7717/peerj.11013/supp-6 Supplemental Information 6 Tabulated list of identified turtle specimens from the RUQ.

10.7717/peerj.11013/supp-7 Supplemental Information 7 Tabulated list of identified crocodylian (Neosuchia) specimens from the RUQ.

10.7717/peerj.11013/supp-8 Supplemental Information 8 Tabulated list of identified dinosaur specimens from the RUQ.

10.7717/peerj.11013/supp-9 Supplemental Information 9 Weathering and breakage data for vertebrate fossils specimens from the RUQ. Taphonomic indices are based on Behrensmeyer, 1991 and are given in values of 1-5. No 5 values were observed. Breakage patterns and the percent of area covered by fractures were both recorded.

## Additional Information and Declarations

### Data Availability

The following information was supplied regarding data availability: Data are available in the Supplemental Files and include charcoal sampling sites, fracture patterns, and taphonomic indices. All non-consumable specimens used in this study are reposited at the Natural History Museum of Utah under accession numbers UMNH.A.2014.82; UMNH.A.2016.9; UMNH.A.2017.14; UMNH.A.2018.21;UMNH.A.2018.35; and UMNH.A.2020.3. Individual specimens have not received UMNH specimen numbers yet, but are still identified by their initial catalog numbers assigned at the BLM laboratory in Kanab, Utah (14UTKA-8-1 through 14UTKA-8-399; 14UTKA-8-400 through 14UTKA-8-599; 14UTKA-8-700 through 14UTKA-8-1112; 14UTKA-8-1200 through 14UTKA-8-1208). Identified specimen numbers are provided in the NISP tables (Tables S3–S6). The remaining specimens (highly fragmentary or non-diagnostic) are not directly relevant to the current work are available for examination on request to the Paria District Paleontologist (Dr. Alan Titus-atitus@blm.gov), Kanab, Utah. Contact can also be made through the following website: https://www.blm.gov/office/paria-river-district.
