# Supplementary material for: Geology and taphonomy of a unique tyrannosaurid bonebed from the upper Campanian Kaiparowits Formation of southern Utah: implications for tyrannosaurid gregariousness
Source: PeerJ. 2021 Apr 19;9:e11013. doi: 10.7717/peerj.11013 (PMC8061582; doi:10.7717/peerj.11013)
Supplement: Supplemental Information 2 [file peerj-09-11013-s002.pdf]

sample	unit	calcite matrix	$\delta^{18}O$ VPDB	$\delta^{13}C$ VPDB
KPC-1	Unit 3	Nodule (bulk)	-6.11	-9.26
KPC-2	Unit 4	Nodule in Conglomerate	-7.79	-7.1
KPC-3	Unit 4	Nodule in Conglomerate	-7.58	-7.76
N2-1	Unit 3	spar (microsample)	-7.32	-9.28
N2-2	Unit 3	spar (microsample)	-7.94	-8.48
N2-3	Unit 3	micritic (microsample)	-6.87	-9.48
N2-4	Unit 3	micritic (microsample)	-7.05	-9.27
N2-5	Unit 3	micritic (microsample)	-7.1	-9.27
N2-6	Unit 3	micritic (microsample)	-6.87	-9.16
N1-1	Unit 3	spar (microsample)	-8.38	-9.68
N1-2	Unit 3	spar (microsample)	-6.44	-10.16
N1-3	Unit 3	spar (microsample)	-7.37	-9.76
N1-4	Unit 3	spar (microsample)	-6.16	-9.87
N1-5	Unit 3	micritic (microsample)	-7.94	-8.88
N1-6	Unit 3	micritic (microsample)	-7.93	-8.71
N1-7	Unit 3	micritic (microsample)	-8	-8.78
N1-8	Unit 3	micritic (microsample)	-7.84	-8.9
KF-clam-1	Unit 3	Unionid	-8.89	-5.46
KF-clam-2	Unit 3	Unionid	-8.96	-5.2
KF-clam-3	Unit 3	Unionid	-8.95	-4.71
KF-clam-4	Unit 3	Unionid	-9.47	-5.03
KF-clam-5	Unit 3	Unionid	-7.49	-4.63
KF-clam-6	Unit 3	Unionid	-8.38	-4.84
KF-clam-7	Unit 3	Unionid	-8.15	-5.08
KF-clam-8	Unit 3	Unionid	-8.36	-4.89
KF-clam-9	Unit 3	Unionid	-8.11	-5.09
KF-clam-1(	Unit 3	Unionid	-8.46	-4.76
