# Supplementary material for: Geology and taphonomy of a unique tyrannosaurid bonebed from the upper Campanian Kaiparowits Formation of southern Utah: implications for tyrannosaurid gregariousness
Source: PeerJ. 2021 Apr 19;9:e11013. doi: 10.7717/peerj.11013 (PMC8061582; doi:10.7717/peerj.11013)
Supplement: Supplemental Information 3 [file peerj-09-11013-s003.pdf]

sample	taxa	material	140Ce	141Pr	146Nd	147Sm	153Eu	157Gd
1	ganoid scale	dentine	393.73	55.01	218.19	39.29	10.17	44.41
2	ganoid scale	dentine	376.49	60.01	241.45	40.89	10.11	43.88
3	ganoid scale	dentine	542.39	86.28	344.50	60.94	15.44	67.00
M4	tyrannosaur	dentine	470.82	58.66	218.06	36.91	9.13	40.81
740A	Giloremys	costal	1031.42	125.39	490.44	95.82	25.91	114.01
136	tyrannosaur	dentine	1008.12	118.74	464.99	89.35	26.28	120.51
N1	Neurankylus	carapace	37.24	5.62	23.23	3.45	0.89	4.37
894	Deinosuchus	dentine	2348.63	302.84	1169.29	232.20	55.84	249.38
440	turtle	carapace	209.27	23.49	83.60	12.14	3.48	17.25
454	turtle	carapace	186.74	22.50	82.56	12.39	3.70	16.78
nod 1	carbonate	nodule in bone	110.98	12.58	48.91	8.95	2.64	12.29
unit 4 nod	carbonate	unit 4	16.36	2.11	8.15	1.60	0.41	1.88
fish vert	fish	vertebrate	1377.54	180.40	721.07	143.64	39.51	163.03
G1	tyrannosaur	vertebrate	674.18	70.65	268.86	47.36	14.35	68.75
150	tyrannosaur	dentine	848.53	107.20	420.92	84.87	21.71	103.47

159Tb	163Dy	165Ho	166Er	169Tm	172Yb	175Lu	Total
5.10	21.82	3.59	8.35	0.64	3.01	0.35	803.65
4.73	18.72	3.01	6.97	0.45	2.02	0.26	808.99
7.58	31.60	5.19	11.96	0.85	3.86	0.47	1178.07
4.53	18.78	3.13	7.67	0.61	2.99	0.38	872.47
14.64	70.18	12.58	31.05	2.94	14.43	1.92	2030.73
15.27	74.16	13.30	31.68	2.81	13.15	1.71	1980.07
0.44	1.77	0.29	0.67	0.04	0.20	0.03	78.24
32.05	147.28	24.98	61.28	5.73	28.61	3.98	4662.10
1.88	8.87	1.82	4.87	0.43	1.96	0.29	369.36
1.84	8.53	1.71	4.51	0.39	1.82	0.27	343.73
1.55	8.19	1.54	3.95	0.40	2.11	0.30	214.40
0.25	1.28	0.25	0.71	0.08	0.50	0.07	33.65
20.13	87.96	14.50	34.01	2.73	12.95	1.71	2799.19
8.18	38.61	7.43	18.81	1.76	8.94	1.27	1229.14
12.99	59.16	10.18	24.09	2.06	9.72	1.31	1706.20
