# Supplementary material for: Geology and taphonomy of a unique tyrannosaurid bonebed from the upper Campanian Kaiparowits Formation of southern Utah: implications for tyrannosaurid gregariousness
Source: PeerJ. 2021 Apr 19;9:e11013. doi: 10.7717/peerj.11013 (PMC8061582; doi:10.7717/peerj.11013)
Supplement: Supplemental Information 4 — Asterisk (*) indicates a sample measured at less than 40 locations and so considered less statistically robust. RoHO is the highest observed reflectance in any given sample and was used to calculate the mean charring temperature of each specimen (°C). [file peerj-09-11013-s004.docx]

| **Sample #** | **No. points measured** | **Mean Ro% ± 1s.d.** | **Ro%_HO_** | **Min Temp. °C calc. from Ro%_HO_** |
| --- | --- | --- | --- | --- |
| 15C-1A | 100 | 1.61 ±0.10 | 1.84 | 461 |
| 15C-1B | 100 | 1.50 ±0.10 | 1.69 | 449 |
| 15F-AA | 16* | 1.14 ±0.04 | 1.19 | 402 |
| 15F-AB | 18* | 0.99 ±0.04 | 1.05 | 387 |
| 15F-AC | 33* | 1.15 ±0.03 | 1.2 | 404 |
| 15F-B | 100 | 1.39 ±0.06 | 1.54 | 437 |
| 16A-AA | 100 | 1.24 ±0.08 | 1.42 | 426 |
| 16A-AB | 100 | 1.16 ±0.06 | 1.28 | 412 |
| 16A-BA | 5* | 0.90 ±0.02 | 0.93 | 373 |
| 16A-BB | 5* | 0.76 ±0.03 | 0.8 | 356 |
| 16A-BC | 5* | 0.77 ±0.04 | 0.83 | 360 |
| 17C-1A | 100 | 1.72 ±0.17 | 2.1 | 479 |
| 17C-1B | 100 | 1.83 ±0.08 | 2.02 | 473 |
| 17C-2 | 100 | 1.30 ±0.12 | 1.59 | 441 |
| 17C-3A | 100 | 0.79 ±0.07 | 0.96 | 377 |
| 17C-3B | 100 | 1.07 ±0.09 | 1.27 | 412 |
| 17C-3-Strew1 | 40 | 1.34 ±0.09 | 1.53 | 436 |
| 17C-3-Strew2 | 10* | 1.16 ±0.04 | 1.21 | 404 |
| 17C-3-Strew3 | 10* | 1.18 ±0.10 | 1.23 | 407 |
| 17C-3-Strew4 | 50 | 1.12 ±0.04 | 1.35 | 419 |
| 17C-3-Strew5 | 10* | 1.56 ±0.07 | 1.19 | 403 |
| 17C-3-Strew6 | 45 | 1.50 ±0.06 | 1.73 | 453 |
| 17C-3-Strew7 | 100 | 1.12 ±0.05 | 1.63 | 444 |
| 17C-3-Strew8 | 10* | 1.08 ±0.05 | 1.2 | 404 |
| 17C-3-Strew9 | 10* | 1.53 ±0.10 | 1.18 | 401 |
| 17C-3-Strew10 | 100 | 1.67 ±N/A | 1.75 | 454 |
| 17C-4A | 2* | 1.12 ±0.07 | 1.67 | 448 |
| 332A-A | 100 | 1.13 ±0.05 | 1.26 | 410 |
| 774A-A | 100 | 1.29 ±0.10 | 1.26 | 410 |
| X-A | 100 | 1.61 ±0.10 | 1.47 | 430 |

Table 5. A summary of the number of locations measured to determine mean random reflectance (Ro%), an asterisk (*) indicates a sample measured at less than 40 locations and so considered less statistically robust. Ro_HO_ is the highest observed reflectance in any given sample and was used to calculate the mean charring temperature of each specimen (°C).
