# Supplementary material for: Geology and taphonomy of a unique tyrannosaurid bonebed from the upper Campanian Kaiparowits Formation of southern Utah: implications for tyrannosaurid gregariousness
Source: PeerJ. 2021 Apr 19;9:e11013. doi: 10.7717/peerj.11013 (PMC8061582; doi:10.7717/peerj.11013)
Supplement: Supplemental Information 5 [file peerj-09-11013-s005.pdf]

Spec. #	Element ID	Grid Ref.	Lepisost.	Amiid	Teleost
1018	vert	2E		1	
1020	cranial	2E		1	
1024	vert	2E		1	
1033	cranial	2E		1	
1066	articul. verts	2E			1
1040	tooth	2G	1		
1041	tooth	2G	1		
990	vert	3F		1	
127d	scales	6E	1		
331b	scales and vertebra	8D	1		
396c	vertebrae	6E	1		
83c	scales	7C	1		
east of 15	vertebrae	7C	1		
w/ block 1	vertebrae and skull	3E	1		
554	vert	_F25		1	
541	dentary	_I26		1	
133	articul. verts		1		
			9	7	1
