# Supplementary material for: Geology and taphonomy of a unique tyrannosaurid bonebed from the upper Campanian Kaiparowits Formation of southern Utah: implications for tyrannosaurid gregariousness
Source: PeerJ. 2021 Apr 19;9:e11013. doi: 10.7717/peerj.11013 (PMC8061582; doi:10.7717/peerj.11013)
Supplement: Supplemental Information 6 [file peerj-09-11013-s006.pdf]

Spec. #	Element ID	Grid Ref.	Denazinam	Neurankyl	Gilmore	Aspideretc	Helopan	Panchel	Adocus
45	carapace	3D		1					
46	shell	2D		1					
50	plastron	3A		1					
80	shell			1					
180	shell							1	
309	shell	7A				1			
310	carapace	7A				1			
319	costal	7-A				1			
320		7-A					1		
322	costal	8A				1			
326	marginal	3D						1	
327b	marginal	3D						1	
328	shell	3D		1					
338	shell	8D		1					
339	shell	8D		1					
346b	dentary							1	
371	carapace	5A		1					
401	carapace	_G27		1					
405	peripheral	_I25						1	
406	costal	_I24						1	
407	peripheral	_I24		1				1	
414	shell							1	
412	hypoplastron	_I25				1			
423	costal	_G25		1					
430	shell	_F25		1					
433	shell	_H25						1	
434	shell			1					
441	costal	_H25		1				1	
442	costal	_H25		1					
444	costal			1					
446	shell			1					
450	carapace	_G25						1	
451	shell	_G25		1					
452	shell							1	
453	shell	_G25		1					
454	costal	_G25		1					
464	costal	_H24		1					
467	shell			1					
471	anterior carapace	_H24						1	
475	peripheral	_G24		1					
478	shell	_G24		1					
483	peripheral?	_G24						1	
486	shell	_G24		1					
490	neural	_G24		1					
492	shell			1					
493	neural	_G24		1					
495	costal	_G23		1					
507	shell	_H23		1					

512	shell	_G24		1					
513	shell	_G24		1					
517	shell	_G23		1					
520	shell*	_H23							1
530	shell			1					
558	neural			1					
572	shell								1
575	shell			1					
584	humerus								1
715	shell	9D?		1					
744	costal	10F			1				
751	shell			1					
770	shell	10F		1					
795	shell	10G		1					
975	shell	2F		1					
989	shell			1					
1094	shell						1		
1100	costal					1			
1201	costal	_I24		1					
1203	shell			1					
1204	shell		1						
1205	shell							1	
"from tren	neural	6A or 6B		1					
217b	shell fragment	6C		1					
311A	costal	7A				1			
311B	costal	7A				1			
532b							1		
70B	shell	7C							
740A	pectoral girdle	10G							
740b	costal	10G			1				
745b	costal	10F			1				
NC-1	shell	1E		1					
NHMU1	shell*			1					
NHMU2	shell*			1					
NC-5			1						
NC-2				1					
NC-3				1					
NC-4				1					
NC-6				1					
NC-7				1					
NC-8				1					
NC-9				1					
NC-10				1					
NC-11				1					
NC-12				1					
NC-13				1					
NC-14				1					
NC-15				1					
NC-18				1					

NC-16		1					
NC-17		1					
NC-21		1					
NC-19		1					
NC-20		1					
	2	68	3	8	3	15	3
