# Supplementary material for: Geology and taphonomy of a unique tyrannosaurid bonebed from the upper Campanian Kaiparowits Formation of southern Utah: implications for tyrannosaurid gregariousness
Source: PeerJ. 2021 Apr 19;9:e11013. doi: 10.7717/peerj.11013 (PMC8061582; doi:10.7717/peerj.11013)
Supplement: Supplemental Information 7 [file peerj-09-11013-s007.pdf]

Spec. #	Element ID	Grid Ref.	Deinosuch
877	tooth	2G	1
878	tooth	2G	1
879	tooth	2G	1
880	tooth	2G	1
886	tooth	2H	1
887	tooth	2H	1
888	tooth	2H	1
889	tooth	2H	1
890	tooth	2H	1
1098	tooth	2H	1
1099	tooth	2H	1
901	R dentary	3G	1
870	cranial	3G	1
1004	dorsal vert	3G	1
984	rib	3G	1
985	rib	3G	1
970	osteoderm	3G	1
967	tibia	3G	1
895	tooth	3G	1
894	tooth	3G	1
893	tooth	3G	1
871	tooth	3G	1
870	cranial	3G	1
			23
