# Supplementary material for: Geology and taphonomy of a unique tyrannosaurid bonebed from the upper Campanian Kaiparowits Formation of southern Utah: implications for tyrannosaurid gregariousness
Source: PeerJ. 2021 Apr 19;9:e11013. doi: 10.7717/peerj.11013 (PMC8061582; doi:10.7717/peerj.11013)
Supplement: Supplemental Information 8 [file peerj-09-11013-s008.pdf]

Spec. #	Element ID	Right	Left	Size Class	Grid Ref.	cf Terato	Hadro	Paraves
150I	juvenile tooth, premaxillary?			juve	7E	1		
366	??				6B	1		
83a	??				7C	1		
181	?caudal				7-A	1		
111	?frontal				6A	1		
352b	?hyoid				8E	1		
77a	?lacrimal				8C	1		
110	?neural arch				6A	1		
74	?post orbital				6D	1		
352a	?right lacrimal				8E	1		
113	?sacral fragment				6A	1		
100	anterior max or dentary rooted tooth				4B	1		
52c	appendicular				5D	1		
4	astragalus		x	adult	7C	1		
11	astragalus				6C	1		
142a	atlantal arch			juve	6D	1		
141a	atlas			juve	6D	1		
15-5	bone					1		
761	braincase			juve		1		
57	calcaneum			adult	5C	1		
15F-10	calcaneum	x		juve		1		
34	caudal				4B	1		
35	caudal				4B	1		
36	caudal				4B	1		
101	caudal				5B	1		
473	caudal				_G24		1	
B-2	caudal				4B	1		
701	caudal? centrum				3D	1		
41	centrum						1	
525	centrum				_G24		1	
549	centrum				26H		1	
J-17-1-a	cervical			adult		1		
J-17-1-b	cervical			adult		1		
J-17-1-c	cervical			adult		1		
J-17-1-d	cervical			adult		1		
132	cervical neural arch			juve	5D	1		
384	cervical neural arch					1		
589A	cervical rib					1		
M15	cervical rib 3		x	juve	5C	1		
790	cervical rib?				9G	1		
98	chevron				4B	1		
109	chevron			adult	5A	1		
557	chevron				_H26		1	
15F-18	chevron			juve		1		
15A-13	chevron (prox)			adult		1		
217C	cranial				6C	1		

351a	cranial element				8E	1	
351b	cranial element				8E	1	
169c	cranial fragment				6C	1	
300	dentary and splenial	x		adult	7A	1	
150N	dentary L		x	juve	7E	1	
136a	dentary or maxillary tooth				6E	1	
145b	dentary or maxillary tooth				7E	1	
east of 15l	dentary or maxillary tooth				7C	1	
61	dist caudal				4B	1	
62	dist caudal				4B	1	
31	distal caudal				4B	1	
437	distal caudal				_G25	1	
B-3	distal caudal				4B	1	
B-4	distal caudal				4B	1	
B-5	distal caudal				4B	1	
B-6	distal caudal				4B	1	
B-7	distal caudal				4B	1	
JB-2	distal caudal				4B	1	
JB-3	distal caudal				4B	1	
JB-4	distal caudal				4B	1	
JB-5	distal caudal				4B	1	
60	distal caudal centra (half)				4B	1	
119	distal caudal centrum				4A	1	
136b	distal caudal centrum				6E	1	
52b	distal-caudal centrum				5D	1	
13	dorsal rib				6C	1	
14	dorsal rib				6C	1	
20	dorsal rib		x	adult	5C	1	
53	dorsal rib				5D	1	
143	dorsal rib				7D	1	
164	dorsal rib				6C	1	
196	dorsal rib	x		adult	8C	1	
217	dorsal rib	x		adult	6C	1	
347	dorsal rib				7E	1	
361	dorsal rib				6B	1	
362	dorsal rib				6C	1	
369	dorsal rib				7B	1	
372	dorsal rib				6B	1	
705	dorsal rib				6F	1	
803	dorsal rib				5F	1	
217b	dorsal rib				6C		1
52a	dorsal rib				5D	1	
64	dorsal vert				4C	1	
702	dorsal vertebrae				6F	1	
M6	ectopterygoid	x		juve	5C	1	
40	femur				4B	1	
394	femur L		x	juve	8F	1	

402	femur L		x		_I28		1	
24	femur R	x		juve	4B	1		
1	fibula			small juv		1		
15F-62	fibula	x		juve		1		
58a	gastralia			adult				
118	gastralia				5C	1		
153	gastralia				5A	1		
134a	gastralia				6D	1		
134b	gastralia				6D	1		
214	gastralia?				5B	1		
215	gastralia?				5B	1		
352	gastralia? Cranial?				8E	1		
127	gastralia? dorsal ribs?				6E	1		
134	gastralia? Rib?				6D	1		
116	gastralium			juve	5C	1		
169	gastralium				6C	1		
58-A	gastralium				5B	1		
M12	gastralium			juve	5C	1		
M2	gastralium				5C	1		
M4	gastralium				5B	1		
M7	gastralium			juve	5C	1		
M1	gastralium?				5C	1		
178	indet				5C	1		
321	indet				8A		1	
329	indet				6B	1		
379	indet				8E	1		
559	ischial shaft?				_H27		1	
83	jugal				7C	1		
1207	juvenile femur					1		
150B	juvenile maxillary tooth				7E	1		
150O	juvenile premaxillary tooth				7E	1		
158	angular L	x		juve	4D			1
97	man III-3 L		x					1
75	lacrimal				3C	1		
194	lacrimal	x		juve				
M14	lacrimal	x		juve	5C	1		
375	lacrimal R	x		adult		1		
15F-41	lateral distal tarsal	x		juve		1		
445	lateral metacarpal?				_G26		1	
"north of 1	limb				6A or 6B	1		
150K	long thin process; cranial?				7E	1		
721	maxilla L		x			1		
136a	maxillary or dentary tooth crown				6E	1		
495a	metacarpal						1	
302	metatarsal II	x		adult	7B			
15F-44	metatarsal II L		x	adult		1		
15F-33	metatarsal II R	x		juve		1		

301	metatarsal III	x		adult	7B			
15F-61	metatarsal III L		x	adult		1		
15F-1	metatarsal III R	x		juve		1		
303	metatarsal IV	x		adult	7A	1		
15F-60	metatarsal IV L		x	adult		1		
15F-20	metatarsal IV R	x		juve		1		
102	mid caudal				7-A	1		
103	mid caudal				7-A	1		
104	mid caudal				7-A	1		
105	mid caudal				7-A	1		
106	mid caudal				7-A	1		
107	mid caudal				7-A	1		
float from	mid caudal					1		
709	mid dorsal rib				9F	1		
98	mid to distal caudal vertebrae				4B	1		
324	mid to distal caudal vertebrae				8A		1	
325	mid to distal caudal vertebrae				8A		1	
411	mid to distal caudal vertebrae				_I25		1	
78b	mid-caudal centrum				7-A	1		
202	mid-dorsal rib				7B	1		
217a	mid-dorsal rib				6C	1		
168	mid-to-distal caudal centrum				5A	1		
395	nasal	x	x	adult	8F	1		
719	palatine		x	adult	7F	1		
70a	pedal phalanx I-1							1
22	pedal phalanx II-1		x	adult	5B	1		
15F-15	pedal phalanx II-1		x	juve		1		
15F-35	pedal phalanx II-1	x				1		
728	pedal phalanx II-2	x		small juv	6F	1		
313	pedal phalanx II-2		x	adult	7A	1		
130	pedal phalanx II-2		x	juve	5D	1		
29	pedal phalanx II-2	x		juve		1		
30	pedal phalanx II-2	x		juve	5B	1		
21	pedal phalanx III-1			adult	5B	1		
150h	pedal phalanx III-1			juve	7E	1		
211	pedal phalanx III-1			juve	5B	1		
791	pedal phalanx III-1				6F			1
212	pedal phalanx III-2			juve	5B	1		
213	pedal phalanx III-2			juve	5B	1		
307	pedal phalanx III-2			adult	7A	1		
208	pedal phalanx III-3				5A	1		
86	pedal phalanx IV-1		x	juve	5B	1		
316	pedal phalanx IV-1	x		adult	7B	1		
15F-8	pedal phalanx IV-1	x		juve		1		
209a	pedal phalanx IV-2				5B	1		
154	pedal phalanx IV-2	x		juve	5A	1		
317	pedal phalanx IV-2	x		juve	7B	1		

152	pedal phalanx IV-3	x		juve	5A	1		
305	pedal phalanx IV-3		x	adult	7B	1		
15F-9	pedal phalanx IV-3	x		large juve		1		
15F-12	pedal phalanx IV-3	x		juve		1		
312	pedal phalanx IV-4		x	?adult	7A	1		
15F-11	pedal phalanx IV-4	x		large juve		1		
96	phalanx				3C			1
150E	phalanx, IV-3?			juve	7E	1		
63	post zygopopheses (2 sets), 1 set pre zygo, cauda				4C	1		
708	postorbital				8F	1		
Block 15-7	prearticular				7F?	1		
M11	premaxilla R	x		juve	5C	1		
993	premaxillary tooth				4G	1		
136b	premaxillary tooth				6E	1		
JB-1	premaxillary tooth				4B	1		
M5	premaxillary tooth			juve	5C	1		
150C	presacral? Vertebra				7E	1		
99	proximal chevron			juve	5B	1		
315	proximal chunk of podial				7A	1		
561	pubis				_H27		1	
333A	pubis L			small juv	4E	1		
15-1	quadrate	x		juve		1		
Block 15-1	quadrate				7F?	1		
144	radius			juve	7E	1		
306	radius				7B	1		
533	radius						1	
175	rib				7B	1		
304	rib					1		
726	rib		x	juve	6F	1		
1104	rib					1		
169b	rib				6C	1		
216 A and	rib or gastralia				5B	1		
127b	rib, cervical-dorsal transition				6E	1		
115	rib, distal end				5C	1		
127a	rib, mid dorsal				6E	1		
127c	rib, mid dorsal				6E	1		
134b	rib, mid dorsal				6D	1		
N-1	rib? Gastralia?			juve	5B	1		
122	R-maxilla	x		juve	6D	1		
192	sacrum				7B		1	
318 A	scapula				7-A		1	
15-2	splenial		x	juve		1		
Block 15-2	splenial				7F?	1		
169a	splenial or angular				6C	1		
145a	squamosal				7E			1
15-7	surangular		x	juve		1		
15F-52	tibia	x		juve		1		

934	tibia	small juv	4I	1		
1011	tooth		2G	1		
1102	tooth			1		
150A	tooth		7E	1		
15F-54	tooth	adult		1		
B-1	tooth		4B	1		
209b	ungual			1		
15A-10	ungual	?		1		
15F-23	ungual	juve	5B	1		
209 B	ungual		5B	1		
811 A	ungual		10G	1		
N-4	ungual	juve	5B	1		
1101	unident.			1		
1103	unident.			1		
150G	unidentified		7E	1		
150M	unidentified		7E	1		
M20	unidentified, cranial?		5C	1		
				223	18	6
