# Supplementary material for: Geology and taphonomy of a unique tyrannosaurid bonebed from the upper Campanian Kaiparowits Formation of southern Utah: implications for tyrannosaurid gregariousness
Source: PeerJ. 2021 Apr 19;9:e11013. doi: 10.7717/peerj.11013 (PMC8061582; doi:10.7717/peerj.11013)
Supplement: Supplemental Information 9 — Taphonomic indices are based on Behrensmeyer, 1991 and are given in values of 1-5. No 5 values were observed. Breakage patterns and the percent of area covered by fractures were both recorded. [file peerj-09-11013-s009.pdf]

Element N	Map Grid	Order	Family	Genus	Species
381-A	8C	Testudines	?Chelonioidea		
406	24_I	Testudines	?Neurankylus		
546	25_H	Osteichthyes	Acipenseriformes		
15L-6		Osteichthyes	Amiidae	Melvius	
541	26_I	Osteichthyes	Amiidae		
554	25_F	Osteichthyes	Amiidae	Melvius	sp.
H-17		Osteichthyes	Amiidae	Melvius	
H-13		Osteichthyes	Amiidae	Melvius	
464	24_H	Testudines	Baenidae		
444	25_H	Testudines	Baenidae		
308	7A	Testudines	Baenidae		
419-A	26_H	?Testudines	Chelonioidea		
400	27_G	Testudines	Chelonioidea		
511	24_G	Testudines	Chelonioidea		
732	9H	Testudines	Chelonioidea		
381-B	8C	Testudines	Chelonioidea		
433	25_H	Testudines	Chelonioidea?		
445	26_G	Dinosauria	Hadrosauridae		
473	24_G	Dinosauria	Hadrosauridae		
533	28_K	Dinosauria	Hadrosauridae		
557	26_H	Dinosauria	Hadrosauridae		
561	27_H	Dinosauria	Hadrosauridae		
318-A	7A	Dinosauria	Hadrosauridae		
561	27_H	Dinosauria	Hadrosauridae		
445-A	26_G	Dinosauria	Hadrosauridae		
411	25_I	Dinosauria	Hadrosauridae		
525	24_G	Dinosauria	Hadrosauridae		
549	26_H	Dinosauria	Hadrosauridae		
707	6F	Dinosauria	indet		
831		Osteichthyes	Lepisosteidae	Lepisosteus	
15C-F5	3E	Osteichthyes	Lepisosteidae		
467	24_H	Testudines	Neurankylus		
402	25_I	Dinosauria	Ornithischia		
192	7B	Dinosauria	Ornithischia		
321	8A	Dinosauria	Ornithischia		
559	27_H	Dinosauria	Ornithischia		
584	25_I	Testudines	Panchelonioidea		
515	24_G	Testudines	Panchelonioidea		
1205	24_I	Testudines	Panchelonioidea		
18-D-4	24_H	Testudines	Panchelonioidea		
441	25_H	Testudines	Panchelonioidea		
405	25_I	Testudines	Panchelonioidea		
327	3D	Testudines	Panchelonioidea		
471	24_H	Testudines	Panchelonioidea		
450	25_G	Testudines	Panchelonioidea		
1	7C	Dinosauria	Paraves		

97	3C	Dinosauria	Paraves		
158	4D	Dinosauria	Paraves		
791	6F	Dinosauria	Paraves		
792	6F	Dinosauria	Paraves		
145-A	7E	Dinosauria	Paraves		
49-A	7D	Dinosauria	Paraves		
70-A	7C	Dinosauria	Paraves		
443	25_H	Osteichthyes	Rhinopristiformes?		
713	9G	Osteichthyes	Teleostei		
J-17-3-7/7	9G	Osteichthyes	Teleostei		
847	5G	Dinosauria	Theropoda		
333-A	4E	Dinosauria	Theropoda		
373	8D	Dinosauria	Theropoda	indet	
740-A	10G	Testudines	Trionychidae		
212	5B	Dinosauria	Tyrannosauridae	Teratophoneus	cf. curriei
39-A	4B		Tyrannosauridae	Teratophoneus	cf. curriei
13	6C	Dinosauria	Tyrannosauridae	Teratophoneus	cf. curriei
14	6C	Dinosauria	Tyrannosauridae	Teratophoneus	cf. curriei
20	5C	Dinosauria	Tyrannosauridae	Teratophoneus	cf. curriei
21	5B	Dinosauria	Tyrannosauridae	Teratophoneus	cf. curriei
22	5B	Dinosauria	Tyrannosauridae	Teratophoneus	cf. curriei
24	4B	Dinosauria	Tyrannosauridae	Teratophoneus	cf. curriei
30	5B	Dinosauria	Tyrannosauridae	Teratophoneus	cf. curriei
31	4B	Dinosauria	Tyrannosauridae	Teratophoneus	cf. curriei
35	4B	Dinosauria	Tyrannosauridae	Teratophoneus	cf. curriei
37-A	4B	Dinosauria	Tyrannosauridae	Teratophoneus	cf. curriei
37-B	4B	Dinosauria	Tyrannosauridae	Teratophoneus	cf. curriei
39-B	4B	Dinosauria	Tyrannosauridae	Teratophoneus	cf. curriei
59	5B	Dinosauria	Tyrannosauridae	Teratophoneus	cf. curriei
60	4B	Dinosauria	Tyrannosauridae	Teratophoneus	cf. curriei
63	4C	Dinosauria	Tyrannosauridae	Teratophoneus	cf. curriei
74	6D	Dinosauria	Tyrannosauridae	Teratophoneus	cf. curriei
98	4B	Dinosauria	Tyrannosauridae	Teratophoneus	cf. curriei
99	5B	Dinosauria	Tyrannosauridae	Teratophoneus	cf. curriei
109	5A	Dinosauria	Tyrannosauridae	Teratophoneus	cf. curriei
115	5C	Dinosauria	Tyrannosauridae	Teratophoneus	cf. curriei
116	5C	Dinosauria	Tyrannosauridae	Teratophoneus	cf. curriei
118	5C	Dinosauria	Tyrannosauridae	Teratophoneus	cf. curriei
127	6E	Dinosauria	Tyrannosauridae	Teratophoneus	cf. curriei
130	5D	Dinosauria	Tyrannosauridae	Teratophoneus	cf. curriei
132	5D	Dinosauria	Tyrannosauridae	Teratophoneus	cf. curriei
134	6D	Dinosauria	Tyrannosauridae	Teratophoneus	cf. curriei
143	7D	Dinosauria	Tyrannosauridae	Teratophoneus	cf. curriei
144	7E	Dinosauria	Tyrannosauridae	Teratophoneus	cf. curriei
150-C	7E	Dinosauria	Tyrannosauridae	Teratophoneus	cf. curriei
150-E	7E	Dinosauria	Tyrannosauridae	Teratophoneus	cf. curriei
150-N	7E	Dinosauria	Tyrannosauridae	Teratophoneus	cf. curriei

151	7E	Dinosauria	Tyrannosauridae	Teratophoneus	cf. curriei
152	5A	Dinosauria	Tyrannosauridae	Teratophoneus	cf. curriei
153	5A	Dinosauria	Tyrannosauridae	Teratophoneus	cf. curriei
154	5A	Dinosauria	Tyrannosauridae	Teratophoneus	cf. curriei
164	6C	Dinosauria	Tyrannosauridae	Teratophoneus	cf. curriei
169	6C	Dinosauria	Tyrannosauridae	Teratophoneus	cf. curriei
196	8C	Dinosauria	Tyrannosauridae	Teratophoneus	cf. curriei
197	8C	Dinosauria	Tyrannosauridae	Teratophoneus	cf. curriei
202	7B	Dinosauria	Tyrannosauridae	Teratophoneus	cf. curriei
208	5A	Dinosauria	Tyrannosauridae	Teratophoneus	cf. curriei
211	5B	Dinosauria	Tyrannosauridae	Teratophoneus	cf. curriei
217	6C	Dinosauria	Tyrannosauridae	Teratophoneus	cf. curriei
300	7A	Dinosauria	Tyrannosauridae	Teratophoneus	cf. curriei
303	7A	Dinosauria	Tyrannosauridae	Teratophoneus	cf. curriei
316	7B	Dinosauria	Tyrannosauridae	Teratophoneus	cf. curriei
317	7B	Dinosauria	Tyrannosauridae	Teratophoneus	cf. curriei
347	7E	Dinosauria	Tyrannosauridae	Teratophoneus	cf. curriei
352	8E	Dinosauria	Tyrannosauridae	Teratophoneus	cf. curriei
362	6C	Dinosauria	Tyrannosauridae	Teratophoneus	cf. curriei
369	7B	Dinosauria	Tyrannosauridae	Teratophoneus	cf. curriei
394	8F	Dinosauria	Tyrannosauridae	Teratophoneus	cf. curriei
395	8F	Dinosauria	Tyrannosauridae	Teratophoneus	cf. curriei
437	25_G	Dinosauria	Tyrannosauridae	Teratophoneus	cf. curriei
589-A	25_I	Dinosauria	Tyrannosauridae	Teratophoneus	cf. curriei
701	3D	Dinosauria	Tyrannosauridae	Teratophoneus	cf. curriei
702	6F	Dinosauria	Tyrannosauridae	Teratophoneus	cf. curriei
705	6F	Dinosauria	Tyrannosauridae	Teratophoneus	cf. curriei
719	7F	Dinosauria	Tyrannosauridae	Teratophoneus	cf. curriei
721-A	6F	Dinosauria	Tyrannosauridae	Teratophoneus	cf. curriei
728	6F	Dinosauria	Tyrannosauridae	Teratophoneus	cf. curriei
761	9F	Dinosauria	Tyrannosauridae	Teratophoneus	cf. curriei
934	4G	Dinosauria	Tyrannosauridae	Teratophoneus	cf. curriei
1104	6E	Dinosauria	Tyrannosauridae	Teratophoneus	cf. curriei
1207	24_I	Dinosauria	Tyrannosauridae	Teratophoneus	cf. curriei
209-B	5B	Dinosauria	Tyrannosauridae	Teratophoneus	cf. curriei
216-A & B	5B	Dinosauria	Tyrannosauridae	Teratophoneus	cf. curriei
58-A	5B	Dinosauria	Tyrannosauridae	Teratophoneus	cf. curriei
811-A	10G	Dinosauria	Tyrannosauridae	Teratophoneus	cf. curriei
B-1	4B	Dinosauria	Tyrannosauridae	Teratophoneus	cf. curriei
B-2	4B	Dinosauria	Tyrannosauridae	Teratophoneus	cf. curriei
B-7	4B	Dinosauria	Tyrannosauridae	Teratophoneus	cf. curriei
Block 15-1	7F	Dinosauria	Tyrannosauridae	Teratophoneus	cf. curriei
Block 15-2	7F	Dinosauria	Tyrannosauridae	Teratophoneus	cf. curriei
Block 15-7	7F	Dinosauria	Tyrannosauridae	Teratophoneus	cf. curriei
M11	5C	Dinosauria	Tyrannosauridae	Teratophoneus	cf. curriei
M12	5C	Dinosauria	Tyrannosauridae	Teratophoneus	cf. curriei
M14	5C	Dinosauria	Tyrannosauridae	Teratophoneus	cf. curriei

M15	5C	Dinosauria	Tyrannosauridae	Teratophoneus	cf. curriei
M6	5C	Dinosauria	Tyrannosauridae	Teratophoneus	cf. curriei
M7	5C	Dinosauria	Tyrannosauridae	Teratophoneus	cf. curriei
16G-20	8E	Dinosauria	Tyrannosauridae	Teratophoneus	cf. curriei
150-H	7E	Dinosauria	Tyrannosauridae	Teratophoneus	cf. curriei
15F-6	5B	Dinosauria	Tyrannosauridae	Teratophoneus	cf. curriei
15F-3	5B	Dinosauria	Tyrannosauridae	Teratophoneus	cf. curriei
15F-18	5B	Dinosauria	Tyrannosauridae	Teratophoneus	cf. curriei
15F-15	5B	Dinosauria	Tyrannosauridae	Teratophoneus	cf. curriei
15F-12	5B	Dinosauria	Tyrannosauridae	Teratophoneus	cf. curriei
15F-11	5B	Dinosauria	Tyrannosauridae	Teratophoneus	cf. curriei
15F-9	5B	Dinosauria	Tyrannosauridae	Teratophoneus	cf. curriei
15F-39	5B	Dinosauria	Tyrannosauridae	Teratophoneus	cf. curriei
15F-38	5B	Dinosauria	Tyrannosauridae	Teratophoneus	cf. curriei
15F-35	5B	Dinosauria	Tyrannosauridae	Teratophoneus	cf. curriei
15F-8	5B	Dinosauria	Tyrannosauridae	Teratophoneus	cf. curriei
15F-50	5B	Dinosauria	Tyrannosauridae	Teratophoneus	cf. curriei
15F-52	5B	Dinosauria	Tyrannosauridae	Teratophoneus	cf. curriei
15F-36	5B	Dinosauria	Tyrannosauridae	Teratophoneus	cf. curriei
15F-45	5B	Dinosauria	Tyrannosauridae	Teratophoneus	cf. curriei
15F-1	5B	Dinosauria	Tyrannosauridae	Teratophoneus	cf. curriei
15F-2	5B	Dinosauria	Tyrannosauridae	Teratophoneus	cf. curriei
15F-3	5B	Dinosauria	Tyrannosauridae	Teratophoneus	cf. curriei
15F-53	5B	Dinosauria	Tyrannosauridae	Teratophoneus	cf. curriei
15F-23	5B	Dinosauria	Tyrannosauridae	Teratophoneus	cf. curriei
209-A	5B	Dinosauria	Tyrannosauridae	Teratophoneus	cf. curriei
175	7B	Dinosauria	Tyrannosauridae	Teratophoneus	cf. curriei
213	5B	Dinosauria	Tyrannosauridae	Teratophoneus	cf. curriei
15F-16	5B	Dinosauria	Tyrannosauridae	Teratophoneus	cf. curriei
15F-61	5B	Dinosauria	Tyrannosauridae	Teratophoneus	cf. curriei
15F-13	5B	Dinosauria	Tyrannosauridae	Teratophoneus	cf. curriei
86	5B	Dinosauria	Tyrannosauridae	Teratophoneus	cf. curriei
96	3C	Dinosauria	Tyrannosauridae?	Teratophoneus	cf. curriei
775	9G	Dinosauria?			
18D-1	23_H	indet			
189	4D	Osteichthyes			
834	5G	Osteichthyes			
15C-F1	3E	Osteichthyes			
15C-F2	3E	Osteichthyes			
15C-F3	3E	Osteichthyes			
15C-F4	3E	Osteichthyes			
589-B	25_I	Osteichthyes			
466	27_I	Osteichthyes			
419-B	26_H	Osteichthyes			
76	7C	Testudines			
185	4D	Testudines			
188	7C	Testudines			

386	4E	Testudines
494	24_G	Testudines
530	25_G	Testudines
536	23_G	Testudines
71	7C	Testudines
79	3B	Testudines
919	4H-3H	Testudines
396-A	6E	Testudines
744-B	10G	Testudines
78-A	7_A	Testudines
82-B	7D	Testudines
M17	5C	Testudines
825	6G	Testudines?

Element	Right/Left	Onto Stage	Notes	Azimuth	Taphonom	Dominant	Fracture C	Notes
limb		huge taxon		290	1	1,2,4	50	prep damage to corte
costal					2			
cranial					1	1,2	45	
					1			
dentary					1	1,2	65	
centrum					2			juvenile bone texture,
					2			
					2			
carapace fragment					1			
carapace fragment					1			
shell		small	fragments		2	2	45	
vertebra					2	1,4	60	
tibia	right	giant			1	4	30	
limb					1	4	30	
limb		giant			1	1,4	30	
					2	4	40	only evaluated the
					1	1,2,4	50	
lateral metacarpal?		subadult			1	1	10	
caudal		subadult/adult			1	4	15	
radius					1			
chevron		juvenile			1	1	40	distal end shattered
pubis		juvenile			1	1	40	rectangular checkerbo
right scapula		juvenile			1			
pubis		juvenile			1			
metacarpal		juvenile			1			
mid to distal caudal v		subadult/adult			2	4	30	
centrum					2	4	50	trans and long fractur
centrum		juvenile			2	4	60	
					1	1	15	
dorsal vertebrae					1			
centrum					1			cannot assess domina
costal					1			
					2			
?					2			
					4	1		
ischial shaft?					4	4	60	
humerus					1			
paired dentary					1			
carapace fragment					2			
carapace fragment					2			
carapace fragment					2			
marginal					2			
marginal					2			
marginal					2			
marginal					2			
tibia?					1	1	7.5	

II-1 phalan	right				1		1	15	some spalling
angular	left				1		1	5	
medial phalanx					1		1	5	
					1	1,2		60	
squamosal	right		associated with other		1			0	
				305	1		1	40	
Pedal phalanx II-1 or II-2					1		4	60	crushed in center
skull plate?					1	1 and irreg		60	
dorsal vertebrae					1				
articulated fish vertebrae, fin rays					1				cannot assess domina
limb				271	1	1,2,4		70	
		juvenile			1	1,4		30	
limb shaft		small indiv	next to 370		2	1,2		60	
pectoral girdle					1		1	5	
pedal phalanx					1				
caudal					1				
dorsal rib		subadult/adult			1		1	10	
dorsal rib		subadult	mid-shaft s	240	1		1	10	
dorsal rib			in Block M	305	1	1,2		40	
pedal phal	right	adult		225	1		1	7	
pedal phal	right	adult	only anterior half		1	1, 2		25	60% trans, 40% long
femur		juvenile	mate of 39	355	1		1	50	
pedal phal	right	juvenile	removed from jacket		1	1,4		5	
distal caudal		juvenile/su	from jacket B probabl		1		1	5	
distal caudal		juvenile/su	south of jacket B		1		1	7	
distal caudal					1				
distal caudal					1				
caudal					1				
metarsal		adult			1		1		
distal caudal centra (h		subadult			1			0	
2 sets post zygopopheses, 1 set p			map shows associate		1			40	
?post orbi	right	juvenile	fragment from jacket		1		1	60	
mid to distal caudal v		subadult	Given number C2 dur		1		4	70	
proximal chevron		juvenile	Scratches are present		1			0	No discernable fractu
chevron		adult			1		1	5	Lots of root damage c
rib, distal end		subadult	in Block M	270	1		1	5	
gastralia			in Block M		1		1	10	
gastralia		subadult		315	1		1	5	
gastralia? dorsal ribs?				355	1		1	40	
pedal phal	left	juvenile			1		1	10	
cervical neural arch		subadult	C7 or C8		1	1,2,3		90	checkerboard
gastralia? Rib?				360	1		1	20	
dorsal rib		juvenile			1		1	40	
radius	left	juvenile			1		1	15	
dorsal vertebrae					1		1		
pedal phalanx		subadult			1		1		
dentary	left	juvenile			1		1		

dorsal rib					1		1	*		
pedal phal	right	juvenile	in jacket 15A		1		4		5	
gastralia		adult	in jacket 1!	290	1		1		25	
IV-2	left	subadult	in jacket 15A		1		1		30	
dorsal rib		subadult/a	near 362		1		1		10	
gastralium					1		1		25	
dorsal rib		adult	mid shaft (	337	1		1		30	
gastralia		juvenile			1		1			
mid-dorsal rib		subadult/adult		293	1	1,2			30	
pedal phal	right	juvenile			1		4		50	
pedal phalanx			in Jacket N or Block N		1		4		40	
dorsal rib		adult			1		1			
dentary ar	right	adult	strongly weathered		1	1,2			60	Checkerboard fractur
proximal end lateral r		adult	rest of element is in b		1		1		5	Noticeable scribe and
pedal phal	left	adult			1	1,3			30	
phalanx					1		2		5	
dorsal rib		subadult/a	shaft and frags		1		2		40	Primarily longitudinal,
gastralia? Cranial?					1		1		30	
dorsal rib		adult	fragment		1		1		5	Root damage to corte
rib					1	*				
femur	left	juvenile	mate of 24	205	1		1		65	
nasal		adult	almost cor	317	1	1,2			50	Bone is rugose and di
distal caudal		subadult			1		1		5	
cervical rib					1		1			
caudal? Centrum					1		4		80	small checkerboard fr
dorsal vertebrae					1	1,2,4			90	small checkerboard fr
dorsal rib					1		1		20	
palatine					1	See notes			85	crushed
maxilla	left	subadult			1					
pedal phalanx		yearling	smallest individual ty		1	2?			5	very small "chipped" f
braincase		subadult			1					
?		juvenile		357	1	1,4			30	
dorsal rib		subadult			1		1			
tibia					1					
ungual					1		1		5	
rib or gastralia		subadult	fragment from jacket		1	1, 2			50	
gastralium			in Block M		1		1		30	
ungual					1		1		15	some spalling
tooth					1		1		5	
caudal					1		1		5	
distal caudal					1		1		5	
quadrate			Could this be block 1!		1				70	irregular fracture patt
splenial			Could this be block 1!		1				30	irregular fracture patt
prearticular			Could this be block 1!		1				40	irregular fracture patt
premaxilla	right	juvenile			1					
gastralium					1		1		20	
lacrimal					1				65	irregular fracture patt

chevron			1	1	20	
ectopterygoid?			1	see notes	50	irregular fracture patt
gastralium?			1	1	20	
atlas-axis			1			
pedal phalanx III-1			1			
gastralia			1			
gastralia			1			
gastralia			1			
phalanx II- left			1			
phalanx II-3			1			
phalanx IV-4			1			
phalanx IV-3			1			
proximal chevron			1			
gastralia	adult		1			
phalanx II-1			1			
phalanx IV-1			1			
distal chevron			1			
tibia_astragalus_calca	juvenile		1			
fibula			1			
metatarsal	adult		1			
metatarsal	juvenile		1			
metatarsal	juvenile		1			
metatarsal	juvenile		1			
metatarsal	juvenile		1			
ungual			1		0	no visible fractures, di
pedal phalanx			1			
rib		fragment associated v	2	1	5	
pedal phalanx		in Jacket N or Block N	2	1, 4	15	
ungual			2			
metatarsal	adult		2			
dorsal rib			3			
pedal phalanx		in Jacket N or Block N	1	4	20	
phalanx			1	1	40	
			4	1,4	80	cortical surface very d
			1	1	30	crushed and fragment
fish skull plate?			1	1,2	60	checkerboard
cranial?			1		30	cannot assess domina
skull			1	1,2	25	
skull			1	1	10	
skull			1		40	cannot assess domina
skull			1		40	cannot assess domina
skull plate ?			1		25	cannot assess domina
skull			2		35	cannot assess domina
	juvenile		2	1	60	
metapodial		medium size taxon	1	4?	10	spalling may be surfac
tibia		medium size	1	4	50	
femur		medium size-shaft on	1	4	20	

limb shaft, tibia?				1	4	50	
tibia				1	4	20	
costal				1			
limb shaft				1			
tibia		medium size taxon		2	1,2	60	
tibia	adult	large species		2	4	70	
scapula			340	2		0	irregular fracture patt
limb bone				2	4	50	
limb				2	4	50	
phalanx				2	4	60	
phalanx				2	4	60	
coracoid				2	1	60	
limb bone				2	4	30	large transverse fractu

Labels
0
1
2
3
4
5

Bin	*Frequency*
0	0
1	161
2	35
3	1
4	3
5	0
Total	200

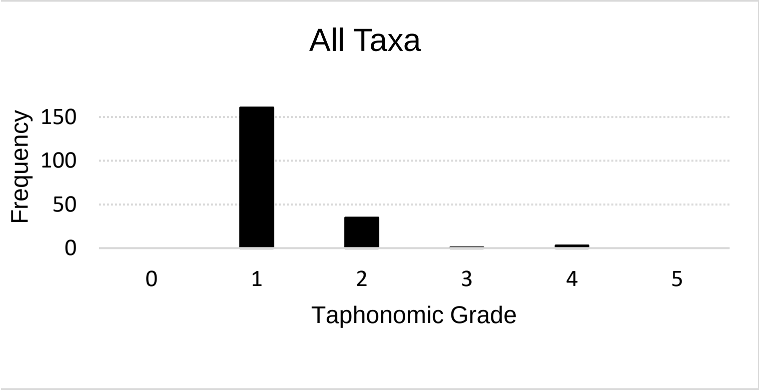

ɔard, dull, maybe not cortical surface
